# Estimated reductions in cardiovascular and gastric cancer disease burden through salt policies in England: an IMPACT_NCD_ microsimulation study

**DOI:** 10.1136/bmjopen-2016-013791

**Published:** 2017-01-24

**Authors:** Chris Kypridemos, Maria Guzman-Castillo, Lirije Hyseni, Graeme L Hickey, Piotr Bandosz, Iain Buchan, Simon Capewell, Martin O'Flaherty

**Affiliations:** 1 Department of Public Health and Policy, University of Liverpool, Liverpool, UK; 2 Department of Biostatistics, University of Liverpool, Liverpool, UK; 3 Department of Prevention and Medical Education, Medical University of Gdansk, Gdansk, Poland; 4 Farr Institute @ HeRC, University of Manchester, Manchester, UK

**Keywords:** Salt, Cardiovascular disease, Gastric Cancer, Public health policy, Microsimulation

## Abstract

**Objective:**

To estimate the impact and equity of existing and potential UK salt reduction policies on primary prevention of cardiovascular disease (CVD) and gastric cancer (GCa) in England.

**Design:**

A microsimulation study of a close-to-reality synthetic population. In the first period, 2003–2015, we compared the impact of current policy against a counterfactual ‘no intervention’ scenario, which assumed salt consumption persisted at 2003 levels. For 2016–2030, we assumed additional legislative policies could achieve a steeper salt decline and we compared this against the counterfactual scenario that the downward trend in salt consumption observed between 2001 and 2011 would continue up to 2030.

**Setting:**

Synthetic population with similar characteristics to the non-institutionalised population of England.

**Participants:**

Synthetic individuals with traits informed by the Health Survey for England.

**Main measure:**

CVD and GCa cases and deaths prevented or postponed, stratified by fifths of socioeconomic status using the Index of Multiple Deprivation.

**Results:**

Since 2003, current salt policies have prevented or postponed ∼52 000 CVD cases (IQR: 34 000–76 000) and 10 000 CVD deaths (IQR: 3000–17 000). In addition, the current policies have prevented ∼5000 new cases of GCa (IQR: 2000–7000) resulting in about 2000 fewer deaths (IQR: 0–4000). This policy did not reduce socioeconomic inequalities in CVD, and likely increased inequalities in GCa. Additional legislative policies from 2016 could further prevent or postpone ∼19 000 CVD cases (IQR: 8000–30 000) and 3600 deaths by 2030 (IQR: −400–8100) and may reduce inequalities. Similarly for GCa, 1200 cases (IQR: −200–3000) and 700 deaths (IQR: −900–2300) could be prevented or postponed with a neutral impact on inequalities.

**Conclusions:**

Current salt reduction policies are powerfully effective in reducing the CVD and GCa burdens overall but fail to reduce the inequalities involved. Additional structural policies could achieve further, more equitable health benefits.

**Strengths and limitations of this study**

- Our study uses a technically advanced dynamic microsimulation model that synthesises information from the best available sources of information on population exposures to salt, and other non-communicable disease-related risk factor.
- Many assumptions must be made with such models; yet, in spite of the potential frailty of such assumptions this model validated well against observed cardiovascular disease and gastric cancer incidence and mortality in real populations, even when multiply stratified.
- The main assumption for the evaluation of current policy was that the decline in salt consumption observed since 2003 was fully attributable to the implemented policy.
- We could not find a sufficiently large data set with individual-level 24-hour urine sodium measurements and other non-communicable disease-related risk factor information. Therefore, we developed a stochastic process to overcome this and synthesise information from multiple sources, which increased the overall uncertainty of the model and is reflected in our reported uncertainty estimates.
- To ensure transparency, we have made IMPACT_NCD_ source code open under GNU GPLv3 licence.

## Background

Excess salt consumption is associated with higher risk of cardiovascular disease (CVD) and gastric cancer (GCa).^1^ ^2^ Globally, more than 1.5 million CVD-related deaths every year can be attributed to the excess salt intake.^3^ Further salt-related deaths come from GCa. Health policies worldwide, therefore, aim to reduce dietary salt intake.^4^ Furthermore, the WHO recommends reducing population exposure to salt as one of the ‘best buy’ strategies to prevent non-communicable diseases, highlighting its cost-effectiveness and feasibility.^5^

Since 2003, the UK has had one of the world's most successful salt reduction strategies, including public awareness campaigns, food labelling and ‘voluntary’ reformulation of processed foods.^6^ The strategy components and the evolution of the strategy over the years have been described in detail elsewhere.^7^ ^8^ This package of measures is regularly evaluated and has been monitored through nationally representative surveys using 24-hour urine collection measurements.^9^ Between 2001 and 2011, the mean salt consumption in the UK dropped from 9.5 to 8.1 g/day^10^—a success, however, still far from the national target of 6 g/day.^11^

In the UK, salt consumption is higher in more deprived groups.^12^ ^13^ Therefore, interventions aiming to reduce salt consumption should ideally aim to also reduce socioeconomic inequalities in health. Unfortunately, the current UK strategy might potentially increase socioeconomic inequality because awareness campaigns, food labelling and voluntary reformulation can be more effective among the more health conscious, affluent individuals.^14–17^ Indeed, evidence suggests the socioeconomic gradient in salt consumption might have worsened during the programme.^13^ ^18^ In contrast, modelling studies consistently suggest that more structural interventions can be more effective, cost-effective and equitable than the current UK policy.^19^ ^20^

Structural salt reduction policies are usually based on legislative initiatives like a mandatory reformulation of processed foods or taxation of high-salt foods. Such policies have already been adopted successfully in Argentina, South Africa, Portugal, Hungary and elsewhere, emphasising their feasibility.^4^ In fact, the actual number of countries currently implementing legislative measures has substantially increased since 2010, indicating a global move towards stricter salt reduction policies.^4^

The aim of this study was to estimate the impact and equity of current UK salt reduction policy on CVD and GCa burden since 2003. We further compared current policy with other feasible policies to estimate possible additional incidence and mortality reductions.

## Methods

We used IMPACT_NCD_, a discrete time, dynamic, stochastic microsimulation model to simulate the effect of current policy and compare it to counterfactual scenarios. We split our analysis into two periods. The first corresponds to years 2003–2015, for which we compared the potential benefits of current policies against a null intervention scenario. For the second period, 2016–2030, we explored the potential benefits of additional structural salt reduction policies, assuming they might lead to steeper declines in salt intake.

### Model description

IMPACT_NCD_ simulates synthetic individuals and allows for greater flexibility and more detailed simulation, including different lag times between exposures and outcomes, socioeconomic gradients in trends of risk factors and a competing risk framework—a computationally intensive task for which we employed the Farr Institute's statistical high-performance computing facilities.^21^

The model synthesises information from Office for National Statistics (ONS) regarding English population structure by age, sex and socioeconomic status and the Health Survey for England^22^ regarding exposure to CVD and GCa-associated risk factors (see below) to generate a close-to-reality synthetic population.^23^ Well-established causal pathways between associated risk factors and disease are used to translate exposure into CVD and GCa incidence and mortality, in a competing risk framework. Effect sizes were taken from published meta-analyses and longitudinal studies (see online supplementary table S1). For salt, we assumed a mediated effect through systolic blood pressure on CVD incidence with 5-year mean lag time, and a direct effect on GCa incidence with a mean lag time of 8 years.

10.1136/bmjopen-2016-013791.supp1 supplementary data

Outputs include CVD and GCa incidence and mortality in the synthetic population under different scenarios. A detailed description of IMPACT_NCD_ is provided in the online supplementary Chapters S2-S4.

#### Risk factor modelling

The exposure of the synthetic population to salt was informed by four nationally representative surveys employing 24-hour urine collections between 2001 and 2011.^10^ ^24–26^ We used a stochastic process to enhance the information from these surveys with information from spot urine measurements (see detailed description in the online supplement Paragraph S3.3.2). Then, we used quantile regression to project daily salt consumption to 2030. Changes in salt consumption were transformed to systolic blood pressure changes using the metaregression equation of a meta-analysis of 103 trials.^3^ The ideal level of salt consumption is not clear (see appendix Text S4 in Mozaffarian *et al*).^3^ We allowed the level of ideal salt consumption under which no risk exist to vary between 1.5 and 6 g/day with a mode of 3.8 g/day, following a PERT distribution.^27^

Trends of other CVD and GCa-associated risk factors were also considered in this study by projecting the observed in Health Survey for England trends since 2001, up to 2030. For CVD, body mass index, total plasma cholesterol, diabetes mellitus (diagnosis or elevated glycated haemoglobin/no diabetes), smoking status (current/ex/never smoker), environmental tobacco exposure (binary variable), fruit and vegetable (portions/day) consumption, and physical activity (days with at least 30 min of moderate or vigorous physical activity/week) were included. Smoking duration, body mass index, and less than two portions of fruit and vegetable consumption were considered for GCa.^28^

CVD was defined as the sum of coronary heart disease (CHD) and stroke (any type) cases. This study focuses on primary prevention; hence, only the first episode of CHD, stroke and GCa was considered. The competing risk framework allows individuals to develop CHD, stroke or GCa independently, and die from these or any other cause.

#### Model outputs

For this study, IMPACT_NCD_ estimated the cumulative cases prevented or postponed and deaths prevented or postponed for the relevant period and for ages 30–84. The results were stratified by quintile groups of Index of Multiple Deprivation (QIMD), a relative measure of area deprivation widely used in England.^29^ Inspired by the slope index of inequality,^30^ we used two regression-based metrics, the ‘absolute equity slope index’ and the ‘relative equity slope index’, as equity measures of a policy. The former measures the impact of an intervention on absolute inequality; for instance, a value of 100 means 100 more cases were prevented or postponed in most deprived compared with least deprived areas, and absolute inequality was decreased. The latter takes into account pre-existing socioeconomic gradient of disease burden and measures the impact of an intervention on relative inequality; positive values mean the policy tackles relative inequality and negative that the policy generates relative inequality.

Owing to the assumed lag times, any changes in salt exposure in the 2003 to 2015 period will reflect on CVD incidence and mortality in years 2008 to 2020, and GCa incidence and mortality in years 2011–2023. Similarly, for the period 2016–2030, these changes will be reflected in CVD burden in 2021–2035 and in GCa burden in 2024–2038.

#### Uncertainty analysis

A probabilistic sensitivity analysis is incorporated in our estimates, as IMPACT_NCD_ implements a second-order Monte Carlo approach that allows the estimated uncertainty of model inputs to be propagated to the outputs.^31^ We summarise the output distributions by reporting medians and IQRs in the form of first and third quartiles. We also report the probability (Ps) that a policy scenario aspect is superior to the counterfactual one. For example, ‘100 cases prevented or postponed (Ps=80%) in scenario A’ is interpreted as ‘in 80% of Monte Carlo iterations, at least one case has been prevented or postponed in scenario “A” comparing to the counterfactual scenario’. Consequently, in the remaining 20% of iterations, cases in scenario ‘A’ were more than in the counterfactual scenario. This does not mean that scenario ‘A’ was harmful, but that its effect in those particular settings was not large enough to exceed the ‘noise level’ from other sources of uncertainty in the model. For a detailed description of the sources of uncertainty that were considered, please refer to the online supplement Chapter S6.

### Period 2003–2015 scenarios

Two scenarios were simulated. The ‘no intervention’ scenario assumes that no salt-related interventions were implemented since 2003. Therefore, the salt exposure remained stable at the estimated level of 2003 for the period up to 2015. The ‘current policy’ scenario simulates the decline in salt consumption that was observed between 2003 and 2011, and projects it up to 2015, assuming a logarithmic decline.

### Period 2016–2030 scenarios

Here, we modelled the potential effect of structural, legislative policies on salt intake, aimed to achieve feasible and ideal targets. First, we modelled a ‘current policy’ (baseline) scenario where the logarithmic decline observed from 2003 to 2011 was projected up to 2030.

In a ‘feasible’ target scenario, we assumed that in 2016, policies like mandatory reformulation and/or taxation of high-salt foods were implemented and as a result, the mean salt consumption will gradually decline to the national target of 6 g/day by 2020 for ages 19 to 64. Owing to lack of empirical evidence regarding the magnitude of the impact of such policies on salt, we allowed their target to vary between 5.8 and 7 g/day following a PERT distribution. The intervention was modelled to be more effective for individuals with higher salt consumption.

**Figure 1:**
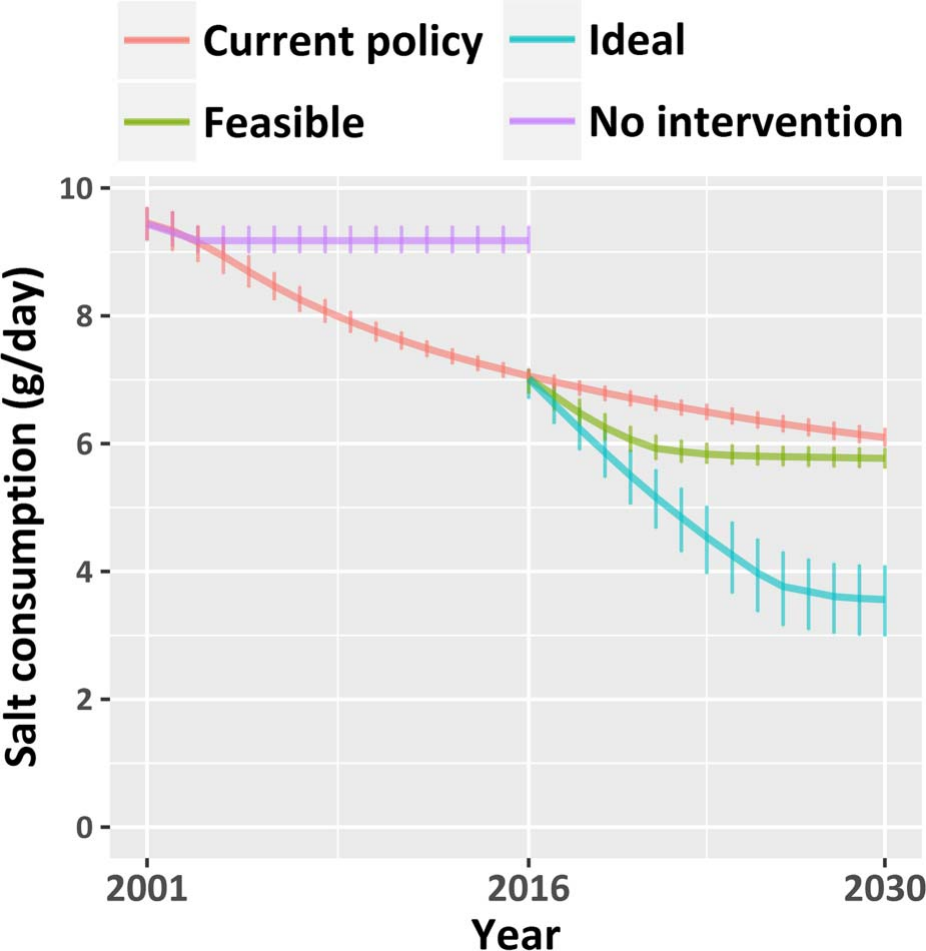
Modelled trends of median salt consumption in English population aged 30–84 under the four simulated scenarios. Error bars represent IQRs.

In an ‘ideal’ target scenario, we assumed mean salt intake to reach the ideal salt intake 3.8 g/day by 2025 for ages 19–64. The ideal salt consumption was modelled to vary between 1.5 and 6 g/day following a PERT distribution. Similarly to the previous scenario, the intervention was modelled to be more effective for individuals with higher salt consumption. The modelled trends of salt consumption for all scenarios are depicted in figure 1.

#### Other assumptions

We assumed that CVD and GCa case fatality is improving by 5% and 2% annually, respectively, but the rate of improvement diminishes by 1% (relative) every year. Moreover, we assumed that there is a constant fatality rate socioeconomic gradient of ∼5% by QIMD level (halved for ages over 70) forcing the more deprived to experience worse disease outcomes. These assumptions are based on empirical evidence.^32–35^ Table 1 presents the key modelling assumptions.

**Table 1:** IMPACT_NCD_ key assumptions

Population module	Migration is not considered.
	Social mobility is not considered.
	QIMD is a marker of relative area deprivation with several versions since 2003. We considered all version of QIMD identical.
	We assume all salt that is consumed is excreted from urine and all urine sodium comes from salt consumption.
	We assume that the surveys used are truly representative of the population.
	We assume that the decline in salt consumption observed since 2003 was fully attributable to the implemented policy.
Disease module	We assume multiplicative risk effects.
	We assume log-linear dose–response for the continuous risk factors.
	We assume that the effects of the risk factors on incidence and mortality are equal and risk factors are not modifying survival.
	We assume 5-year mean lag time for CVD and 8-year for GCa (except for the cumulative effect of smoking on GCa where lag was assumed similar to CVD one).
	We assume 100% risk reversibility.
	We assume that trends in disease incidence are attributable only to trends of the relevant modelled risk factors.
	Only well-accepted associations between upstream and downstream risk factors that have been observed in longitudinal studies are considered. However, the magnitudes of the associations are extracted from a series of nationally representative cross-sectional surveys (Health Survey for England).
	For GCa, we assume that survival of 10 years after diagnosis equals remission.

CVD, cardiovascular disease; GCa, gastric cancer; QIMD, quantile group of Index of Multiple Deprivation.

## Results

We present our results separately for the two distinct periods, then an external validation of IMPACT_NCD_.

### Evaluation of current policy (2003–2015)

Under the ‘current policy’ scenario, median salt consumption was reduced from 8.9 (IQR: 8.7–9.2) g/day in 2003 to 7.1 (IQR: 6.9–7.2) g/day in 2015. Socioeconomic inequalities in salt consumption remained and might even have increased as a result of the current policy.

Under the ‘no intervention’ scenario, IMPACT_NCD_ estimated ∼1.3 (IQR: 1.2–1.4) million new cases of CVD and 700 000 (IQR: 680 000–720 000) deaths from CVD. Likewise, the model estimated ∼68 000 (IQR: 61 000–74 000) new GCa cases and 41 000 (IQR: 37 000–44 000) deaths.

Compared with the ‘no intervention’ scenario, the salt reduction strategy resulted in about 52 000 (IQR: 34 000–76 000; Ps=99%) fewer new CVD cases, and 10 000 (IQR: 3000–17 000; Ps=86%) fewer CVD deaths. In addition, the current policy prevented around 5000 (IQR: 2000–7000; Ps=92%) new cases of GCa resulting in 2000 (IQR: 0–4000; Ps=78%) fewer GCA deaths.

When equity was considered, we estimated that the current policy has a rather neutral effect on tackling socioeconomic inequalities in CVD. The effect on GCa equity was more complex. Current policy apparently prevented or postponed fewer GCa cases in more deprived areas. However, GCa incidence increases with age and more affluent individuals tend to live longer. After directly standardising age and sex, the effect was essentially disappeared for absolute inequality bur remained for relative inequality (table 2).

**Table 2:** Effectiveness of current policy compared with the ‘no intervention’ scenario by quantile group of Index of Multiple Deprivation (QIMD)

	CPP absolute reduction in thousands		CPP relative percentage reduction	
QIMD	CVD	GCa	CVD	GCa
1 (least deprived)	9.7 (4.6 to 16.2)	1.0 (−0.1 to 2.1)	4.1% (1.9% to 6.5%)	7.3% (−0.9% to 15.3%)
2	11.7 (5.5 to 18.8)	1.1 (0.0 to 2.3)	4.4% (2.3% to 6.8%)	7.8% (0.0% to 16.1%)
3	11.3 (5.3 to 17.8)	1.0 (−0.2 to 2.0)	4.3% (2.2% to 6.4%)	6.9% (−1.3% to 14.7%)
4	10.8 (5.0 to 17.5)	0.8 (−0.1 to 1.9)	4.3% (2.1% to 6.7%)	6.5% (−1.0% to 15.6%)
5 (most deprived)	9.2 (3.8 to 15.5)	0.9 (−0.2 to 2.0)	3.9% (1.6% to 6.0%)	7.2% (−2.1% to 15.6%)
Slope (crude)	−0.7 (95% CI −1.6 to 0.2)	−0.4 (95% CI −0.6 to −0.2)	−2.9% (95% CI −6.1% to 0.4%)	−1.6% (95% CI −2.8% to −0.3%)
Slope (directly age and sex-standardised)	4.7 (95% CI 3.8 to 5.7)	0.2 (95% CI 0.0 to 0.3)	−0.1% (95% CI −0.5% to 0.2%)	−1.5% (95% CI −2.7% to −0.2%)

Absolute and relative median reductions of cases prevented or postponed (CPP) are presented for cardiovascular disease (CVD) and gastric cancer (GCa).

The slope for absolute and relative reduction represents the absolute and relative equity slope index, respectively.

Brackets contain IQRs for the estimated CPP and 95% CIs for the slopes.

### Future options (2016–2030)

Under the ‘current policy’ scenario, IMPACT_NCD_ projected that median salt consumption would reduce further from 7.0 (IQR: 6.8–7.7) g/day in 2016 to 6.2 (IQR: 5.9–6.2) g/day in 2030. The addition of structural policies might reach the national target of 6 g/day by 2020. The less feasible ‘ideal’ policy scenario was estimated to reach 3.6 (IQR: 3.0–4.1) g/day by 2030. Inequality in salt consumption persisted under the ‘current policy’ projections and decreased moderately with the addition of structural policies.

Under the ‘current policy’ scenario, we calculated ∼1.4 million new cases of CVD (IQR: 1.3–1.4 million) and 530 000 deaths (IQR: 510 000−560 000). Similarly, for GCa we estimated some 80 000 new cases (IQR: 65 000−93 000) and 42 000 deaths (IQR: 35 000−49 000). Approximately 20 000 more cases of CVD and GCa can be prevented or postponed from the implementation of structural policies. Table 3 presents IMPACT_NCD_ estimates for the two counterfactual scenarios.

**Table 3:** Additional cases and deaths that can be potentially prevented or postponed (CPP, DPP) from the addition of structural policies to current policy, and under the ‘ideal scenario’

	Cardiovascular disease		Gastric cancer	
Scenario	CPP in thousands	DPP in thousands	CPP in thousands	DPP in thousands
Feasible	18.7 (8.0 to 29.5; Ps=90%)	3.6 (−0.4 to 8.1; Ps=72%)	1.2 (−0.2 to 3.0; Ps=72%)	0.7 (−0.9 to 2.3; Ps=63%)
Ideal	73.2 (53.9 to 94.3; Ps=100%)	11.0 (6.5 to 16.1; Ps=95%)	6.3 (3.4 to 9.6; Ps=94%)	3.1 (1.1 to 5.1; Ps=86%)

Compared with the current policy projections for 2015 to 2030.

Brackets contain the respective IQRs and the probability of superiority (Ps).

The addition of structural policies was more effective among the most deprived groups especially for CVD and might potentially decrease absolute socioeconomic inequality (table 4). As anticipated, the ‘ideal’ scenario had the largest impact on burden and inequality (table 5).

**Table 4:** Additional effectiveness of structural policies compared with the ‘current policy’ scenario by quantile group of Index of Multiple Deprivation (QIMD)

‘Feasible’ scenario	CPP absolute reduction in thousands		CPP relative percentage reduction	
QIMD	CVD	GCa	CVD	GCa
1 (least deprived)	2.7 (−1.0 to 6.4)	0.3 (−0.7 to 1.1)	1.6% (−0.5% to 3.6%)	2.6% (−6.2% to 10.3%)
2	2.4 (−1.2 to 6.6)	0.2 (−0.7 to 1.2)	1.3% (−0.7% to 3.6%)	2.4% (−6.6% to 10.4%)
3	2.8 (−1.0 to 6.8)	0.2 (−0.7 to 1.2)	1.5% (−0.7% to 3.6%)	2.4% (−7.0% to 10.2%)
4	2.8 (−1.3 to 7.0)	0.2 (−0.7 to 1.0)	1.6% (−0.7% to 3.9%)	2.2% (−7.5% to 11.2%)
5 (most deprived)	3.3 (−0.9 to 7.3)	0.3 (−0.7 to 1.2)	1.8% (−0.6% to 4.0%)	2.7% (−7.7% to 11.6%)
Slope	0.6 (95% CI 0.0 to 1.1)	0.0 (95% CI −0.1 to 0.2)	0.2% (95% CI −0.1% to 0.5%)	0.3% (95% CI −1.1% to 1.6%)
Slope (directly age and sex-standardised)	1.7 (95% CI 1.1 to 2.3)	0.1 (95% CI 0.0 to 0.2)	0.1% (95% CI −0.2% to 0.4%)	−0.2% (95% CI −1.6% to 1.1%)

Absolute and relative reductions of cases prevented or postponed (CPP) are presented for cardiovascular disease (CVD) and gastric cancer (GCa).

The slope for absolute and relative reduction represents the absolute and relative equity slope index, respectively.

Brackets contain IQRs for the estimated CPP and 95% CIs for the slopes.

**Table 5:** The additional effectiveness of ‘ideal’ compared with the ‘current policy’ scenario by quantile group of Index of Multiple Deprivation (QIMD)

‘Ideal’ scenario	CPP absolute reduction in thousands		CPP relative percentage reduction	
QIMD	CVD	GCa	CVD	GCa
1 (least deprived)	7.7 (3.3 to 12.6)	0.8 (−0.3 to 1.7)	4.2% (2.0% to 6.5%)	6.7% (−2.7% to 15.2%)
2	8.2 (3.6 to 12.6)	0.7 (−0.2 to 1.7)	4.1% (1.9% to 6.2%)	5.6% (−1.7% to 14.4%)
3	8.9 (4.0 to 14.4)	1.0 (−0.1 to 2.0)	4.4% (2.1% to 6.9%)	8.5% (−0.9% to 17.4%)
4	8.6 (3.5 to 13.3)	0.7 (−0.2 to 1.6)	4.4% (1.9% to 6.7%)	6.8% (−2.0% to 15.8%)
5 (most deprived)	9.7 (4.7 to 14.8)	1.0 (0.1 to 1.9)	4.9% (2.5% to 7.1%)	9.3% (1.0% to 18.4%)
Slope	2.1 (95% CI 1.4 to 2.8)	0.3 (95% CI 0.1 to 0.4)	0.8% (95% CI 0.5% to 1.2%)	3.4% (95% CI 2.0% to 4.7%)
Slope (directly age and sex-standardised)	5.7 (95% CI 5.0 to 6.3)	0.6 (95% CI 0.4 to 0.7)	0.7% (95% CI 0.3% to 1.0%)	2.9% (95% CI 1.5% to 4.3%)

Absolute and relative reductions of cases prevented or postponed (CPP) are presented for cardiovascular disease (CVD) and gastric cancer (GCa).

The slope for absolute and relative reduction represents the absolute and relative equity slope index, respectively.

Brackets contain IQRs for the estimated CPP and 95% CIs for the slopes.

### Validation

**Figure 2:**
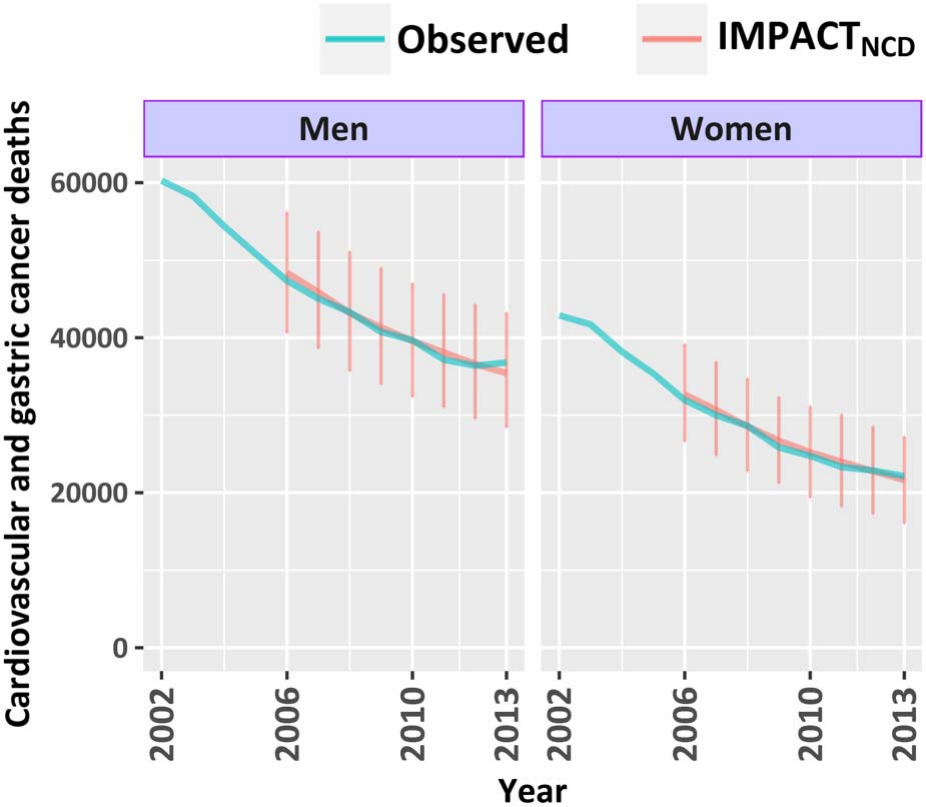
Number of deaths from cardiovascular disease and gastric cancer in England, by year and sex for ages 30–84. Office for National Statistics (ONS)-reported deaths (observed) versus IMPACT_NCD_-estimated. Observed deaths after 2010 were adjusted to account for changes in the ICD-10 version used by ONS since 2011.^36^ Error bars represent IQRs.

We assessed the eternal validity of the IMPACT_NCD_ model by comparing the estimated number of deaths from CVD and GCa against the observed number of deaths from the same causes for years 2006 to 2013 in England (figure 2). Detailed graphs by age group, sex, QIMD and disease can be found in the online supplement chapter S8. Overall, IMPACT_NCD_ is strongly validated even when mortality was highly stratified.

## Discussion

This is the first study to quantify the impact of UK salt reduction policies on CVD and GCa by socioeconomic group. We estimated that the current UK salt strategy has potentially prevented or postponed some 57 000 new cases and 12 000 deaths from CVD and GCa in England. The addition of structural policies and achievement on the national target by 2020 could potentially prevent or postpone a further 20 000 new cases and 4000 deaths, while the ‘ideal’ combination of salt reduction policies might potentially prevent or postpone some 80 000 new cases and 14 000 deaths from CVD and GCa.

When equity is considered, the impact of the implemented strategy is more complex. Our results agree with previous studies^13^ ^18^ that the socioeconomic gradient in salt consumption would not be reduced by these strategies. IMPACT_NCD_ estimated that current policies might have a rather neutral impact of CVD socioeconomic inequalities (absolute and relative) and worsen GCa inequalities reflecting an older age distribution in more affluent groups. However, the addition of structural policies may reduce absolute socioeconomic inequality in CVD incidence and neutralise the negative impact of current policies on GCa inequalities.

Simpler modelling studies have previously examined the impact of a theoretical decrease in UK salt consumption. A 3 g/day reduction in salt consumption might prevent about 32 000 CVD cases and 4500 CVD deaths in England and Wales in a 10-year period according to Barton *et al*,^37^ or 200 000 fewer CVD events and 90 000 fewer CVD deaths according to Dodhia *et al*^38^ or almost 100 000 fewer CVD deaths in 20 years according to Hedriksen *et al*.^39^ Our results appear to echo the more conservative estimates by Barton *et al*.^37^ In addition, Gillespie *et al*^20^ model that was informed by experts' opinion to model policy effectiveness and equity estimated that mandatory salt reformulation might reduce socioeconomic inequalities in CHD. We reached reassuringly similar conclusions using a very different methodology.

Going further than previous studies, we modelled structural interventions and as being more effective for those individuals with the highest salt intakes. In the UK, about 70% of dietary salt comes from processed food.^11^ Since structural policies target processed foods, their effect would be stronger among those with higher consumption of processed food, and hence higher salt intake.

Some researchers claim that salt consumption lower than 7.5 g can actually increase the risk of CVD and overall mortality.^40^ ^41^ However, it appears that their argument is based on biased measurement methodology. Previous studies that used the gold standard measure of individual salt intake, multiple non-consecutive 24-hour urine collections, to measure the salt exposure of their participants have consistently suggested that the optimal daily salt exposure is well below 6 g.^42^

### Public health implications

Our study confirms and quantifies the positive impact of the currently implemented UK salt reduction policies on CVD and GCa disease burdens. The overall health potential from salt reduction policies is likely to be greater, for example, through kidney disease, which we have not considered in our study. However, we also highlight two culprits of current policy. First, the national target of 6 g/day is unlikely to be reached in the next 15 years assuming the decline continues to be logarithmic. Second, the current policy will probably not reduce socioeconomic inequalities in CVD incidence and might even increase inequalities in GCa.

Structural policies, like a mandatory reformulation of processed foods, could potentially accelerate the decline in salt consumption and reduce absolute inequality in CVD. The existing salt reduction recommendations for the food industry could achieve the national target.^9^ In order to realise this, however, the food industry must comply with them, which is not happening at present.^43^ Failing to do so will most affect the poorest in society. Although we did not consider cost in our study, previous studies have suggested that mandatory reformulation is cost-effective and potentially cost-saving.^44^ ^45^

Many experts are supporting now the combined reformulation in portion sizes, sugar, salt and fat content of processed food with sanctions for food manufacturers that do not comply.^46^ After the derail of the salt reduction strategy in 2011 due to the ‘Responsibility Deal’, that transferred the responsibility for nutrition from the Food Standards Agency to the food industry itself, salt reduction efforts have been renewed since 2014.^7^ In fact, the second year of the Public Health England sugar reformulation programme is scheduled to also address salt in 2017.^47^

### Strengths and limitations

Our study uses a technically advanced microsimulation model that synthesises information from the best available sources of information on population exposures to salt, and other non-communicable disease-related risk factor, to generate a ‘close-to-reality’ synthetic population. Many assumptions must be made with such models. Yet, in spite of the potential frailty of such assumptions, this model validated well against observed CVD and GCa incidence and mortality in real populations, even when multiply stratified. This validation is particularly important because for the years after 2006 the incidence and mortality in the synthetic population were recreated from first epidemiological principles and not through an optimisation process. Moreover, to ensure transparency, we have made IMPACT_NCD_ source code open under GNU GPLv3 licence.

This study has many limitations, three of which are noteworthy. First, for the evaluation of current policy, we assumed that the decline in salt consumption observed since 2003 was fully attributable to the implemented policy. This was perhaps slightly simplistic and our estimates may, therefore, be high. Second, we did not model the effect of the ‘Responsibility Deal’ that potentially reduced the rate of salt decline since 2011.^7^ ^43^ However, this overestimation of the baseline would, therefore, reduce the apparent gains from additional structural policies, making our conclusions relatively conservative. Third, we could not find a sufficiently large data set with individual-level 24-hour urine sodium measurements and other non-communicable disease-related risk factor information. The stochastic process we developed to overcome this and synthesise information from multiple sources increased the overall uncertainty of the model. Nevertheless, this uncertainty has been quantified and transparently reported using uncertainty intervals.

## Conclusions

Current salt reduction policies are generally effective in reducing the cardiovascular and cancer disease burden but fail to do so equitably. Additional structural policies could achieve further, more equitable health benefits.
