# Supplementary material for: Estimated reductions in cardiovascular and gastric cancer disease burden through salt policies in England: an IMPACTNCD microsimulation study
Source: BMJ Open. 2017 Jan 24;7(1):e013791. doi: 10.1136/bmjopen-2016-013791 (PMC5278253; doi:10.1136/bmjopen-2016-013791)
Supplement: supplementary data [file bmjopen-2016-013791supp.pdf]

Supplementary Appendix

This appendix has been provided by the authors to give readers additional information about their work.

Supplement to: Estimated reductions in cardiovascular and gastric cancer disease burden through salt policies in England: an $IMPACT_{NCD}$ microsimulation study.

Table of Contents

CHAPTER S1. Summary of evidence about the risks of excess salt consumption 8

CHAPTER S2. High-level description of $IMPACT_{ncd}$ 9

S2.1.1. Technical information 9

CHAPTER S3. Population module 11

S3.1. Estimating exposure to risk factors (steps 2-3) 11

S3.2. Generating the ‘close to reality’ synthetic population for $IMPACT_{NCD}$ 11

S3.2.1. Stage 1: household structure 12

S3.2.2. Stage 2: socioeconomic variables 12

S3.2.3. Stage 3: behavioural variables 13

S3.2.4. Stage 4: biological variables 13

S3.3. $IMPACT_{NCD}$ implementation of individualised risk factor trajectories 13

S3.3.1. Age, sex and socioeconomic variables 14

S3.3.2. Salt 14

S3.3.3. Fruit & veg consumption and physical activity 16

S3.3.4. Smoking 16

S3.3.5. Environmental tobacco smoking 17

S3.3.6. Continuous biological variables 17

S3.3.7. Diabetes mellitus 18

S3.4. Lag times 19

S3.5. Birth engine (Step 4) 19

CHAPTER S4. Disease module 20

S4.1. Estimating the annual individualised disease risk and incidence (Step 5) 20

S4.1.1. Stage 1 20

S4.1.2. Stage 2 21

S4.1.3. Stage 3 21

S4.2. Simulating disease histories (Step 6) 22

S4.3. Simulating mortality (Step 7) 22

CHAPTER S5. Scenarios 24
CHAPTER S6. Uncertainty 26
CHAPTER S7. Equity metrics 28
S7.1. Absolute and relative equity slope index 28
CHAPTER S8. Validation 30
S8.1. Synthetic population validation 30
S8.2. Risk factor trends validation 41
S8.3. Incidence external validation 53
S8.4. Mortality external validation 54
Tables 62
References 74

List of Tables

TABLE S1 IMPACT$_{NCD}$ DATA SOURCES 62
TABLE S2 IMPACT$_{NCD}$ ASSUMPTIONS AND LIMITATIONS 68
TABLE S3 DISTRIBUTIONS THAT WERE USED AS INPUTS FOR THE SIMULATIONS. NUMBERS ARE ROUNDED 69

List of Figures

FIGURE S1 SIMPLIFIED IMPACT$_{NCD}$ ALGORITHM FOR INDIVIDUALS. FOR EACH STEP, THE ALGORITHM USES INFORMATION FROM ALL APPROPRIATE PREVIOUS STEPS. CHD DENOTES CORONARY HEART DISEASE. 10
FIGURE S2 PLOT OF THE CUMULATIVE DISTRIBUTION FUNCTION OF THE SELECTED DISTRIBUTION (LINE) AGAINST KNOWN QUANTILES (POINTS) FOR MEN, AGED 19 – 24 FROM SODIUM SURVEY 2001. 15
FIGURE S3 PLOT OF THE PERCENTILE RANK AGAINST THE SYSTOLIC BLOOD PRESSURE OF MALE SYNTHETIC INDIVIDUALS LIVING IN QIMD 3 AREA FOR AGE GROUPS 20-24 AND 70-74. 18
FIGURE S4 DENSITY PLOT OF SALT CONSUMPTION DISTRIBUTION FOR EACH SCENARIO OF THIS STUDY IN A SIMULATED YEAR. THE ALGORITHM DOES NOT ALLOW SALT CONSUMPTION < 1G/DAY 25
FIGURE S5 COMPARISON BETWEEN THE HEALTH SURVEY FOR ENGLAND 2006 (N = 17,633) AND A RANDOM SAMPLE (N=200,000) FROM THE SYNTHETIC POPULATION. DISTRIBUTION OF AGE GROUP, SEX AND QUINTILE GROUPS OF INDEX OF MULTIPLE DEPRIVATION (1=LEAST DEPRIVED, 5=MOST DEPRIVED) IS PRESENTED 31
FIGURE S6 COMPARISON BETWEEN THE HEALTH SURVEY FOR ENGLAND 2006 (N = 17,633) AND A RANDOM SAMPLE (N=200,000) FROM THE SYNTHETIC POPULATION. DISTRIBUTION OF AGE GROUP, SEX, QUINTILE GROUPS OF INDEX OF MULTIPLE DEPRIVATION (1=LEAST DEPRIVED, 5=MOST DEPRIVED) AND SMOKING STATUS IS PRESENTED 32

Figure S7 Comparison between the Health Survey for England 2006 (n = 17,633) and a random sample (n=200,000) from the synthetic population. Distribution of age group, sex, quintile groups of index of multiple deprivation (1=least deprived, 5=most deprived) and exposure to environmental tobacco is presented 33
Figure S8 Comparison between the Health Survey for England 2006 (n = 17,633) and a random sample (n=200,000) from the synthetic population. Distribution of age group, sex, quintile groups of index of multiple deprivation (1=least deprived, 5=most deprived) and portions of fruit and vegetable consumed per day is presented 34
Figure S9 Comparison between the Health Survey for England 2006 (n = 17,633) and a random sample (n=200,000) from the synthetic population. Distribution of age group, sex, quintile groups of index of multiple deprivation (1=least deprived, 5=most deprived) and exposure to days of more than 30 min of physical activity (PA) per week is presented. The small circles represent sub-groups with no participants. Their number reduced in the synthetic population sample highlighting the capability of the method to create individuals with traits not present in the original survey 35
Figure S10 Comparison between the Health Survey for England 2006 (n = 17,633) and a random sample (n=200,000) from the synthetic population. Distribution of age group, sex, quintile groups of index of multiple deprivation (1=least deprived, 5=most deprived) and diabetes mellitus is presented 36
Figure S11 Comparison of body mass index cumulative distributions in Health Survey for England 2006 (n = 17,633) and a random sample (n=200,000) from the synthetic population. Each panel depicts a different subgroup of the population based on quintile groups of index of multiple deprivation (QIMD, 1=least deprived, 5=most deprived), sex and age group 37
Figure S12 Comparison of systolic blood pressure cumulative distributions in Health Survey for England 2006 (n = 17,633) and a random sample (n=200,000) from the synthetic population. Each panel depicts a different subgroup of the population based on quintile groups of index of multiple deprivation (QIMD, 1=least deprived, 5=most deprived), sex and age group.. 38
Figure S13 Comparison of plasma total cholesterol cumulative distributions in Health Survey for England 2006 (n = 17,633) and a random sample (n=200,000) from the synthetic population. Each panel depicts a different subgroup of the population based on quintile groups of index of multiple deprivation (QIMD, 1=least deprived, 5=most deprived), sex and age group.. 39
Figure S14 Comparison of salt consumption cumulative distributions in Health Survey for England 2006 (n = 17,633) and a random sample (n=200,000) from the synthetic population. Each panel depicts a different subgroup of the population based on quintile groups of index of multiple deprivation (QIMD, 1=least deprived, 5=most deprived), sex and age group. Note that IMPACT$_{\mathbf{NCD}}$ applies another layer of processing to integrate information from 24h urine sodium measurements before risk estimation 40
Figure S15 Mean salt consumption for ages 19 – 64 between years 2001 and 2011. Observed in the population through surveys using 24h urine collections[39–42] vs. IMPACT$_{\mathbf{NCD}}$ synthetic population estimates. Error bars represent 95% confidence intervals of the mean. 41
Figure S16 Mean salt consumption by age group, between years 2001 and 2011. Observed in the population through surveys using 24h urine collections[39–42] vs. IMPACT$_{\mathbf{NCD}}$ synthetic population estimates. Error bars represent 95% confidence intervals of the mean. 42

Figure S17 Mean systolic blood pressure for ages 30 – 84 between years 2001 and 2012. Observed in the population through Health Survey for England vs. IMPACT$_{NCD}$ synthetic population estimates. Error bars represent 95% confidence intervals of the mean. 42
Figure S18 Mean systolic blood pressure for ages 30 – 84 by quintile group of index of multiple deprivation (QIMD, 1 = least deprived) between years 2001 and 2012. Observed in the population through Health Survey for England vs. IMPACT$_{NCD}$ synthetic population estimates. Error bars represent 95% confidence intervals of the mean. 43
Figure S19 Mean systolic blood pressure for ages 30 – 84 by age group, between years 2001 and 2012. Observed in the population through Health Survey for England vs. IMPACT$_{NCD}$ synthetic population estimates. Error bars represent 95% confidence intervals of the mean. 43
Figure S20 Mean total plasma cholesterol for ages 30 – 84 between years 2001 and 2012. Observed in the population through Health Survey for England vs. IMPACT$_{NCD}$ synthetic population estimates. Error bars represent 95% confidence intervals of the mean. 44
Figure S21 Mean total plasma cholesterol for ages 30 – 84 by quintile group of index of multiple deprivation (QIMD, 1 = least deprived) between years 2001 and 2012. Observed in the population through Health Survey for England vs. IMPACT$_{NCD}$ synthetic population estimates. Error bars represent 95% confidence intervals of the mean. 44
Figure S22 Mean total plasma cholesterol for ages 30 – 84 by age group, between years 2001 and 2012. Observed in the population through Health Survey for England vs. IMPACT$_{NCD}$ synthetic population estimates. Error bars represent 95% confidence intervals of the mean. 45
Figure S23 Mean body mass index for ages 30 – 84 between years 2001 and 2012. Observed in the population through Health Survey for England vs. IMPACT$_{NCD}$ synthetic population estimates. Error bars represent 95% confidence intervals of the mean. 45
Figure S24 Mean body mass index for ages 30 – 84 by quintile group of index of multiple deprivation (QIMD, 1 = least deprived) between years 2001 and 2012. Observed in the population through Health Survey for England vs. IMPACT$_{NCD}$ synthetic population estimates. Error bars represent 95% confidence intervals of the mean. 46
Figure S25 Mean body mass index for ages 30 – 84 by age group between years 2001 and 2012. Observed in the population through Health Survey for England vs. IMPACT$_{NCD}$ synthetic population estimates. Error bars represent 95% confidence intervals of the mean. 46
Figure S26 Smoking prevalence for ages 30 – 84 between years 2001 and 2012. Observed in the population through Health Survey for England vs. IMPACT$_{NCD}$ synthetic population estimates. Error bars represent 95% confidence intervals of the mean. 47
Figure S27 Smoking prevalence for ages 30 – 84 by quintile group of index of multiple deprivation (QIMD, 1 = least deprived) between years 2001 and 2012. Observed in the population through Health Survey for England vs. IMPACT$_{NCD}$ synthetic population estimates. Error bars represent 95% confidence intervals of the mean. 47
Figure S28 Smoking prevalence for ages 30 – 84 by age group between years 2001 and 2012. Observed in the population through Health Survey for England vs. IMPACT$_{NCD}$ synthetic population estimates. Error bars represent 95% confidence intervals of the mean. 48

Figure S29 Diabetes mellitus prevalence for ages 30 – 84 between years 2001 and 2012. Observed in the population through Health Survey for England vs. IMPACT$_{NCD}$ synthetic population estimates. Error bars represent 95% confidence intervals of the mean. 48
Figure S30 Diabetes mellitus prevalence for ages 30 – 84 by quintile group of index of multiple deprivation (QIMD, 1 = least deprived) between years 2001 and 2012. Observed in the population through Health Survey for England vs. IMPACT$_{NCD}$ synthetic population estimates. Error bars represent 95% confidence intervals of the mean. 49
Figure S31 Diabetes mellitus prevalence for ages 30 – 84 by age group between years 2001 and 2012. Observed in the population through Health Survey for England vs. IMPACT$_{NCD}$ synthetic population estimates. Error bars represent 95% confidence intervals of the mean. 49
Figure S32 Five or more portions of fruit & veg per day prevalence for ages 30 – 84 between years 2001 and 2012. Observed in the population through Health Survey for England vs. IMPACT$_{NCD}$ synthetic population estimates. Error bars represent 95% confidence intervals of the mean. 50
Figure S33 Five or more portions of fruit & veg per day prevalence for ages 30 – 84 by quintile group of index of multiple deprivation (QIMD, 1 = least deprived) between years 2001 and 2012. Observed in the population through Health Survey for England vs. IMPACT$_{NCD}$ synthetic population estimates. Error bars represent 95% confidence intervals of the mean. 50
Figure S34 Five or more portions of fruit & veg per day prevalence for ages 30 – 84 by age group between years 2001 and 2012. Observed in the population through Health Survey for England vs. IMPACT$_{NCD}$ synthetic population estimates. Error bars represent 95% confidence intervals of the mean. 51
Figure S35 Five or more active days per week prevalence for ages 30 – 84 between years 2001 and 2012. Observed in the population through Health Survey for England vs. IMPACT$_{NCD}$ synthetic population estimates. Error bars represent 95% confidence intervals of the mean. 51
Figure S36 Five or more active days per week prevalence for ages 30 – 84 by quintile group of index of multiple deprivation (QIMD, 1 = least deprived) between years 2001 and 2012. Observed in the population through Health Survey for England vs. IMPACT$_{NCD}$ synthetic population estimates. Error bars represent 95% confidence intervals of the mean. 52
Figure S37 Five or more active days per week prevalence for ages 30 – 84 by age group between years 2001 and 2012. Observed in the population through Health Survey for England vs. IMPACT$_{NCD}$ synthetic population estimates. Error bars represent 95% confidence intervals of the mean. 52
Figure S38 Gastric cancer cases in England for ages 30 – 84 by age group between years 2006 and 2012. Observed in the population through cancer registries vs. IMPACT$_{NCD}$ synthetic population estimates. Error bars represent 95% uncertainty intervals. 53
Figure S39 Number of deaths from coronary heart disease in England, by year and sex for ages 30 to 84. Office for National Statistics reported deaths (observed) vs IMPACT$_{NCD}$ estimated. 54
Figure S40 Number of deaths from stroke in England, by year and sex for ages 30 to 84. Office for National Statistics (ONS) reported deaths (observed) vs IMPACT$_{NCD}$ estimated. Observed deaths after 2010 were

adjusted to account for changes in ICD-10 version used by ONS since 201. Error bars represent interquartile ranges. 55
Figure S 41 Number of deaths from gastric cancer in England, by year and sex for ages 30 to 84. Office for National Statistics reported deaths (observed) vs IMPACT$_{NCD}$ estimated. 55
Figure S42 Coronary heart disease mortality (ICD10: I20 – I25) for men by age group and quintile group of index of multiple deprivation (QIMD, 1 = least deprived) between years 2002 and 2013. Observed in the population through mortality registries vs. IMPACT$_{NCD}$ synthetic population estimates. Whiskers represent 95% uncertainty intervals. 56
Figure S43 Coronary heart disease mortality (ICD10: I20 – I25) for women by age group and quintile group of index of multiple deprivation (QIMD, 1 = least deprived) between years 2002 and 2013. Observed in the population through mortality registries vs. IMPACT$_{NCD}$ synthetic population estimates. Whiskers represent 95% uncertainty intervals. 57
Figure S44 Stroke mortality (ICD10: I60 – I69) for men by age group and quintile group of index of multiple deprivation (QIMD, 1 = least deprived) between years 2002 and 2013. Observed in the population through mortality registries vs. IMPACT$_{NCD}$ synthetic population estimates. Whiskers represent 95% uncertainty intervals. 58
Figure S45 Stroke mortality (ICD10: I60 – I69) for women by age group and quintile group of index of multiple deprivation (QIMD, 1 = least deprived) between years 2002 and 2013. Observed in the population through mortality registries vs. IMPACT$_{NCD}$ synthetic population estimates. Whiskers represent 95% uncertainty intervals. 59
Figure S46 Gastric cancer mortality (ICD10: C16) for men by age group and quintile group of index of multiple deprivation (QIMD, 1 = least deprived) between years 2002 and 2013. Observed in the population through mortality registries vs. IMPACT$_{NCD}$ synthetic population estimates. Whiskers represent 95% uncertainty intervals. Uncertainty intervals could not be estimated for younger age groups due to small number of events. 60
Figure S47 Gastric cancer mortality (ICD10: C16) for women by age group and quintile group of index of multiple deprivation (QIMD, 1 = least deprived) between years 2002 and 2013. Observed in the population through mortality registries vs. IMPACT$_{NCD}$ synthetic population estimates. Whiskers represent 95% uncertainty intervals. Uncertainty intervals could not be estimated for younger age groups due to small number of events. 61

CHAPTER S1. SUMMARY OF EVIDENCE ABOUT THE RISKS OF EXCESS SALT CONSUMPTION

Excess dietary salt consumption has been linked to an increased risk of cardiovascular disease (CVD) and gastric cancer (GCa).[1–3] For CVD, the excess risk appears to be mainly mediated through the deleterious effect of excess salt consumption on blood pressure.[4,5] The pathophysiological mechanisms that link excess salt consumption with the increased risk for GCa are less clear. Some experimental studies showed increased inflammation of gastric mucosa, caused by high intragastric sodium concentrations, that leads to increased cell mutations. Other researchers suggest that a high salt diet facilitates gastric colonisation by Helicobacter pylori, a widely accepted risk factor for GCa, through changes in the viscosity of the gastric mucous barrier.[2,6,7]

There is some controversy regarding the optimal level of salt consumption.[8] The World Health Organisation (WHO) and the United Kingdom (UK) national guidelines recommend a daily salt intake of less than 5g and 6g, respectively.[9,10] Some researchers claim that salt consumption lower than 7.5g can actually increase the risk of CVD and overall mortality.[11,12] However, it appears that this argument is based on biased measurement methodology.[13] A recent discussion on the subject can be found in Mozaffarian et al who concluded that the optimal level of salt consumption below which no health gains have been observed is somewhere in the range of 1.5g to 6.0 g per day.[5 text S4] In our study we have incorporated the uncertainty around the ideal salt consumption in our probabilistic sensitivity analysis.

Evidence that directly links salt risk reversibility to CVD mortality or morbidity outcomes is lacking. A meta-analysis of several randomised control trials that tested low salt diets was underpowered and therefore inconclusive.[14] In comparison, a plethora exists on the effect of low salt diet on blood pressure which appears to happen within weeks.[4,5,15] Finally, to our knowledge there is no convincing evidence regarding risk reversibility for GCa.

The difference in risk reversibility lag times renders the implementation of experimental studies about salt risk reversibility on GCa impossible on ethical ground. For example, consider a randomised control trial to study the effect on GCa of an intervention that reduces salt consumption. Participants in the intervention arm of the study would have reduced mortality because of the favourable effect of reduced salt consumption on CVD. Because of the likely shorter lag time for CVD, this would have manifested earlier than any effect on GCa that has likely longer lag time and might well have resulted in early termination of the trial.[16]

CHAPTER S2. HIGH-LEVEL DESCRIPTION OF $IMPACT_{NCD}$

$IMPACT_{NCD}$ is a discrete time, dynamic, stochastic microsimulation model.[17,18] Within $IMPACT_{NCD}$ each unit is a synthetic individual and is represented by a record containing a unique identifier and a set of associated attributes.

For this study we considered age, sex, quintile groups of index of multiple deprivation (QIMD)[*], salt consumption, body mass index (BMI), systolic blood pressure (SBP), total plasma cholesterol (TC), diabetes mellitus (DM, binary variable)[†], smoking status (current/ex/never smoker), pack-years, environmental tobacco exposure (ETS, binary variable), fruit and vegetable (F&V) consumption and physical activity (PA) as the set of associated attributes. A set of stochastic rules is then applied to these individuals, such as the probability of developing coronary heart disease (CHD) or dying, as the simulation advances in discrete annual steps. The output is an estimate of the burden of CHD, stroke, and GCa in the synthetic population including both total aggregate change and, more importantly, the distributional nature of the change. This allows, among others, for an investigation of the impact of different scenarios on social equity.

$IMPACT_{NCD}$ is a complex model that simulates the life course of synthetic individuals and consists of two modules: The 'population' module and the 'disease' module. Figure S1 highlights the steps of the algorithm that generate the life course of each synthetic individual. We will fully describe $IMPACT_{NCD}$ by describing the processes in each of these steps in the following chapters. The description is from an epidemiological rather than technical perspective. The source code and all parameter input files are available in https://github.com/ChristK/IMPACTncd/tree/Evaluation_of_UK_salt_strategy under the GNU GPLv3 licence. Tables Table S1 and Table S2 summarise the sources of the input parameters and the main assumptions and limitations, respectively.

S2.1.1. Technical information

$IMPACT_{NCD}$ is being developed in R v3.2.0[20] and is currently deployed in an 80-core server with 2TB of RAM running Scientific Linux v6.2. $IMPACT_{NCD}$ is built around the R package 'data.table'[21], which imports a new heavily optimised data structure in R. Most functions that operate on a data table have been coded in C to improve performance. Each iteration for each scenario is running independently in one of the CPU cores and the R package 'foreach'[22] is responsible for the distribution of the jobs and collection of the results. To ensure statistical independence of the pseudo-random number generators

[*] QIMD is a measure of relative area deprivation based on the 2010 version of the Index of Multiple Deprivation[19]

[†] We defined as diabetics those with self-reported medically diagnosed diabetes (excluding pregnancy-only diabetes) or glycated haemoglobin (HbA1c) ≥ 6.5

running in parallel, the R package ‘doRNG’[23] was used to produce independent random streams of numbers, generated by L'Ecuyer's combined multiple-recursive generator.[24]

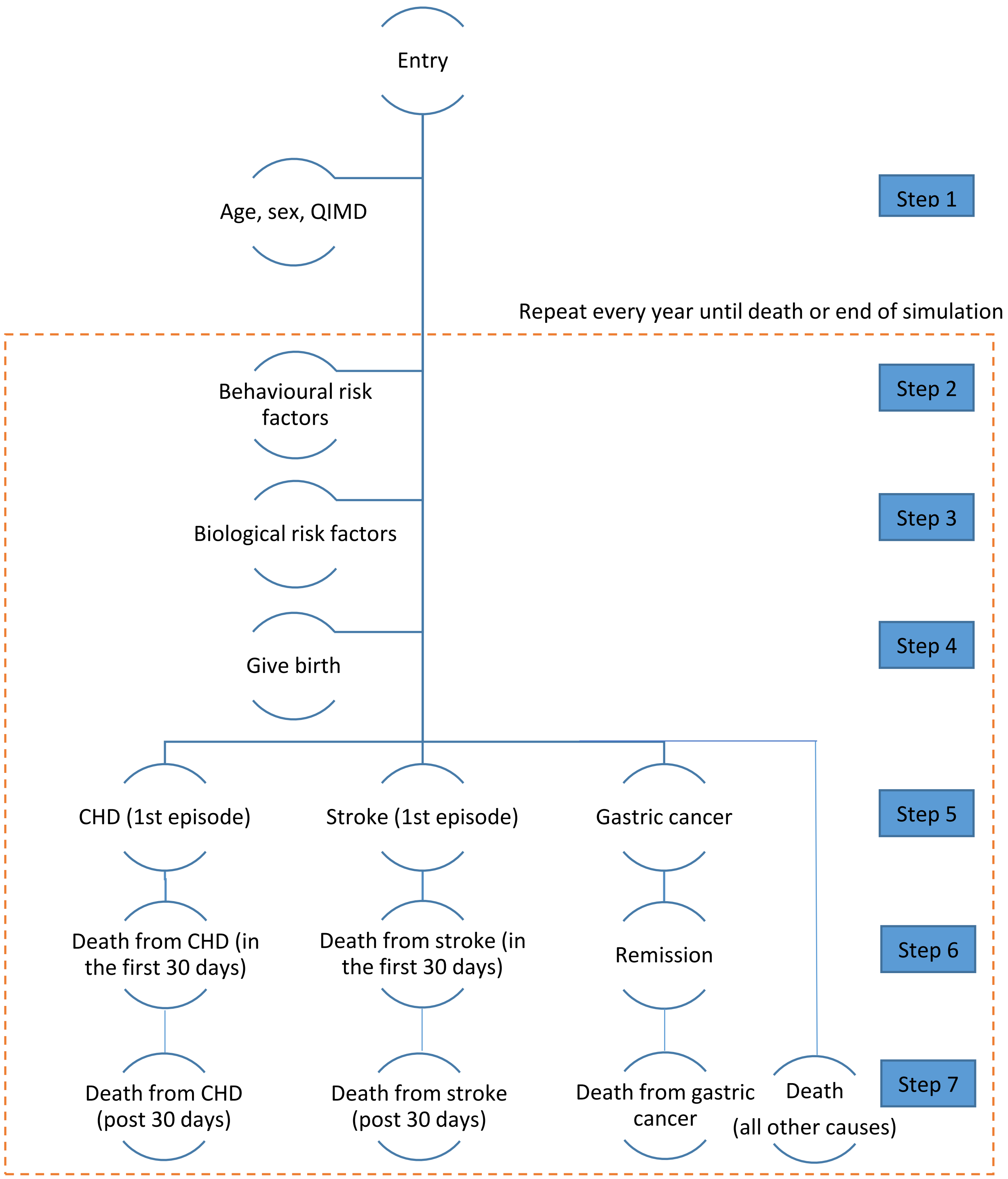

Figure S1 Simplified IMPACT$_{NCD}$ algorithm for individuals. For each step, the algorithm uses information from all appropriate previous steps. CHD denotes coronary heart disease.

CHAPTER S3. POPULATION MODULE

The 'population' module consists of steps 1 to 4 in Figure S1. Synthetic individuals enter into the simulation in the initial year (2006 for this study). The number of synthetic individuals that enter into the simulation is user defined and for this study was set to 200,000. The algorithm ensures that the age, sex and QIMD distribution of the sample is similar to this of the English population in mid-2006. This concludes step 1, which only happens at the beginning of each simulation. Following steps 2-7 are calculated annually (in simulation time) for each synthetic individual until the simulation horizon is reached, or death occurs.

S3.1. Estimating exposure to risk factors (steps 2-3)

In steps 2 and 3, $\text{IMPACT}_{\text{NCD}}$ estimates the exposure of the synthetic individual to the modelled risk factors. It is essential the risk profile of each synthetic individual to be similar to the risk profiles that can be observed in the real English population. For this, we first built a 'close to reality' synthetic population of England from which we sampled the synthetic individuals. Then, we used generalised linear models (GLM) for each modelled risk factor, to simulate individualised risk factor trajectories for all synthetic individuals.

S3.2. Generating the 'close to reality' synthetic population for $\text{IMPACT}_{\text{NCD}}$

The 'close to reality' synthetic population ensures that the sample of synthetic individuals for the simulation is drawn from a synthetic population similar to the real one in terms of age, sex, socioeconomic circumstance, and risk factors conditional distributions. In our implementation, we used the same statistical framework originally developed by Alfons et al[25] and adapted it to make it compatible with epidemiological principles and frameworks.

In general, this method uses a nationally representative survey of the real population to generate a 'close to reality' synthetic population. Therefore, the method expands the, often small, sample of the survey into a significantly larger synthetic population, while preserves the statistical properties and important correlations of the original survey.

The main advantages over other approaches are: 1) it takes into account the hierarchical structure of the sample design of the original survey, and 2) it can generate trait combinations which were not present in the original survey but are likely to exist in the real population. The second is particularly important because it avoids bias from the excessive repetition of combinations of traits present in the original survey that results from multilevel stratification of a relatively small sample. For example, the original survey may have two 35-year-old male participants, one with a BMI of 35 and the other with a BMI of 40 and no other 35-year-old male participants with BMI between 35 and 40. Unlike other methodologies, the approach proposed by Alfons et al can produce 35-year-old male synthetic

individuals with a BMI between 35 and 40. This is possible because the synthetic population is produced by drawing from conditional distributions that were estimated from multinomial models fitted in the original survey data. The detailed statistical methodology and justification can be found elsewhere.[25]

Our approach consists of four stages of which the first is common with the original method by Alfons et al.[25] The following stages have been adapted in order to be compatible with the widely accepted 'wider determinants of health' framework.[26] The main notion of this framework is that upstream factors such as the socioeconomic conditions, influence individual behavioural risk factors (e.g. diet, smoking), which in turn, influence individual downstream risk factors such as systolic blood pressure and total cholesterol. The four stages are:

1. Setup of the household structure.
2. Generate the socioeconomic variables.
3. Generate the behavioural variables.
4. Generate the biological variables.

In each stage, information from all previous stages is used. All the variables of the synthetic population for this study were informed by the Health Survey for England 2006 (HSE06).[27] The R language for statistical computing v3.2.0 and the R package 'simPopulation' v0.4.1 were used to implement the method.[20,28]

S3.2.1. Stage 1: household structure

The household size and the age and sex of the individuals in each household that have been recorded in HSE06 were used to inform the synthetic population, stratified by Strategic Health Authority (SHA)[*].

S3.2.2. Stage 2: socioeconomic variables

Once the basic age, sex, household and spatial information of the synthetic population was generated, other socioeconomic information was built up. QIMD for each synthetic individual was generated dependent on the household size and the age and sex of the individuals, stratified by SHA. Then, the equivalised income quintile groups[29] (EQV5) for each household was generated, dependent on five-year age groups and sex, stratified by QIMD. Finally, the employment status of the head of the household (HPNSSEC8) was generated using the National Statistics Socio-Economic Classification[30], dependent on 5-year age groups, sex and EQV5, stratified by QIMD.

[*] SHAs were 10 large geographic areas, part of the structure of the National Health Service in England before 2013. SHA is the only variable with spatial information in HSE06 and was used as a proxy, to roughly include some spatial information to the synthetic population.

S3.2.3. Stage 3: behavioural variables

In this stage, behavioural variables such as F&V portions per day, days achieving more than 30 min of moderate or vigorous PA per week, smoking status, exposure to ETS and salt consumption were generated, dependent on 5-year age groups, sex, HPNSSEC8 and EQV5, stratified by QIMD. Moreover, the use of statins and antihypertensive medication (two binary variables) was generated, dependent on 5-year age groups, sex and HPNSSEC8, stratified by QIMD. Other smoking related variables like cigarettes smoked per day for smokers, years since cessation for ex-smokers and pack-years for ever-smokers were also generated in this step. Specifically for salt consumption, HSE06 contains spot urine sodium measurements which are less reliable to 24h-urine sodium ones.[31,32] To overcome this limitation, IMPACT$_{NCD}$ adds another processing layer that is described separately (see paragraph S3.3.2 on page 14).

S3.2.4. Stage 4: biological variables

The last stage is the generation of the biological variables. Widely accepted causal pathways that have been observed in cohort studies, were used to identify associations between biological and behavioural variables. F&V consumption was used as a proxy to a healthy diet. Citations refer to specific evidence regarding the associations. BMI is associated with SBP[33–36], TC[37] and DM[38]. Thus, BMI was the first to be generated in the synthetic population dependent on 5-year age groups, sex, EQV5, F&V consumption[39] and PA[39–41], stratified by QIMD. Then, DM was generated dependent on 5-year age groups, sex, HPNSSEC8 and QIMD, stratified by BMI deciles. The TC was generated dependent on 5-year age groups, sex, deciles of BMI, use of a statin and F&V consumption, stratified by QIMD. Similarly, for the SBP the 5-year age groups, sex, deciles of BMI, smoking status[42,43] and deciles of salt consumption were used as predictors, stratified by QIMD. Socioeconomic variables were used as predictors for both behavioural and biological variables to allow for possible interaction between socioeconomic and behavioural variables.

The outcome of the method was to create a synthetic population of 55 million with similar characteristics to the non-institutionalised population of England in 2006. The synthetic population was validated against the original HSE06 sample (see p30, Synthetic population validation).

S3.3. IMPACT$_{NCD}$ implementation of individualised risk factor trajectories

IMPACT$_{NCD}$ only applies the previous process for the initial year of the simulation. As the simulation evolves over time, all variables are recalculated to take into account age and period effects. This feature justifies the classification of IMPACT$_{NCD}$ as a dynamic microsimulation. The process depends on the nature of each variable and the available information but generally, it uses HSE01 – HSE12[27,44–54] to capture the time trends by age, sex, and QIMD and project them into the future.

S3.3.1. Age, sex and socioeconomic variables

As the simulation progress in annual circles, the age of the synthetic individuals in the model increase by one year in each loop. The sex and socioeconomic variables remain stable. Therefore, social mobility is not simulated in the current version of $IMPACT_{NCD}$.

S3.3.2. Salt

For this study, we assume that all consumed salt is excreted through urine and all the sodium that is excreted in urine comes from the consumed salt. HSE06 measured sodium excretion from spot urine. We used the INTERSALT equation for Northern Europe to estimate daily sodium excretion from spot urine.[36] However, while this method is acceptable to estimate the mean sodium excretion of the population, it tends to overestimate low measurements and underestimate high measurements, when compared to the golden standard of sodium estimation from 24h urine collection.[31,32]

Additionally, sodium excretion from 24h urine collections was estimated in four nationally representative surveys times between 2001 and 2011 in the UK. [55–58] Unfortunately, the reported results are aggregated, stratified by age group and sex. Because individual-level data is not available from these surveys, their results cannot directly inform the synthetic population.

Hence, in order to synthesise the individual level information from the HSE with the less flexible but more accurate information from the sodium surveys we developed the following stochastic process:

Stage 1: The sodium surveys report several percentiles of the 24h urine sodium distribution by age group and sex. We used least squares estimation to fit known continuous univariate distributions.[*] The distribution with the best fit was selected and used for further calculation. The R package 'rriskDistributions' v2.1 was used for this.[59] The result of this stage was that for each age group, sex, and sodium survey year we estimated a known distribution for 24h urine sodium. For instance, a triangular distribution was selected for men, aged 19 – 24 in 2001 with parameters min ≈ 5.18, mode ≈ 7.3, and max ≈ 21.07 (Figure S2).

Stage 2: The four sodium surveys were performed in years 2001, 2006, 2008, and 2011. We used the nearest year HSE that individual level data for spot urine sodium was available and we converted the spot urine sodium to 24h sodium, using the INTERSALT equation for Northern Europe.[36] Instead of using fixed coefficients for the INTERSALT equation, for each HSE participant different coefficients were sampled from the normal distributions with mean equal to the coefficient and standard

[*] Normal, beta, Cauchy, logistic, *t*, chi-square, non-central chi square, exponential, *F*, gamma, lognormal, Weibull, triangular, PERT, truncated normal and Gompertz.

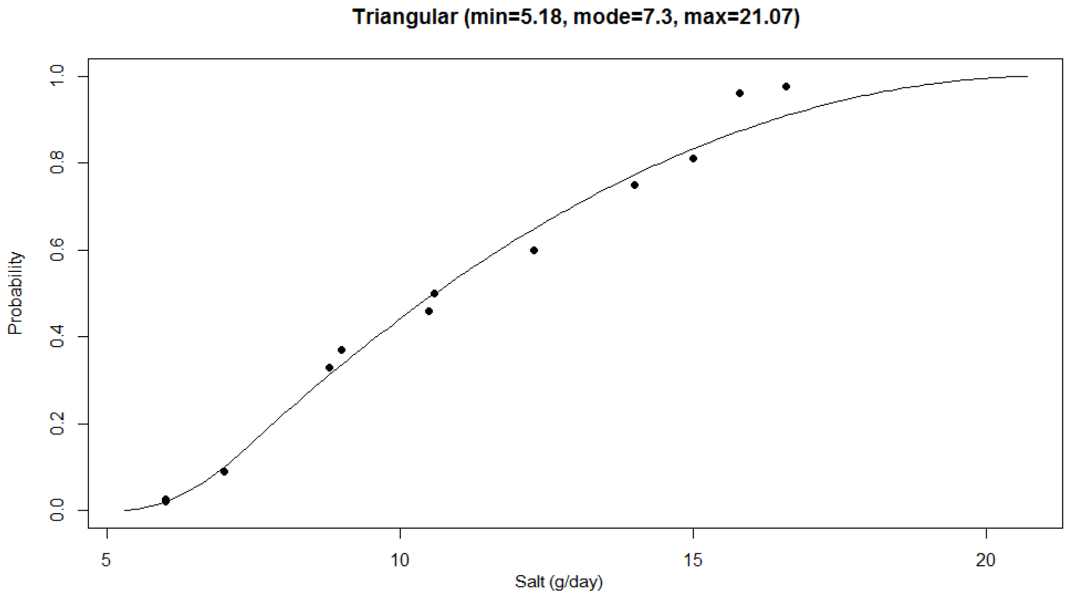

Figure S2 Plot of the cumulative distribution function of the selected distribution (line) against known quantiles (points) for men, aged 19 – 24 from sodium survey 2001.

deviation (sd) equal to the standard error (S.E.) of the respective coefficient. For instance, the reported INTERSALT age coefficient for men is 0.26 (S.E. = 0.78); therefore, for each use of the INTERSALT equation in this stage we draw a new age coefficient for men from a normal distribution with mean = 0.26 and sd = 0.78. Finally, 24h sodium (in mEq/day) is converted to salt (g/day) using the formula 1 mEq of sodium/day = 58.5 * 10^{-3} g of salt/day.

Stage 3: The rank of estimated salt for each HSE participant is calculated by age group, sex, and year. Then, the estimated salt consumption values from stage 2 are replaced by an equal number of values that were drawn from the respective (by age group, sex, and year) salt distribution that was estimated in stage 1, based on the equality[*] of ranks. For example, let us suppose a participant whose salt consumption was estimated in stage 2, at 10 g/day. Let us suppose that the percentile rank for his/her respective age group, sex and year corresponds to 0.6. Then in this step, a set of numbers[†] will be drawn from the respective distribution estimated in stage 1 and the value with a percentile rank of 0.6 will replace the 10 g/day salt consumption. Therefore, by the end of this stage, the individual level data from HSE03[322929], HSE06, HSE09[51], HSE12[54] regarding salt consumption, have very similar statistical properties as those reported in sodium surveys.

Stage 4: Quantile regression models are fitted to the series of HSE data with salt consumption as the dependent variable and ln(year of the survey - 1997), the 3rd degree of an orthogonal polynomial of

[*] Or maximum proximity if equality is not possible.
[†] With length equal to the number of participants in the respective age group, sex and year.

age, sex, QIMD and their 1st order interaction as the independent variables. The models are fitted for the 0.01, 0.05, 0.10, 0.15, …, 0.90, 0.95, 0.99 percentiles.

Stage 5: Stages 2 to 4 are repeated 500 times and 500 quantile regression models are built.

Stage 6: A quantile regression model is drawn from the models in stage 5 and is used to estimate the respective percentiles of the salt distribution by age, sex, QIMD and year. Then, the percentile rank* of salt consumption for each synthetic individual in $\text{IMPACT}_{\textbf{NCD}}$ is calculated from the previous year data. Based on their percentile rank, the minimum and maximum values for salt consumption is defined for each synthetic individual. For example, if the percentile rank of a synthetic individual is 0.23 the minimum and maximum values will be the 0.20 and 0.25 percentile respectively, as estimated from the quantile regression model for the respective age, sex, QIMD and year. Finally, a new salt consumption for the current year is drawn from the uniform distribution with the aforementioned minimum and maximum values.

The main advantage of this approach is that uses all the available information from the 24h urine sodium surveys, while enhances it with information regarding socioeconomic gradients and correlation with other risk factors and especially SBP, from spot urine measurements. The stochastic nature of the process allows its uncertainty to be estimated with Monte Carlo methods and is included in our reported uncertainty intervals.

S3.3.3. Fruit & veg consumption and physical activity

Both F&V consumption (portions/day) and PA (days with more than 30 min of moderate or vigorous activity/week) were modelled as ordinal factor variables. A proportional odds logistic regression model was fitted in the HSE01, HSE02, HSE04-11 individual level data with F&V consumption as the dependent variable and year, 2nd degree polynomial of age, sex, QIMD and their 1st order interactions. Similarly, for PA a similar model was fitted in the HSE06, HSE08 and HSE12 data. These models were used for individual-level predictions about the synthetic individuals as the simulation was evolving.

S3.3.4. Smoking

The 'close to reality' synthetic population is an accurate snapshot of active, ex-, and never-smokers in 2006, as it was observed in HSE06. Then $\text{IMPACT}_{\textbf{NCD}}$ uses transitional probabilities for smoking initiation, smoking cessation and relapse, to generate and record smoking histories of the synthetic individuals. For smoking initiation and cessation probabilities, logistic regression models were fitted

---

* For the percentile rank the formula $R_{percentile} = (R-1)/(n-1)$ is used, where $R_{percentile}$ is the percentile rank and $R = (R_1, \dots, R_n)$ is the rank vector constructed from a random observation vector $(X_1, \dots, X_n)$.

to HSE data with age, sex, and QIMD as the independent variables. A similar approach was followed for relapse probabilities with years since cessation, sex and QIMD as the independent variables.

S3.3.5. Environmental tobacco smoking

For ETS we assumed a linear relation between smoking prevalence and ETS, stratified by QIMD. We assumed no intercept; when smoking prevalence reaches 0, ETS prevalence will be 0 too.

S3.3.6. Continuous biological variables

In IMPACT$_{\mathbf{NCD}}$, the value of each continuous biological risk factor (BMI, SBP, and TC) is calculated in a two-stage process for each synthetic individual and each projected year. The first stage simulates ageing effects, while the second stage simulates period effects. We follow this approach mainly for two reasons. Firstly, to simulate physiological mechanisms of ageing. For example, the change of lipid profile in postmenopausal women, or the increase of SBP due to age-related stiffening of the arteries. Secondly, because the variance of the risk factor distributions increases with age, and we wanted to model this. Below we describe the stages:

Stage 1: Instead of tracking the actual biological risk factor values for the synthetic individuals, we track the percentile ranks[*] of the values by age, sex and QIMD. These percentile ranks remain fixed for each synthetic individuals throughout the simulation. In each simulated year, the percentile ranks are converted back to actual risk factor values, by matching the percentile ranks of a sample of the initial synthetic population of same age group, sex, and QIMD.

For example, in 2006 a 20-year-old male synthetic individual living in a QIMD 3 area with SBP of 120 mmHg has an SBP percentile rank of 0.52. Fifty years later, the same synthetic individual has retained his percentile score for SBP. However, his SBP is now calculated to 137.6 mmHg in order to match the SBP of a 70-year old man living in a QIMD 3 area in 2006 with the same percentile rank of 0.52. Figure S3 illustrates the previous example. Despite, individuals retain their percentile for the respective risk factor throughout the simulation (vertical position in Figure S3), this stage remains stochastic because each time this stage is implemented a different sample from the synthetic population is drawn. Finally, the distance from the mean for each risk factor is calculated stratified by 5-year age group, sex, and QIMD. For instance, if a synthetic individual has SBP of 140 mmHg and the mean SBP in the respective group of same age group, sex and QIMD is 130 mmHg, the distance from the mean is 140 – 130 = 10 mmHg.

---

[*] For the percentile rank the formula $R_{percentile} = (R-1)/(n-1)$ is used, where $R_{percentile}$ is the percentile rank and $R = (R_1, \dots, R_n)$ is the rank vector constructed from a random observation vector $(X_1, \dots, X_n)$. In IMPACT$_{\mathbf{NCD}}$ specifically, vector X is constructed from the subset of the respective continuous risk factor values, by 5 year age group, sex and QIMD, for each year of the simulation.

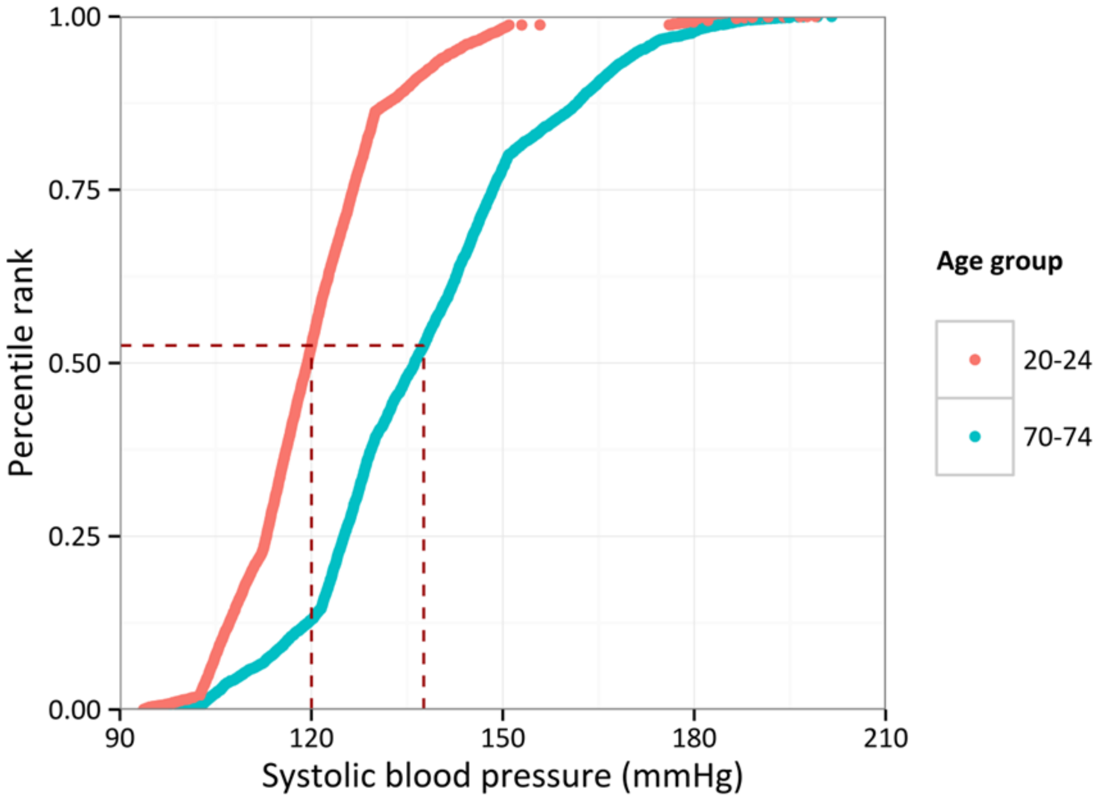

Figure S3 Plot of the percentile rank against the systolic blood pressure of male synthetic individuals living in QIMD 3 area for age groups 20-24 and 70-74.

Stage 2: Similarly to the approach followed for other variables, we fitted regression models to the HSE01-12 data. For BMI, year, age, sex, QIMD and PA were the independent variables. For SBP, year, age, sex, QIMD, smoking status, BMI, and PA were the independent variables. Finally for TC, year, age, sex, QIMD, BMI, F&V consumption and PA were the independent variables.* These models are used to predict the mean of the relevant group. These predicted means are added then, to the distances calculated in the previous stage. The result is the final value of the relevant risk factor that will be used for risk estimation.

S3.3.7. Diabetes mellitus

As with smoking, the 'close to reality' synthetic population is an accurate snapshot of diagnosed and non-diagnosed diabetics in 2006, as it was observed in HSE06. We assumed DM is an incurable chronic

* As before, the independent variables for each risk factor were selected based on known associations from longitudinal studies. Therefore, only the magnitude of the association is informed by cross-sectional data and possibly attenuated due to reverse causality.

condition. $IMPACT_{NCD}$ uses the validated for English population Qdiabetes algorithm (ex QDscore) to calculate annual transitional probabilities of non-diabetic synthetic individuals to develop DM.[60]

S3.4. Lag times

All the function that have been described above for risk factor trajectories include time and age (in years) as one of the independent variables. Therefore, lag times can be potentially considered on a per risk factor basis. For instance, let us consider a 50-year-old synthetic individual in 2010 and an assumed lag time of 5 years for F&V. When $IMPACT_{NCD}$ calculates the probabilities for F&V consumption of this individual, it will use time – (lag time) = 2010 – 5 = 2005 and age – (lag time) = 50 – 5 = 45. So, when the 'disease' module of $IMPACT_{NCD}$, uses the risk exposure to F&V to estimate a disease incidence transitional probability, the lag-timed exposure will be used.

In this study, we assumed that the mean lag time between exposure and CVD is 5 years.[61–63] Similarly, the mean lag time between exposure and GCa is 8 years, except for the cumulative risk of smoking (smoking duration) which was set to follow CVD lag time. Mean lag times were roughly informed from risk reversibility trials, when available, or the median observation times of the cohort studies we used to inform the risk magnitude for each risk factor. Then for each iteration, we draw lag time values from binomial distributions with the respective means.

S3.5. Birth engine (Step 4)

The Office for National Statistics (ONS) principal-assumption fertility projections for England are used to estimate the number of new synthetic individuals entering the model through birth, in every simulated year.[64] The birth engine only becomes relevant for simulations featuring a horizon of more than 30 years and its importance increases as the simulation progress further in time. The 'new-born' synthetic individuals inherit the socioeconomic position of their mother and their quantile ranks for the continuous biological risk factors from a random synthetic individual.

CHAPTER S4. DISEASE MODULE

The disease module contains the last 3 steps of the model (Figure S1). The risk (probability) for each synthetic individual aged 30 – 84, to develop each of the modelled diseases is estimated in step 5 conditional on the exposure to relevant risk factors. The step ends by selecting synthetic individuals to develop the modelled diseases. Finally, in steps 6 and 7 the risk of dying from one of the modelled diseases or any other cause is estimated and applied. Steps 2 to 7 are then repeated for the surviving individuals until the simulation horizon is reached.

S4.1. Estimating the annual individualised disease risk and incidence (Step 5)

In order to estimate the individualised annual probability of a synthetic individual to develop a specific disease conditional on his/her relevant risk exposures we follow a 3-stage approach:

1. The proportion of incidence attributable to each modelled risk factor by age group and sex is estimated, assuming a specific time lag.
2. Assuming multiplicative risks, the portion of the disease incidence attributable to all the modelled risk factors is estimated and subtracted from the total incidence.
3. For each individual in the synthetic population, the probability of developing the disease is estimated and then is used in an independent Bernoulli trial to select those who finally develop the disease.

Next, the implementation of the above method is described in more detail using CHD as an example. The same process is used for all modelled diseases.

S4.1.1. Stage 1

The population attributable risk (PAF) is an epidemiological measure that estimates the proportion of the disease attributable to an associated risk factor.[65] It depends on the relative risk associated with the risk factor and the prevalence of the risk factor in the population. In a microsimulation context where exposure to risk factors are known to individual level and assuming multiplicative risk factors PAF can be calculated with the formula:

$$PAF = 1 - \frac{n}{\sum_{i=1}^{n}(RR_1 * RR_2 * \dots * RR_k)} ,$$

where n is the number of synthetic individuals in the population, and $RR_{1\dots k}$ is the relative risks of the risk factors associated with CHD. We calculated PAF based on above formula stratified by age and sex. Consistent with findings from the respective meta-analyses that were used for $IMPACT_{NCD}$ (Table S1), SBP below 115 mmHg, TC below 3.8 mmol/l and BMI below 20 Kg/m^2 were considered to have a relative risk of 1. Similarly, consumption of eight or more portions of F&V and five or more days with

more than 30 minutes of moderate to vigorous activity per week were also considered to have a relative risk of 1. All the relative risks were taken from published meta-analyses and cohort studies (Table S1).

S4.1.2. Stage 2

The incidence of CHD not attributable to the modelled risk factors can be estimated by the formula:

$$I_{Theoretical\ minimum} = I_{Observed} * (1 - PAF)$$

Where $I_{Observed}$ is the CHD incidence and PAF is from Step 1. $I_{Theoretical\ minimum}$ represents CHD incidence if all the modelled risk factors were at optimal levels. The theoretical minimum incidence is calculated by age and sex only in the initial year of the simulation and it is assumed stable thereafter.

S4.1.3. Stage 3

Assuming that $I_{Theoretical\ minimum}$ is the baseline annual probability of a synthetic individual to develop CHD for a given age and sex due to risk factors not included in the model (i.e. genetics etc.), the individualised annual probability to develop CHD, $\mathbb{P}(\mathrm{CHD} \mid \mathrm{age, sex, exposures})$, given his/her risk factors were estimated by the formula:

$$\mathbb{P}(CHD \mid age, sex, exposures) = I_{Theoretical\ minimum} * RR_1 * RR_2 * RR_3 * \dots * RR_k$$

Where $RR_{1 \dots k}$ the relative risks that are related to the specific risk exposures of the synthetic individual, same as in stage 1. Depending on data availability this method can be further stratified by QIMD; however, data were not available for this in the current study.

The above method can be used only when the incidence of the disease in the population is known. For cancers, this information is available from the cancer registries. The true incidence of CHD (and stroke) though, is largely unknown. Several estimates exist nonetheless all have limitations. Therefore, for the estimation of CHD incidence by age and sex we opted for a modelling solution to synthesise all the available sources of information and minimise bias. Specifically, we used ONS CHD mortality (ICD10 I20-I25) for England in 2006,[66] self-reported prevalence of CHD from HSE06, the incidence of angina from primary care data[67] and incidence of acute myocardial infarction (AMI) from mortality and hospital statistics[68] to inform the WHO DISMOD II model.[69] DISMOD II is a multi-state life table model that is able to estimate the incidence, prevalence, mortality, fatality and remission of a disease when information about at least three of these indicators is available. A similar approach has been followed by the Global Burden of Disease team and others.[70,71] We considered CHD an incurable chronic disease (i.e. remission rate was set to 0); therefore, the derived DISMOD II incidence refers to the first ever manifestation of angina or AMI excluding any recurrent episodes. For the DISMOD II calculations, we assumed that incidence and case-fatality had been declining by 3% (relative), over

the last 20 years. The derived CHD incidence, prevalence and fatality were used as an input for $IMPACT_{NCD}$. A similar approach was used for stroke.

For the initial year of the simulation, some synthetic individuals need to be allocated as prevalent cases for each of the modelled diseases. DISMOD II model[69] is used again to estimate the number of prevalent cases of the disease by age and sex. Then, the estimated number of prevalent cases are sampled independently from the individuals in the population with weights proportional to their relevant exposures.

S4.2. Simulating disease histories (Step 6)

In the current stage of development, $IMPACT_{NCD}$ does not contain a detailed disease history module. However, Step 6 is used to simulate significant aspects of the disease. For CVD, this was used to simulate the observable spike of short-term (30 days) mortality after the first event of AMI or stroke. Data about short-term mortality were used from the 'Coronary heart disease statistics 2012 edition' report.[67]

For GCA this step is used to simulate remission cases. Once more, we used the DISMOD II model to estimate the remission rate by age and sex, using as inputs incidence, mortality, and case fatality rates by age group and sex. Specifically, the incidence and survival rates of GCa is known through the cancer registries and is reported by ONS.[72,73] From the reported first and fifth-year survival rate, assuming a Weibull survival distribution, we calculated annual case fatality and 10-year survival rate. Finally, we used the observed GCa mortality reported by ONS.[66] We assumed remission rate equals the 10-year survival rate. Furthermore, we assumed the incidence and case-fatality rate had been declining by 2% (relative) over the last 20 years and the remission rate had been improving by 1% (relative).

S4.3. Simulating mortality (Step 7)

All synthetic individuals are exposed to the risk of dying from any of their acquired modelled diseases or any other non-modelled cause. However, the algorithm behaves differently depending on the age and life course trajectory of the synthetic individual.

For ages 0 to 29 we used all-cause mortality rate by age, sex, and QIMD to inform an independent Bernoulli trial and select synthetic individuals that die every year. For years 2006 to 2013 we used the observed mortality rates as were reported from ONS.[66] For years after 2013, functional demographic models by sex and QIMD were fitted to the ONS reported annual mortality rates, from years 2002 to 2013, and then were projected to the simulation horizon using the R package 'demography'.[74] Functional demographic models are generalisations of the Lee-Carter demographic model, influenced by ideas from functional data analysis and non-parametric smoothing.[75]

The same approach as above was followed for synthetic individuals aged 85 to 100. We considered a mortality rate of 1 for all synthetic individuals reaching the age of 100. Hence, $IMPACT_{NCD}$ maximum synthetic individual age is 100 years.

Finally, for synthetic individuals with ages between 30 and 84 the all-cause mortality was decomposed into modelled-diseases specific mortality and any-other-cause mortality. The former applies only to the prevalent cases of each modelled disease in the synthetic population. For this, case-fatality rates by age and sex are estimated by DISMOD II for each modelled disease, as described before, and then are used in a Bernoulli trial to select prevalent cases that die from the disease in a year.

For the any-other-cause mortality, a process similar to the one described for ages 0 to 29 and 85 to 100. However, this time CVD and GCa specific mortality are removed from the observed mortality and mortality projections to avoid double counting.

The case mortality and fatality rates are further parametrized and individualised based on established epidemiological evidence. The ‘male British doctors’ and DECODE studies have shown that smokers and diabetics have increased overall mortality even when CVD is excluded[76,77]. $IMPACT_{NCD}$ adjusts for that by inflating the any-other-cause mortality rate for smokers and diabetics and deflating it for non-smokers and non-diabetics, while it constrains the sum to remain the same as before the adjustments. Furthermore, we assumed that CVD and GCa case-fatality is improving by 3% and 2% annually, respectively and that there is a constant case-fatality socioeconomic gradient of approximately 5% by QIMD level (halved for ages over 70) for CHD and GCa, and 2% for stroke. The socioeconomic gradient forces the more deprived to experience worse disease outcomes. These assumptions are based on empirical evidence.[67,78–80]

Finally, synthetic individuals who remain alive after this step progress to the next year and start again from step 1, unless the simulation horizon has been reached.

CHAPTER S5. SCENARIOS

The method described above is used to for the 'Current Policy' scenario. In general, primary prevention interventions or policies can then be modelled as counterfactual scenarios, through their effects on the relevant risk factors, mainly in three ways:

1. Population-wide interventions can be modelled, by altering the intercept or the coefficients of the regression equations that are used to estimate risk factor exposures. For example, when continuous risk factors are considered, adding or subtracting from the intercept increases or decreases the related risk factor for each synthetic individual; therefore, the mean of the risk factor for the whole population. Altering the year coefficient accelerates, decelerates or reverses the trend for the whole population. Likewise, altering the QIMD coefficients or/and the coefficient of the interaction between year and QIMD can simulate differential effects and trends by QIMD. A similar approach sometimes can be used also for the non-continuous risk factors. The benefit is that by just altering a few parameters the changes are translated down to individual level characteristics in a computationally efficient way.
2. Targeted interventions can be modelled by selecting synthetic individuals with a specific trait or combination of traits, and apply an intervention to them. For example, to simulate the effect of statins a simple approach would be to randomly select 30% of the synthetic individuals with TC higher than 4 mmol/l not currently on statins; and apply a 25% reduction of their TC between steps 4 and 5 (Figure S1).
3. Some hybrid combinations of the previous methods or some more complex approaches have the time slow down, stop in a specific year, or running backwards to simulate `disaster' scenarios.

Specifically for this study, the 'No intervention' scenario was modelled by stopping the time in 2003 for the quantile regression equation that predicts salt consumption. For the impact on SBP, salt reduction was estimated by rerunning the same equation for the appropriate year and calculate the difference for each synthetic individual using the formula from Mozaffarian et al.[5]

The 'Feasible' and 'Ideal' scenarios were modelled by allowing the 'Current Policy' to progress. Then after Step 4 (Figure S1), the mean salt consumption in the population aged 20 – 64[*] was calculated. From the year the intervention was applied (2015), if the mean was higher than the target then salt consumption of every synthetic individual was multiplied by the target divided by the mean of the synthetic population. Therefore, we applied a proportional reduction to all synthetic individuals and

[*] Previous 24h urine sodium surveys were conducted for the age group 19 – 64. We assumed that salt monitoring will continue to assess salt consumption in the same age group.

those with higher salt consumption had the higher reduction, in order synthetic population mean for ages 20 – 64 to reach the target. The impact of salt reduction on SBP was calculated as in the 'No intervention' scenario. Figure S4 shows the density plots of salt consumption for the scenarios of this study, in one iteration of the simulation.

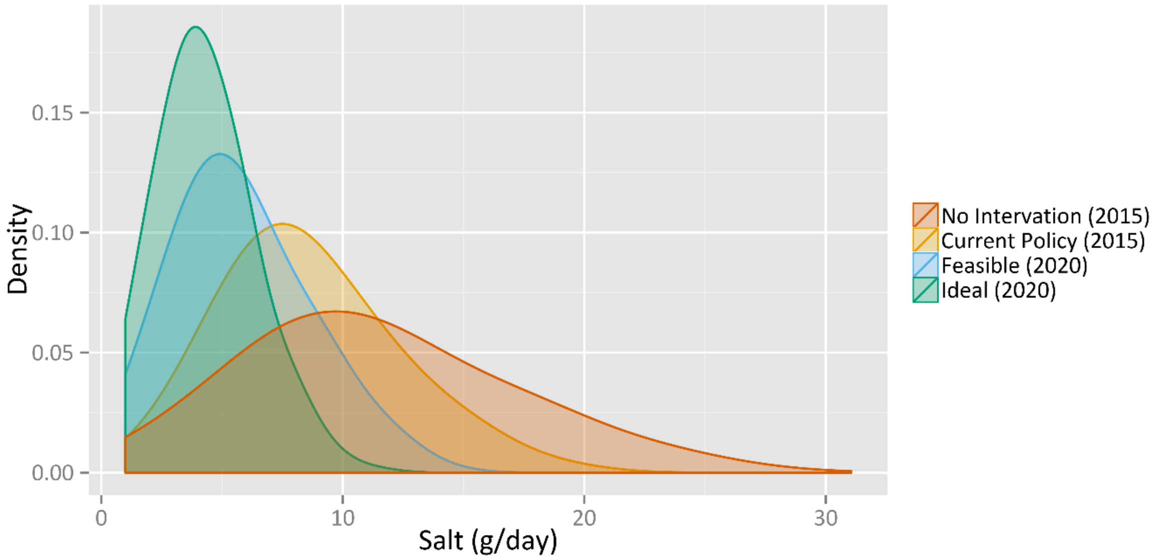

Figure S4 Density plot of salt consumption distribution for each scenario of this study in a simulated year. The algorithm does not allow salt consumption < 1g/day

CHAPTER S6. UNCERTAINTY

IMPACT$_{NCD}$ implements a 2nd order Monte Carlo approach to estimate uncertainty intervals (UI) for each scenario.[81,82] Each simulation runs 1000 times. For each iteration, a different set of input parameters is used, by sampling from the respective distributions[*] of input parameters (Table S3), and a different sample of the synthetic population is drawn. However, the scenarios are 'paired'. For instance, the *n*th iteration of all scenarios runs with the same set of input parameters and on the same synthetic population sample for all of them.[†] This explains why the uncertainty of in-between scenarios comparisons is significantly smaller than the uncertainty of isolated scenarios.

The framework allows stochastic uncertainty, parameter uncertainty and individual heterogeneity to be reflected in the reported UI. The following example illustrates the different types of uncertainty that were considered in IMPACT$_{NCD}$. Let us assume that the annual risk for CHD is 5%. If we apply this risk to all individuals and randomly draw from a Bernoulli distribution with p = 5% to select those who will manifest CHD, we only consider stochastic uncertainty. If we allow the annual risk for CHD to be conditional on individual characteristics (i.e. age, sex, exposure to risk factors), then individual heterogeneity is considered. Finally, when the uncertainty of the relative risks due to sampling errors is considered in the estimation of the annual risk for CHD, the parameter uncertainty is considered. From these three types of uncertainty, only the parameter uncertainty can be reduced from better studies in the future.

Due to lack of information and for computational efficiency, not all three types of uncertainty are considered in every step (Figure S1) of IMPACT$_{NCD}$. Specifically, stochastic uncertainty is included in every step, individual heterogeneity in every step except 1 and 4 and parameter uncertainty in step 5. Of course, parameter uncertainty (if any) of scenario targets are also estimated in steps 2 and 3. For example, the target of the 'Feasible' scenario is mean salt consumption of 6g/day and its uncertainty assumed to follow a PERT distribution with min = 5.8 g/day, mode = 6 g/day, and max = 7 g/day

The structure of the model is grounded on fundamental epidemiological ideas and well-established causal pathways; therefore, we considered this type of uncertainty relatively small and did not study it. However, mortality from each of the modelled diseases and any-other-cause (steps 6 and 7) is

---

[*] We assumed log-normal distributions for relative risks and hazard ratios, normal distributions for coefficients of regression equations, and PERT distributions for other parameters. Specifically for relative risks and hazard ratios, the distributions were bounded above 1 when the mean was above 1 and vice versa.

[†] Individual life-course trajectories however, are not. The same normotensive individual may evolve and develop hypertension under scenario 'A' but not under scenario 'B' due to chance, and not as a direct effect of the scenarios.

calculated serially, one modelled disease at a time. To avoid bias that this approach might introduce, the order of the modelled diseases in each mortality estimation is randomised.

From our experience in communicating our results to policy makers and researchers, we realised that they tend to misinterpret 95% UIs as 95% confidence intervals (CI) and overlapping UIs as 'evidence against statistical significance'. This does not apply in our model because the scenarios share common sources of uncertainty as explained above; therefore, scenarios are not independent. We decided to present medians and interquartile ranges (IQRs) exactly to avoid this misunderstanding with UIs and CIs. We hope that readers will mentally visualise the distribution from medians and IQRs rather than attempt to apply frequentist statistical inference and hypothesis testing rules, which do not apply in this particular situation. In any case, all our output distributions were approximately normal and their standard deviation can be approximated by dividing IQR with 1.35. Then, z-scores can be used to approximate any probability of UI.

CHAPTER S7. EQUITY METRICS

S7.1. Absolute and relative equity slope index

The ‘absolute equity slope index’ and the ‘relative equity slope index’ are two regression-based metrics, to measure the impact of the modelled interventions on absolute and relative socioeconomic health inequalities. They are inspired by the slope index of inequality (SII) and the relative index of inequality (RII);[83] however, instead of directly measuring inequalities in a population, like SII and RII do, they measure the impact of an intervention to existing inequalities.

The basic principles of the metrics are illustrated in this simplified example. Let us consider the simple example of a population that consists of only two mutually exclusive and same-sized socioeconomic groups, the ‘deprived’ and the ‘affluent’. The two groups experience different incidence of a disease; supposedly, 50 and 10 incident cases among the deprived and the affluent, respectively, every year. Hence, the absolute socioeconomic inequality for disease incidence is 50 – 10 = 40 cases and the relative socioeconomic inequality is 50 / 10 = 5. If a hypothetical intervention ‘A’ prevents the same number of cases in both groups, absolute inequality will remain stable. Similarly, if intervention ‘A’ prevents more cases in the affluent group, absolute inequality will increase and vice versa. For relative inequality to remain stable, the decrease in cases needs to be proportional to the observed number of cases. For example, a hypothetical intervention ‘B’ that reduces 10% of cases in each group will have no effect on relative inequality. If the proportional reduction is higher in the affluent group compared to the deprived, then relative inequality will increase and vice versa.

As in many real-world examples, $\text{IMPACT}_{\textbf{NCD}}$ uses QIMD to classify population in five socioeconomic groups of unequal sizes. In this case, SII and RII can be used to measure absolute and relative socioeconomic inequalities in health, respectively. The same principles of intervention effectiveness and inequalities described in the previous paragraph, also apply here. If an intervention prevents an equal number of cases in all QIMD groups SII will remain unchanged, while if the proportional reductions of cases in all QIMD groups are equal, RII will remain unchanged.[*] Inspired by SII and RII, the absolute equity slope index is the slope of the regression line fitted in the number of cases prevented or postponed by an intervention (dependent variable), on ridit scores[84] of QIMD (independent variable). Ridit scores reflect the average cumulative frequency of each QIMD group.[†] As in SII and RII they are used to account for the different sizes of each QIMD group (the distribution

---

[*] Assuming that the deaths prevented by the intervention does not change the relative size of the socioeconomic groups.

[†] So, if in QIMD 1,2,3,4 and 5 areas live 14%, 22%, 22%, 24% and 18% of the population respectively, the cumulative frequency is 14%, 36%, 58%, 82% and 100% and the rigid scores are 0+0.14/2 = 0.07, (0.14+0.36)/2 = 0.25, (0.36+0.58)/2 = 0.45, (0.58+0.82)/2 = 0.7 and (0.82+1)/2 = 0.91

of inequality) and allow for comparisons between populations. A positive slope means that the intervention prevents more cases in the more deprived QIMD groups and reduces absolute inequality in the population, and vice versa. The magnitude of the slope is proportional to the reduction in absolute inequality. The relative equity slope index is constructed and interpreted similarly, except that the proportion of cases prevented or postponed over the total cases in each socioeconomic group is the independent variable, and it measures the effect on relative inequality.

CHAPTER S8. VALIDATION

For this study, $IMPACT_{NCD}$ is calibrated to data from 2006 or before. The only exception is the regression models that are used in steps 2 and 3 (Figure S1) for individual predictions of exposure to risk factors. These models were fitted in data from 2001 to 2012. In this chapter, we first present the internal validation of the synthetic population and the risk factor trends, as an evidence that the synthetic population used in $IMPACT_{NCD}$ was similar to English population. Then, we present the external validation of $IMPACT_{NCD}$ by comparing observed to estimated mortality rates for years 2006 to 2013 by age group, sex, QIMD, and modelled disease. Specifically for GCa, we also compare observed and estimated incidence rates for the same time period by age group and sex.[*]

S8.1. Synthetic population validation

The following graphs compare a random sample of 200,000 synthetic individuals from the synthetic population to the original sample of HSE06 (n = 17,633). Mosaic plots[†] were used for the categorical variables and cumulative distribution plots were used for the continuous variables. Specifically in this document, the area of each tile of the mosaic plots is proportional to the proportion of each subgroup in the respective population. Only graphs that were relevant to the analysis for this study are presented here.

The graphs support the argument that the final synthetic population is close to reality, at least as it was captured through the HSE06, and are useful for the internal validation of the method. Alfons et al. used a statistical simulation approach to evaluate the process and showed that this method produces synthetic populations very similar to the original survey.[25] Of course, the method cannot overcome any limitations of the original survey, such as selection bias, or misclassification.

---

[*] For CHD and stroke, true incidence rates are largely unknown, so this part of the model cannot be easily validated.

[†] Mosaic plots are graphical representations of a contingency table of two or more categorical variables, using tiles with areas proportional to the frequencies in each cell of the table.[85]

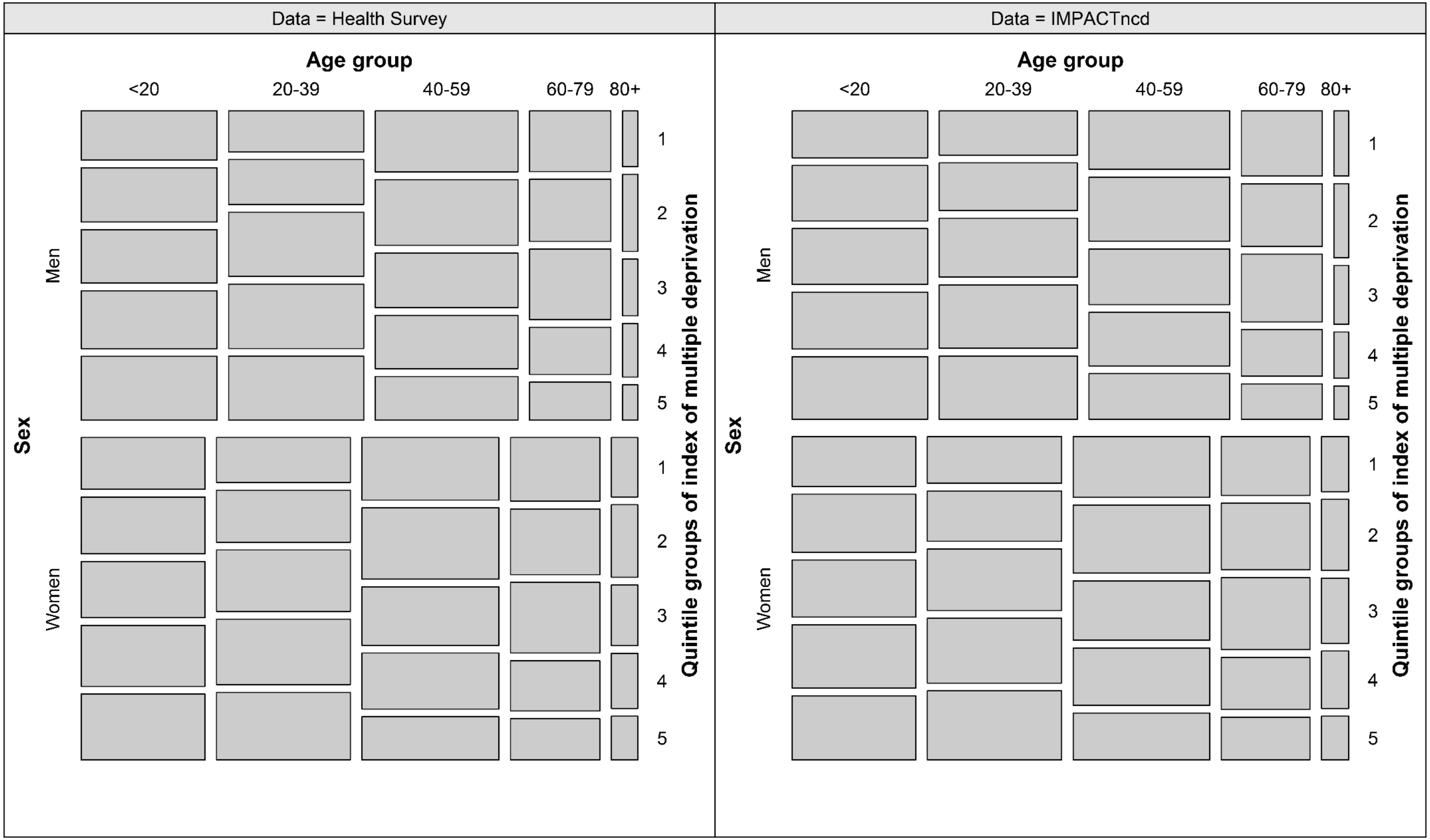

Figure S5 Comparison between the Health Survey for England 2006 (n = 17,633) and a random sample (n=200,000) from the synthetic population. Distribution of age group, sex and quintile groups of index of multiple deprivation (1=least deprived, 5=most deprived) is presented

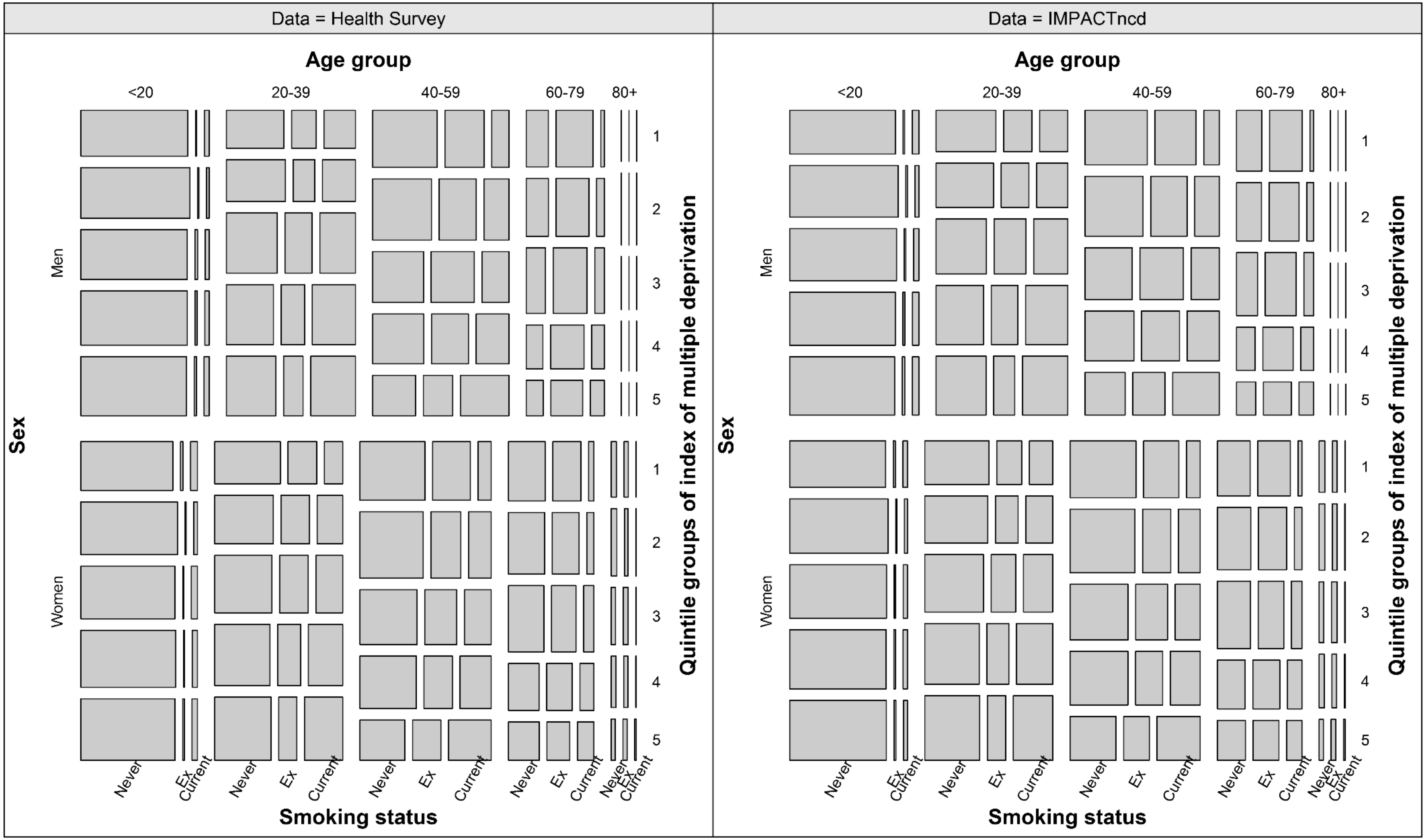

Figure S6 Comparison between the Health Survey for England 2006 (n = 17,633) and a random sample (n=200,000) from the synthetic population. Distribution of age group, sex, quintile groups of index of multiple deprivation (1=least deprived, 5=most deprived) and smoking status is presented

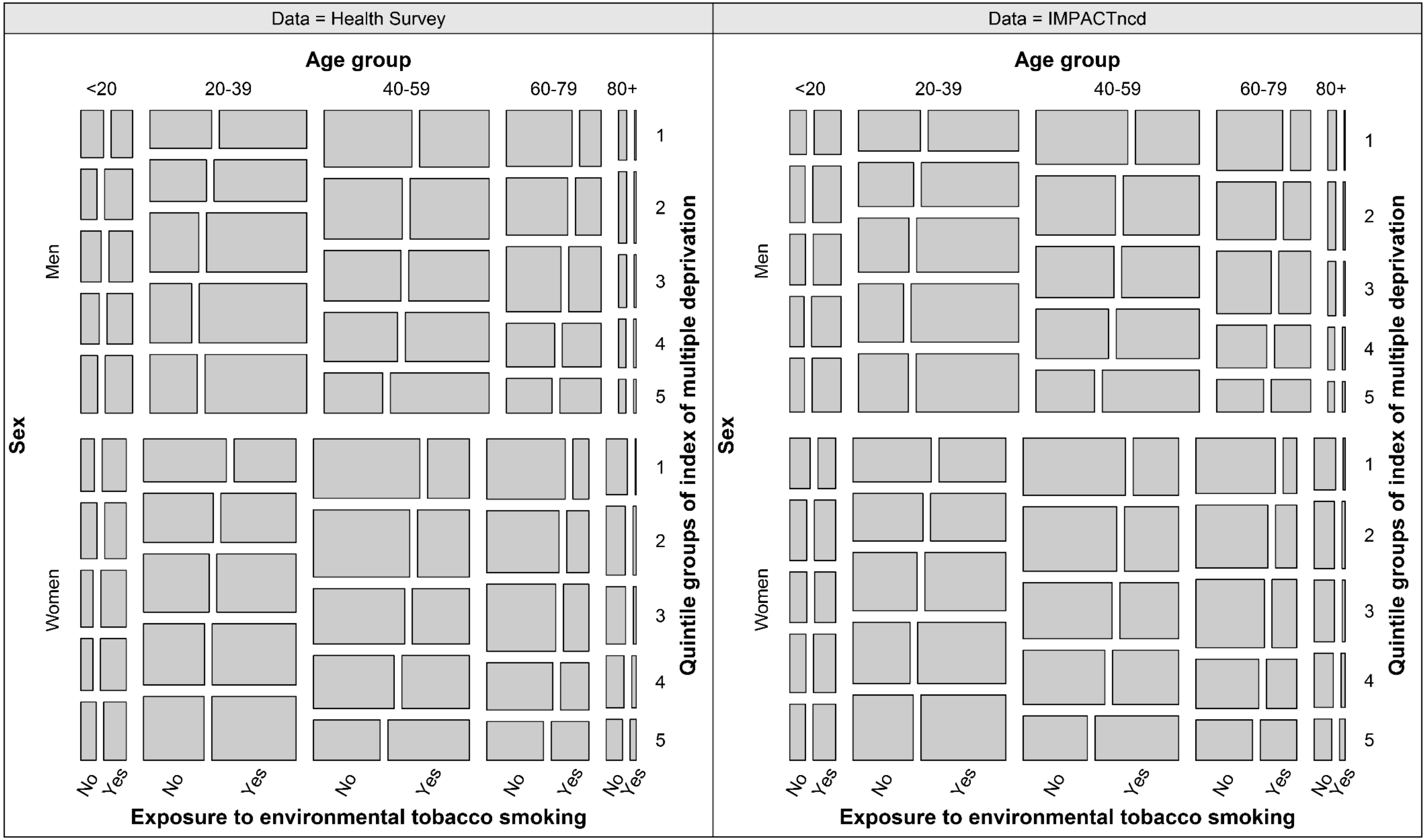

Figure S7 Comparison between the Health Survey for England 2006 (n = 17,633) and a random sample (n=200,000) from the synthetic population. Distribution of age group, sex, quintile groups of index of multiple deprivation (1=least deprived, 5=most deprived) and exposure to environmental tobacco is presented

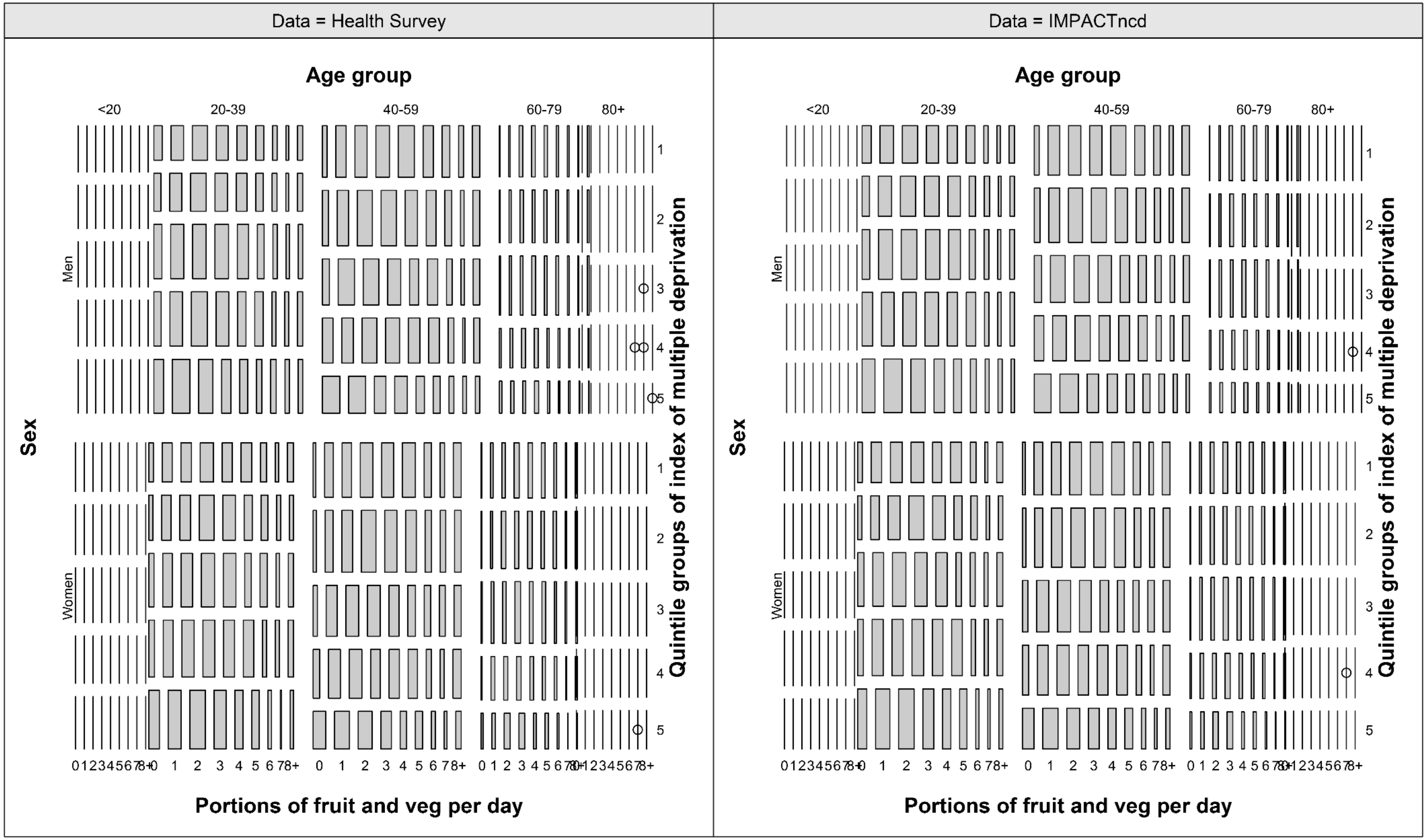

Figure S8 Comparison between the Health Survey for England 2006 (n = 17,633) and a random sample (n=200,000) from the synthetic population. Distribution of age group, sex, quintile groups of index of multiple deprivation (1=least deprived, 5=most deprived) and portions of fruit and vegetable consumed per day is presented

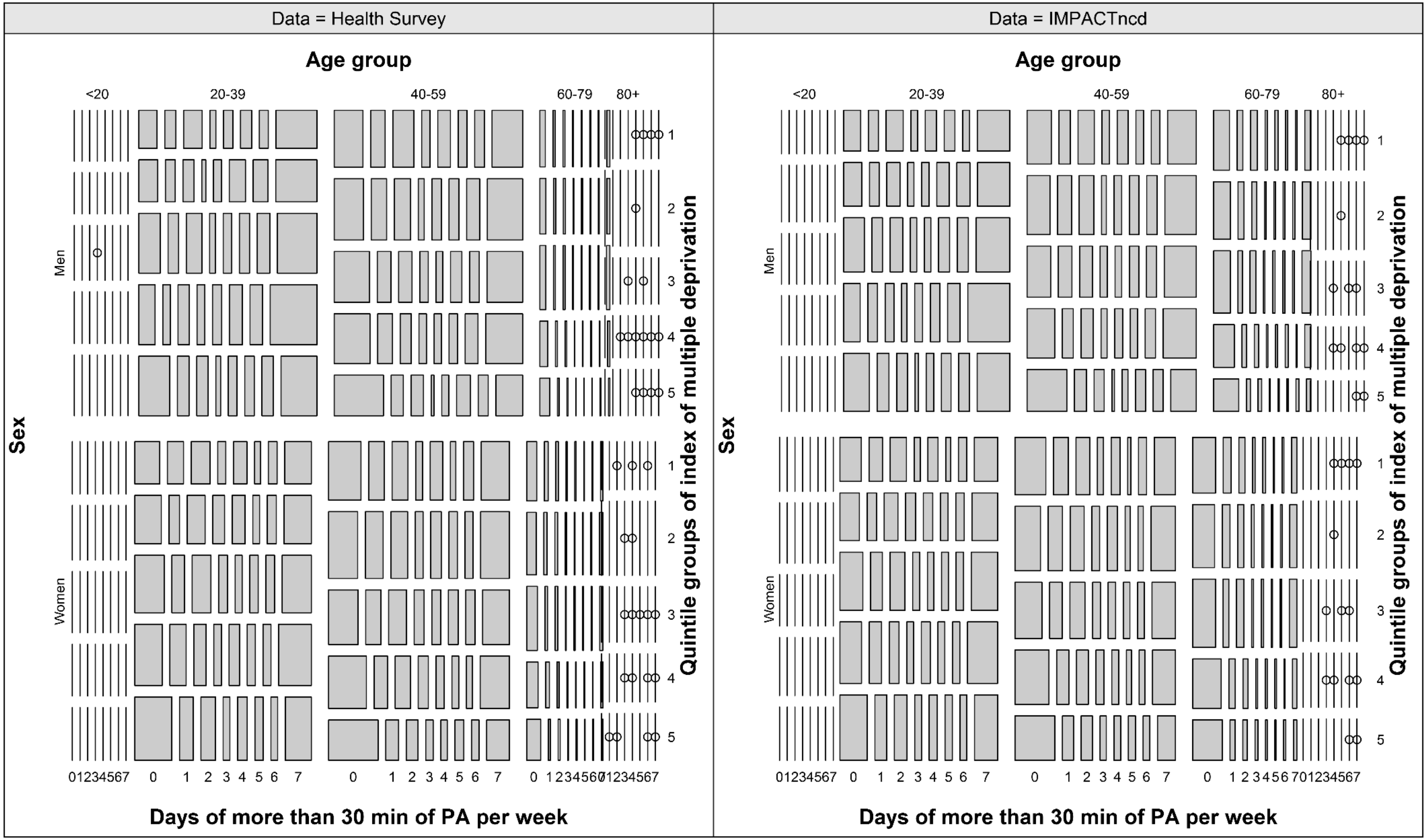

Figure S9 Comparison between the Health Survey for England 2006 (n = 17,633) and a random sample (n=200,000) from the synthetic population. Distribution of age group, sex, quintile groups of index of multiple deprivation (1=least deprived, 5=most deprived) and exposure to days of more than 30 min of physical activity (PA) per week is presented. The small circles represent sub-groups with no participants. Their number reduced in the synthetic population sample highlighting the capability of the method to create individuals with traits not present in the original survey

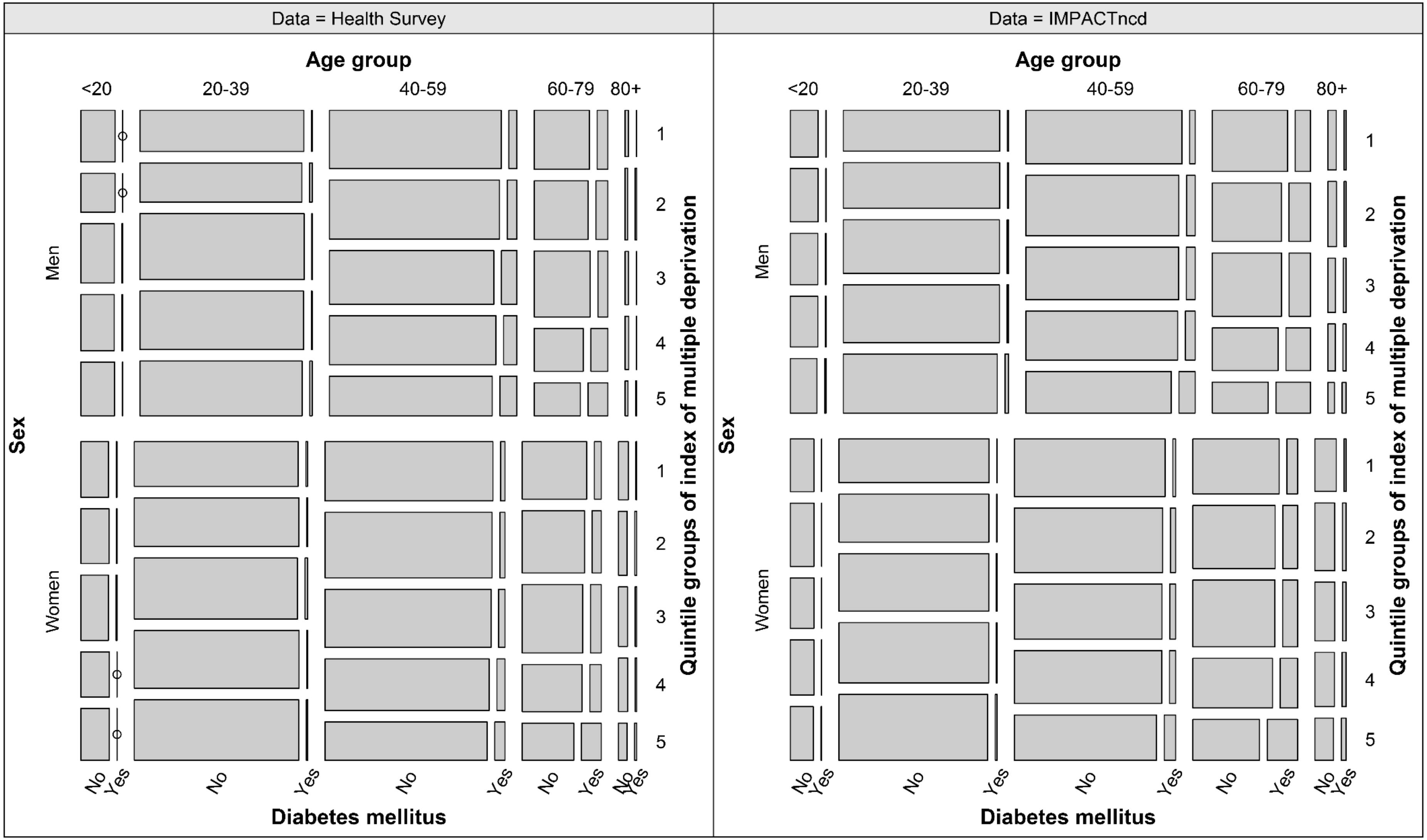

Figure S10 Comparison between the Health Survey for England 2006 (n = 17,633) and a random sample (n=200,000) from the synthetic population. Distribution of age group, sex, quintile groups of index of multiple deprivation (1=least deprived, 5=most deprived) and diabetes mellitus is presented

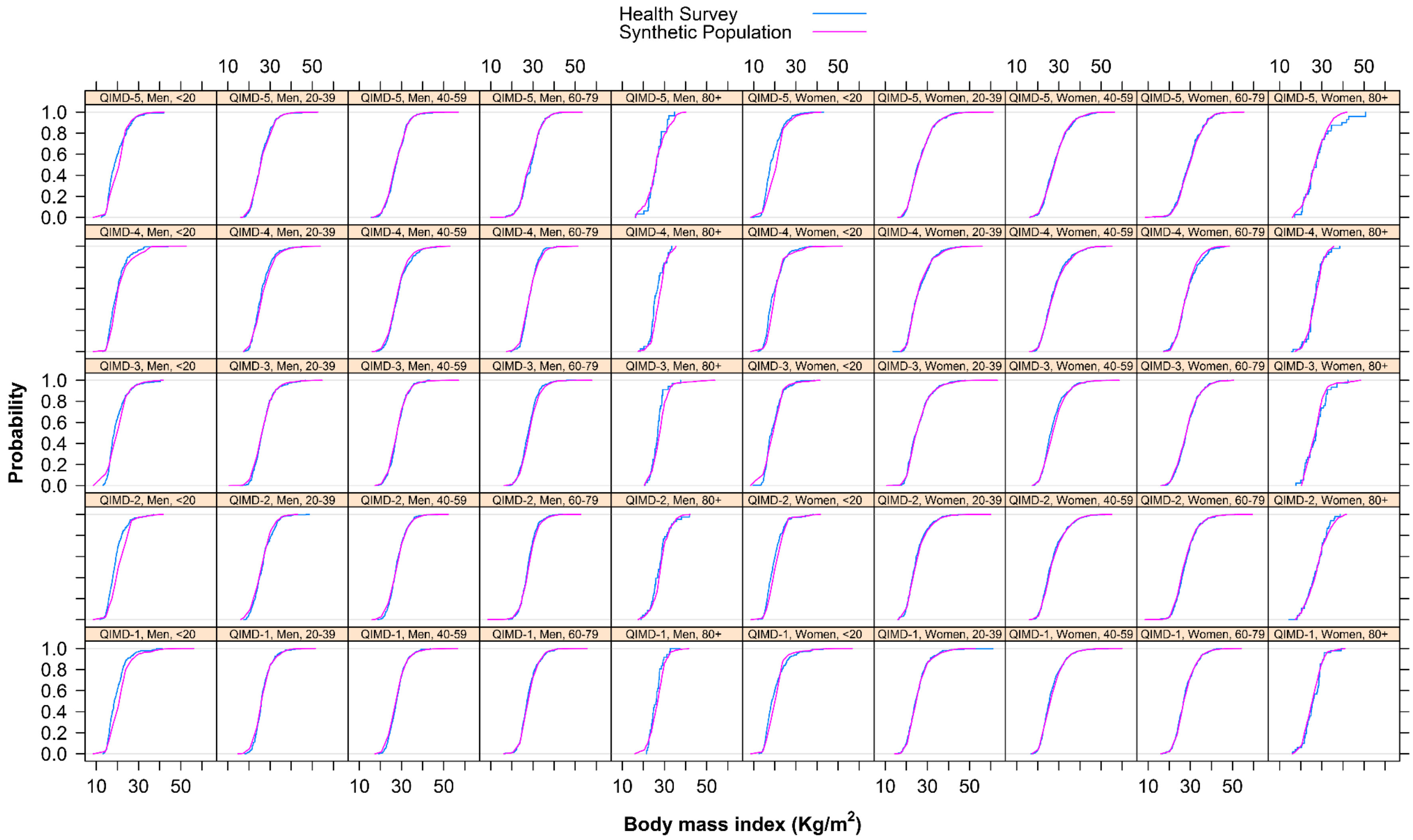

Figure S11 Comparison of body mass index cumulative distributions in Health Survey for England 2006 (n = 17,633) and a random sample (n=200,000) from the synthetic population. Each panel depicts a different subgroup of the population based on quintile groups of index of multiple deprivation (QIMD, 1=least deprived, 5=most deprived), sex and age group

=

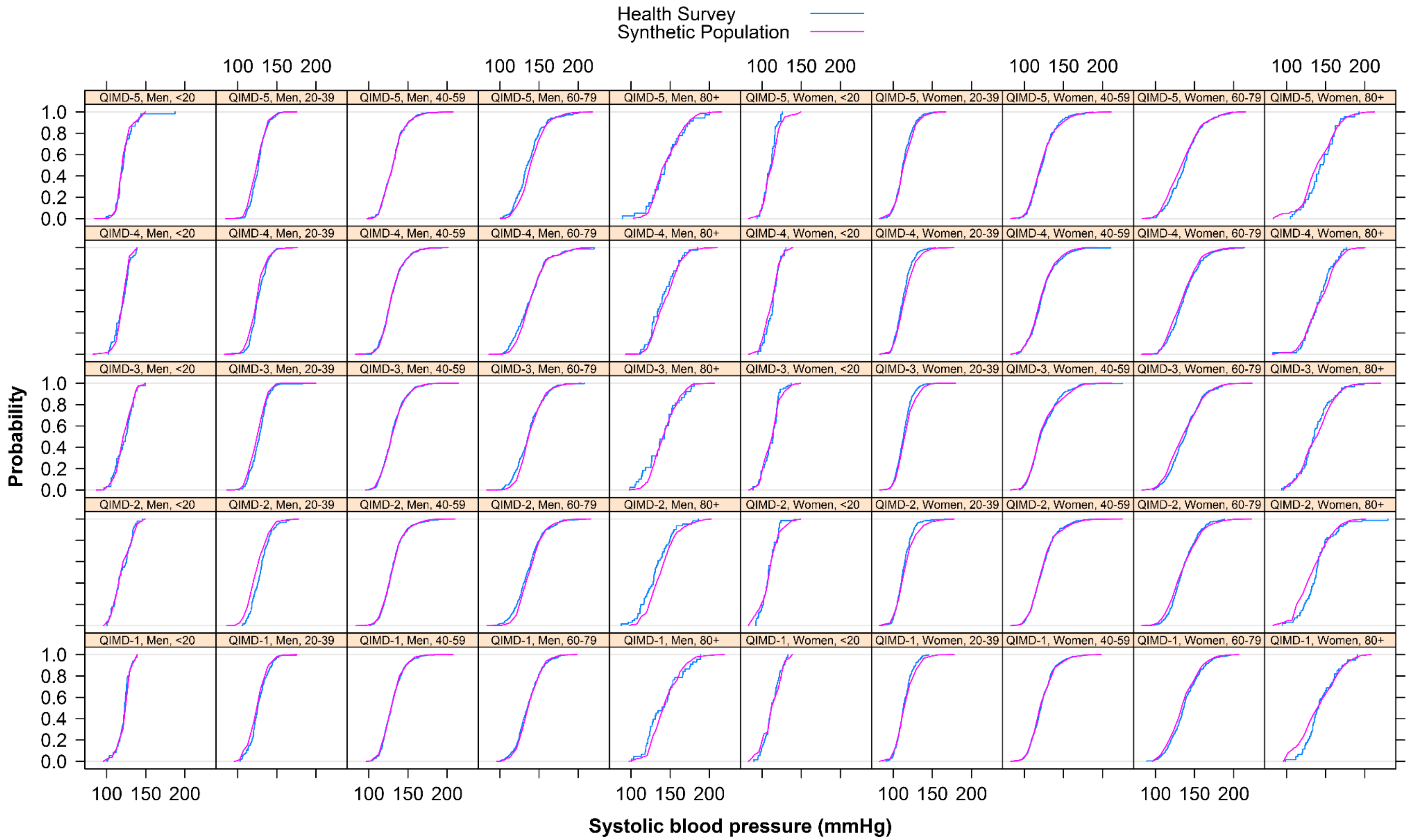

Figure S12 Comparison of systolic blood pressure cumulative distributions in Health Survey for England 2006 (n = 17,633) and a random sample (n=200,000) from the synthetic population. Each panel depicts a different subgroup of the population based on quintile groups of index of multiple deprivation (QIMD, 1=least deprived, 5=most deprived), sex and age group

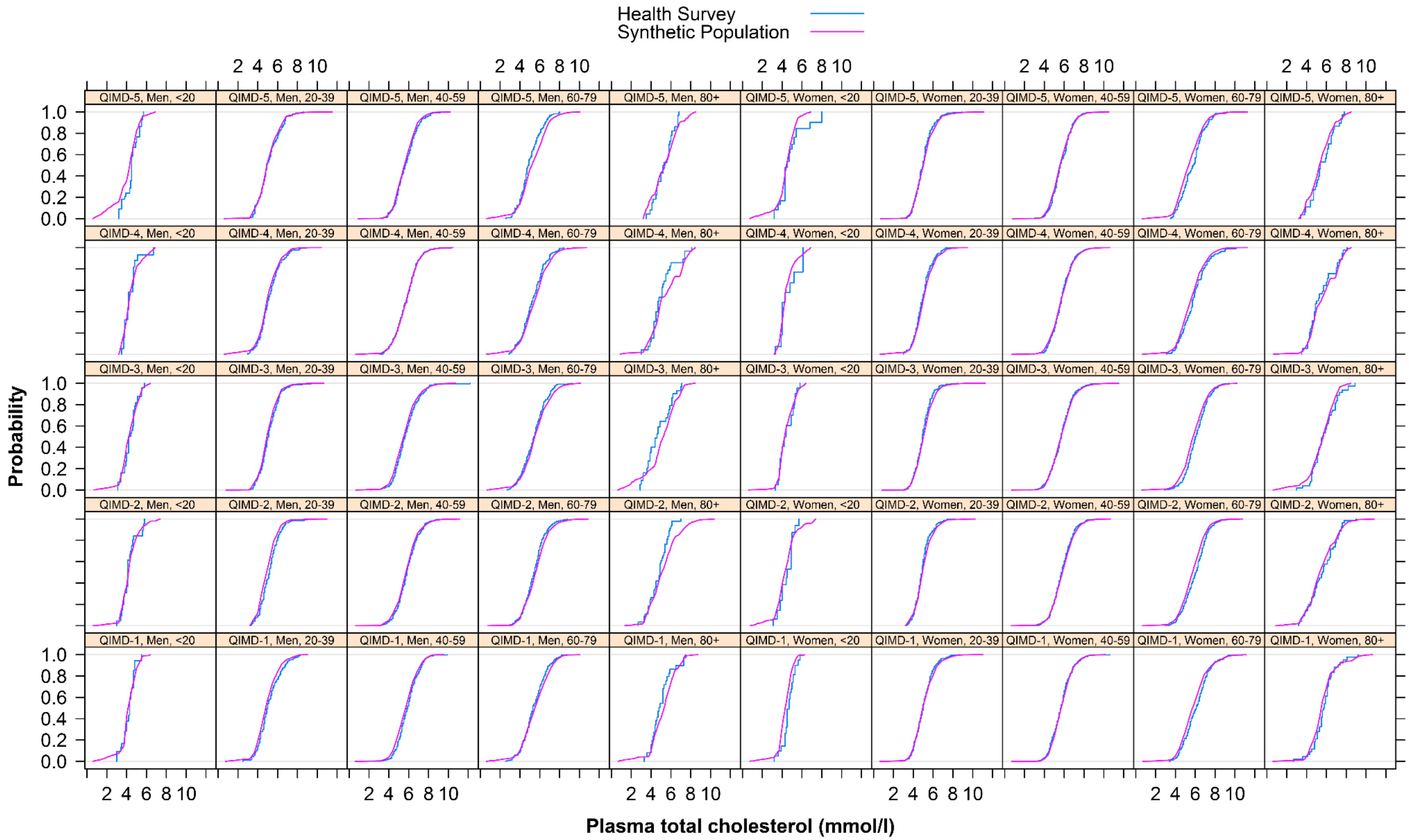

Figure S13 Comparison of plasma total cholesterol cumulative distributions in Health Survey for England 2006 (n = 17,633) and a random sample (n=200,000) from the synthetic population. Each panel depicts a different subgroup of the population based on quintile groups of index of multiple deprivation (QIMD, 1=least deprived, 5=most deprived), sex and age group

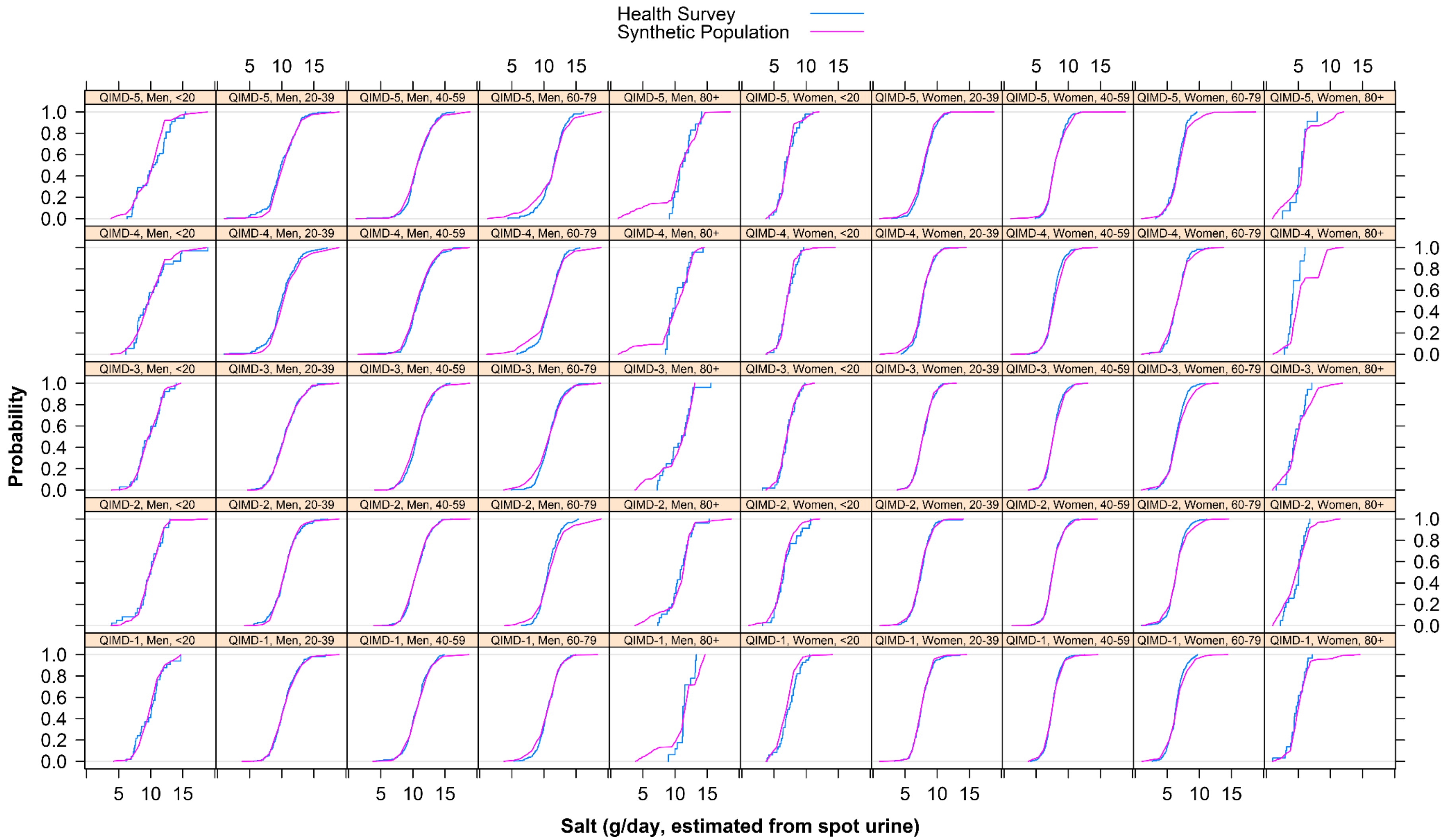

Figure S14 Comparison of salt consumption cumulative distributions in Health Survey for England 2006 (n = 17,633) and a random sample (n=200,000) from the synthetic population. Each panel depicts a different subgroup of the population based on quintile groups of index of multiple deprivation (QIMD, 1=least deprived, 5=most deprived), sex and age group. Note that IMPACT$_{NCD}$ applies another layer of processing to integrate information from 24h urine sodium measurements before risk estimation

S8.2. Risk factor trends validation

Here we compare mean exposure of IMPACT$_{NCD}$ synthetic population to the observed exposure through relevant national representative surveys. We stratified by sex, age group and when data allowed by QIMD. Overall, the plots provide evidence that the regression models used in steps 2 and 3 (Figure S1) have captured trends by age, sex and QIMD well enough.

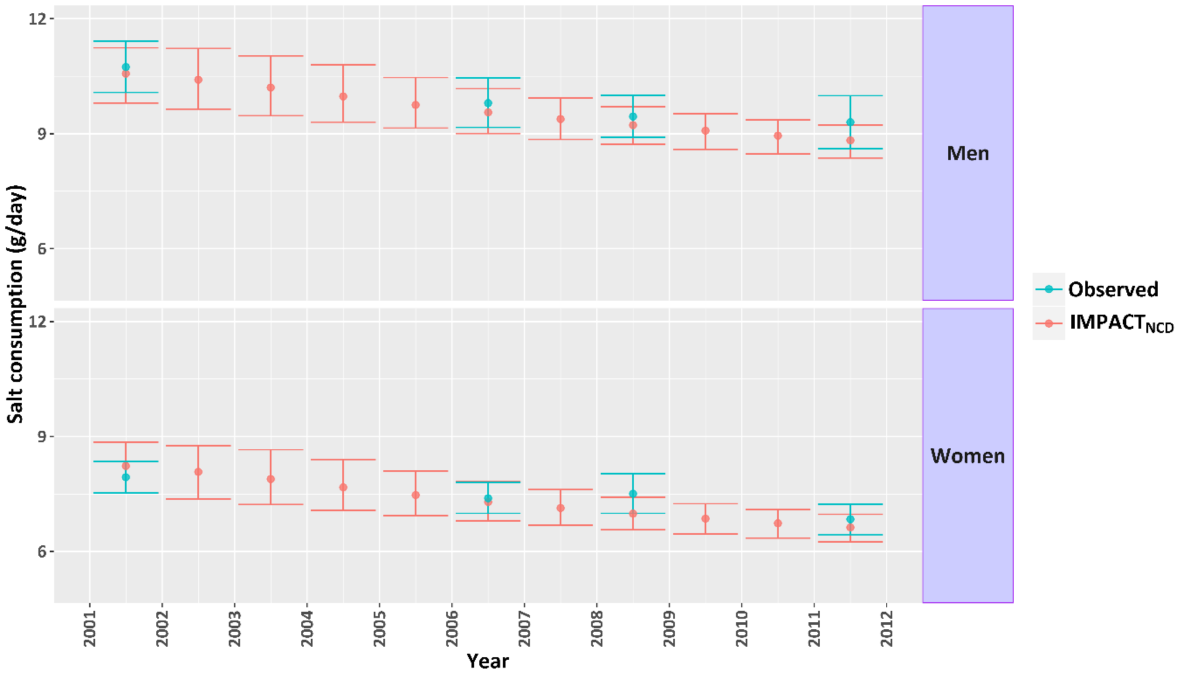

Figure S15 Mean salt consumption for ages 19 – 64 between years 2001 and 2011. Observed in the population through surveys using 24h urine collections[55–58] vs. IMPACT$_{NCD}$ synthetic population estimates. Error bars represent 95% confidence intervals of the mean.

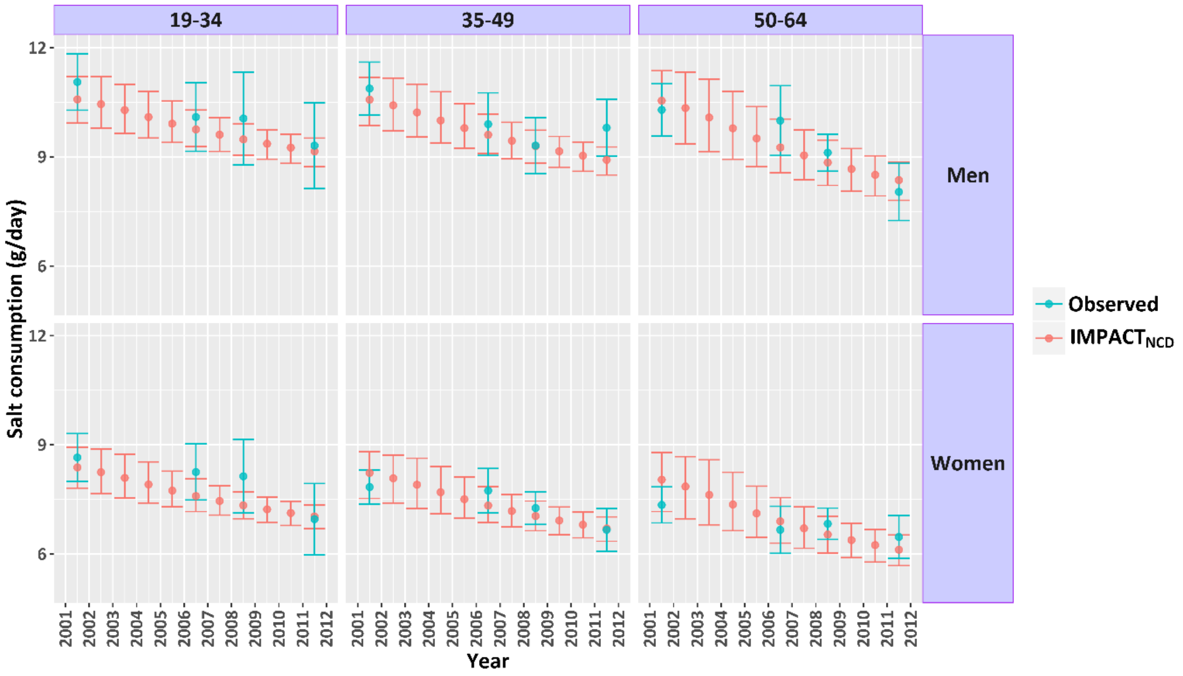

Figure S16 Mean salt consumption by age group, between years 2001 and 2011. Observed in the population through surveys using 24h urine collections[55–58] vs. IMPACT$_{NCD}$ synthetic population estimates. Error bars represent 95% confidence intervals of the mean.

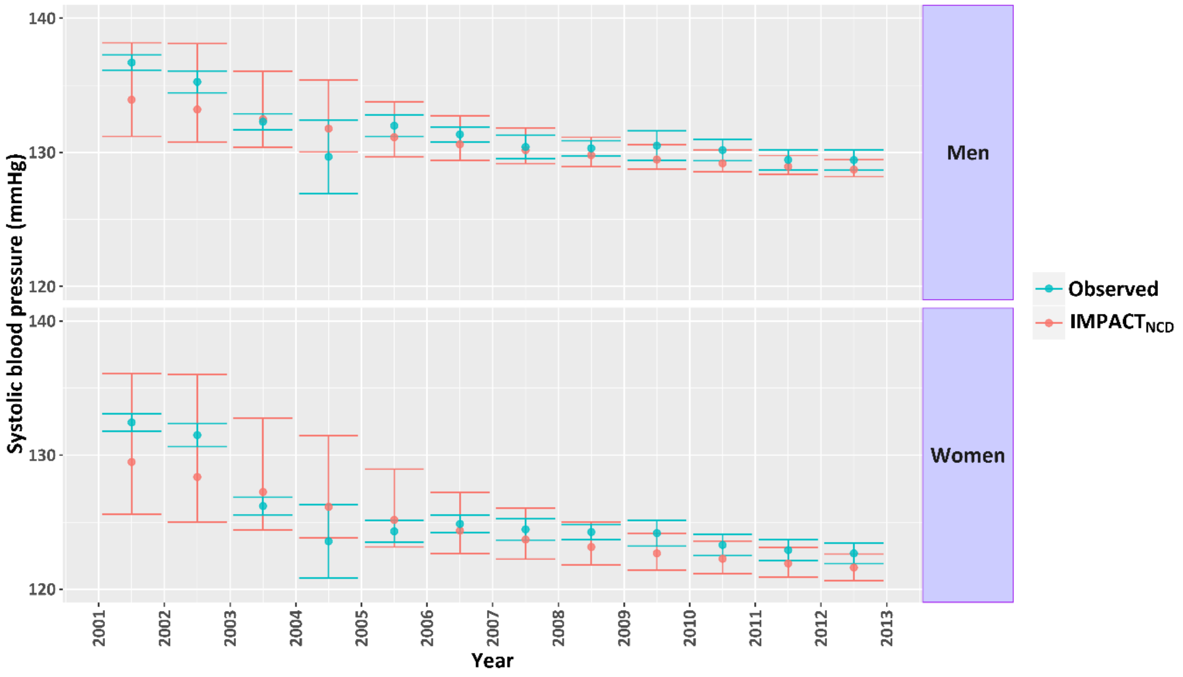

Figure S17 Mean systolic blood pressure for ages 30 – 84 between years 2001 and 2012. Observed in the population through Health Survey for England vs. IMPACT$_{NCD}$ synthetic population estimates. Error bars represent 95% confidence intervals of the mean.

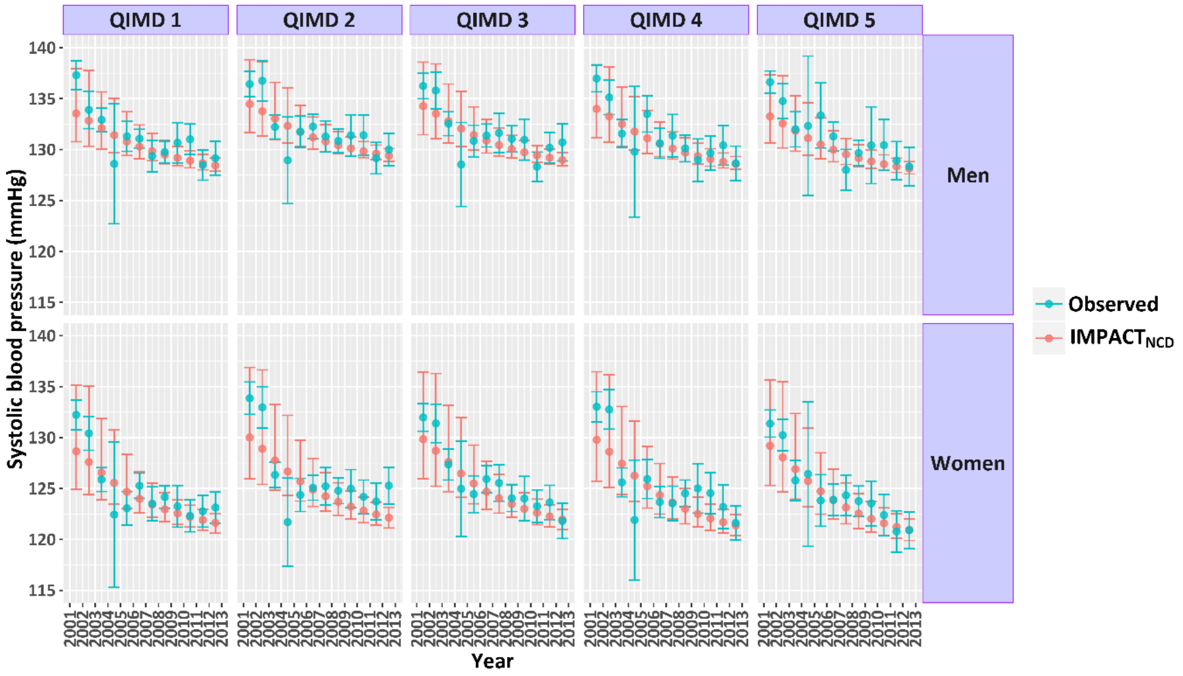

Figure S18 Mean systolic blood pressure for ages 30 – 84 by quintile group of the index of multiple deprivation (QIMD, 1 = least deprived) between years 2001 and 2012. Observed in the population through Health Survey for England vs. IMPACT$_{NCD}$ synthetic population estimates. Error bars represent 95% confidence intervals of the mean.

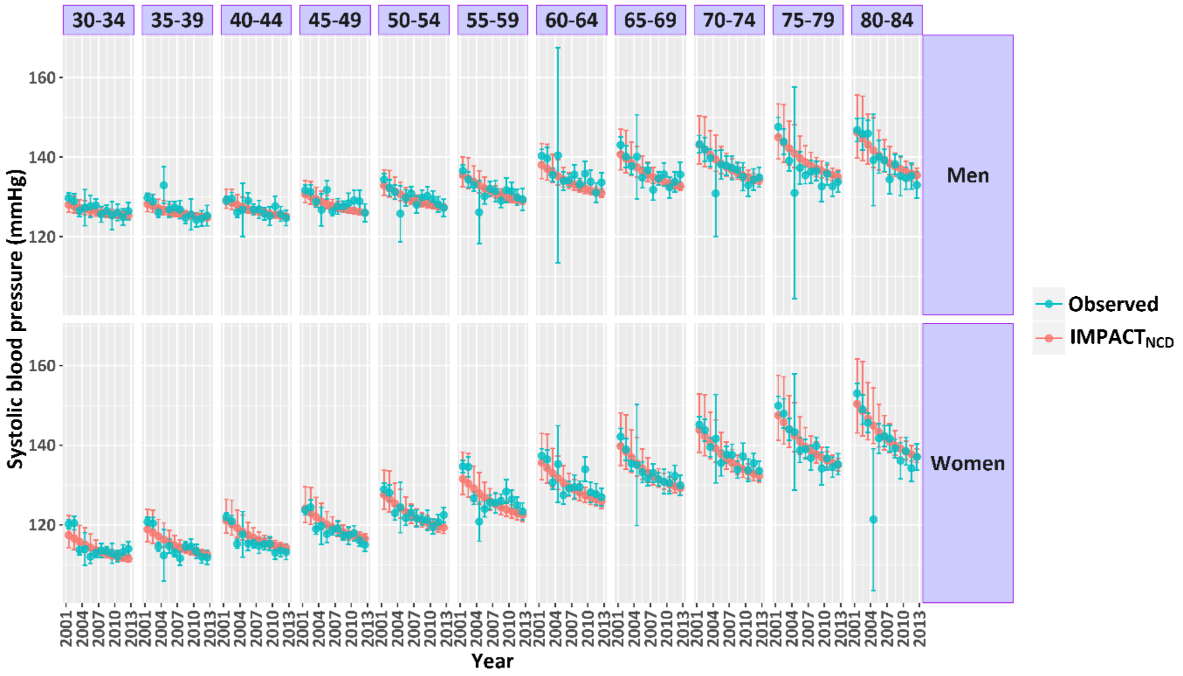

Figure S19 Mean systolic blood pressure for ages 30 – 84 by age group, between years 2001 and 2012. Observed in the population through Health Survey for England vs. IMPACT$_{NCD}$ synthetic population estimates. Error bars represent 95% confidence intervals of the mean.

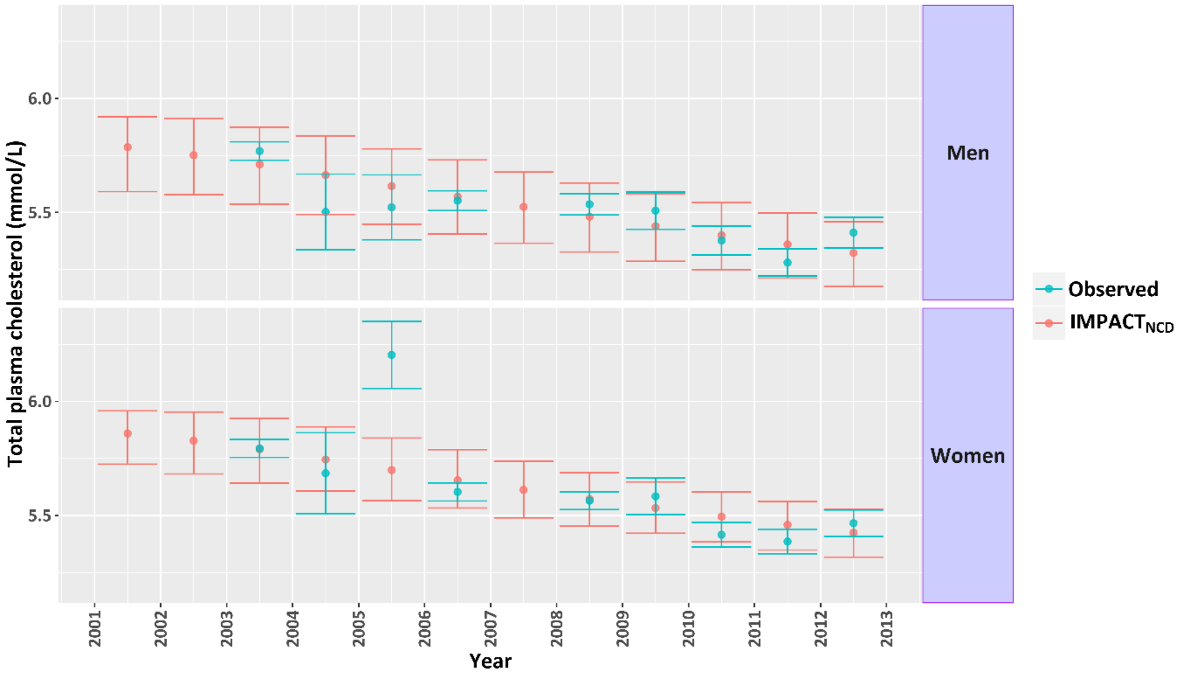

Figure S20 Mean total plasma cholesterol for ages 30 – 84 between years 2001 and 2012. Observed in the population through Health Survey for England vs. IMPACT$_{NCD}$ synthetic population estimates. Error bars represent 95% confidence intervals of the mean.

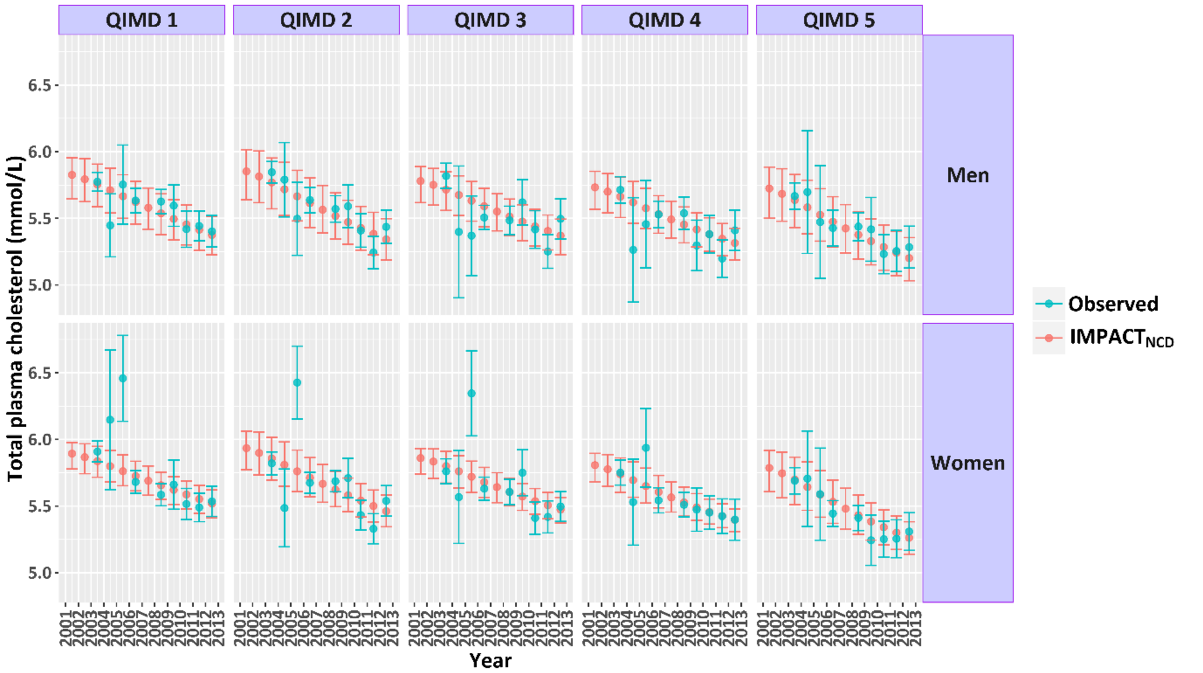

Figure S21 Mean total plasma cholesterol for ages 30 – 84 by quintile group of the index of multiple deprivation (QIMD, 1 = least deprived) between years 2001 and 2012. Observed in the population through Health Survey for England vs. IMPACT$_{NCD}$ synthetic population estimates. Error bars represent 95% confidence intervals of the mean.

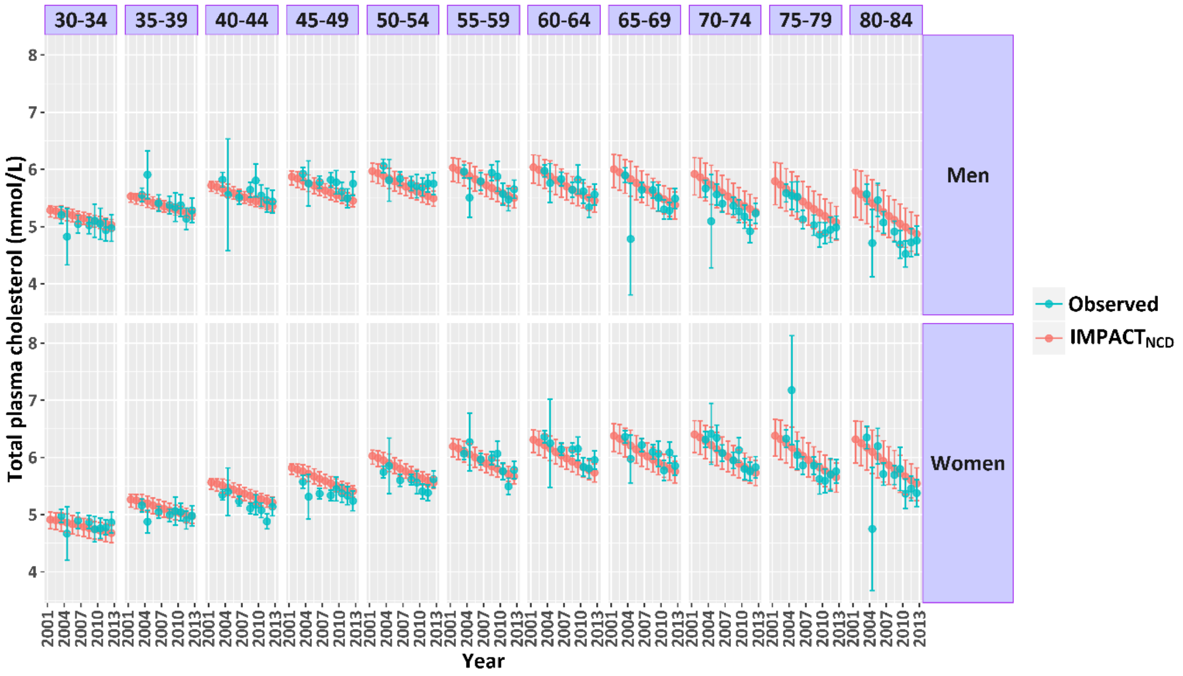

Figure S22 Mean total plasma cholesterol for ages 30 – 84 by age group, between years 2001 and 2012. Observed in the population through Health Survey for England vs. IMPACT$_{NCD}$ synthetic population estimates. Error bars represent 95% confidence intervals of the mean.

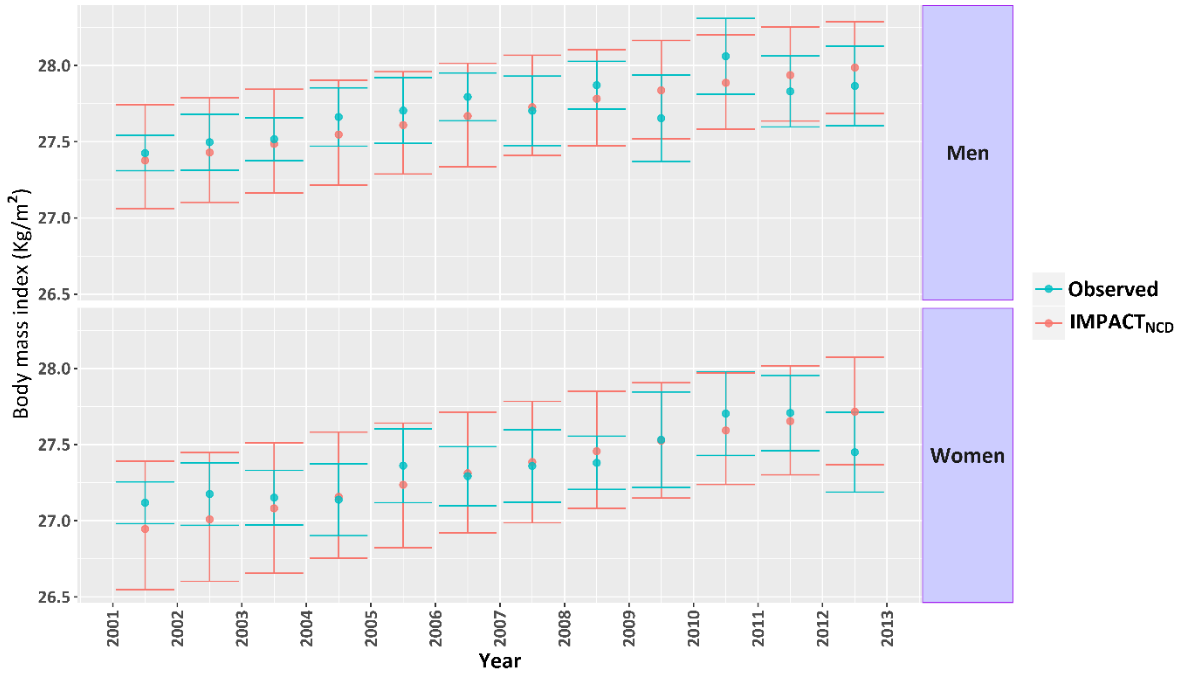

Figure S23 Mean body mass index for ages 30 – 84 between years 2001 and 2012. Observed in the population through Health Survey for England vs. IMPACT$_{NCD}$ synthetic population estimates. Error bars represent 95% confidence intervals of the mean.

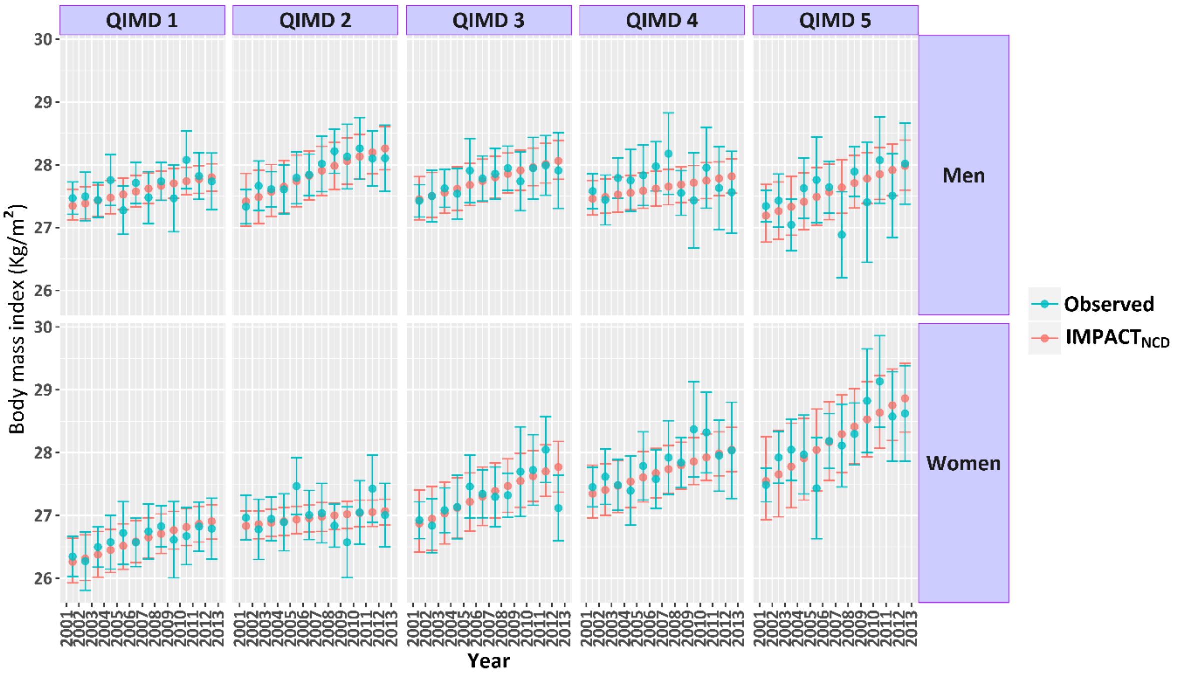

Figure S24 Mean body mass index for ages 30 – 84 by quintile group of the index of multiple deprivation (QIMD, 1 = least deprived) between years 2001 and 2012. Observed in the population through Health Survey for England vs. IMPACT$_{NCD}$ synthetic population estimates. Error bars represent 95% confidence intervals of the mean.

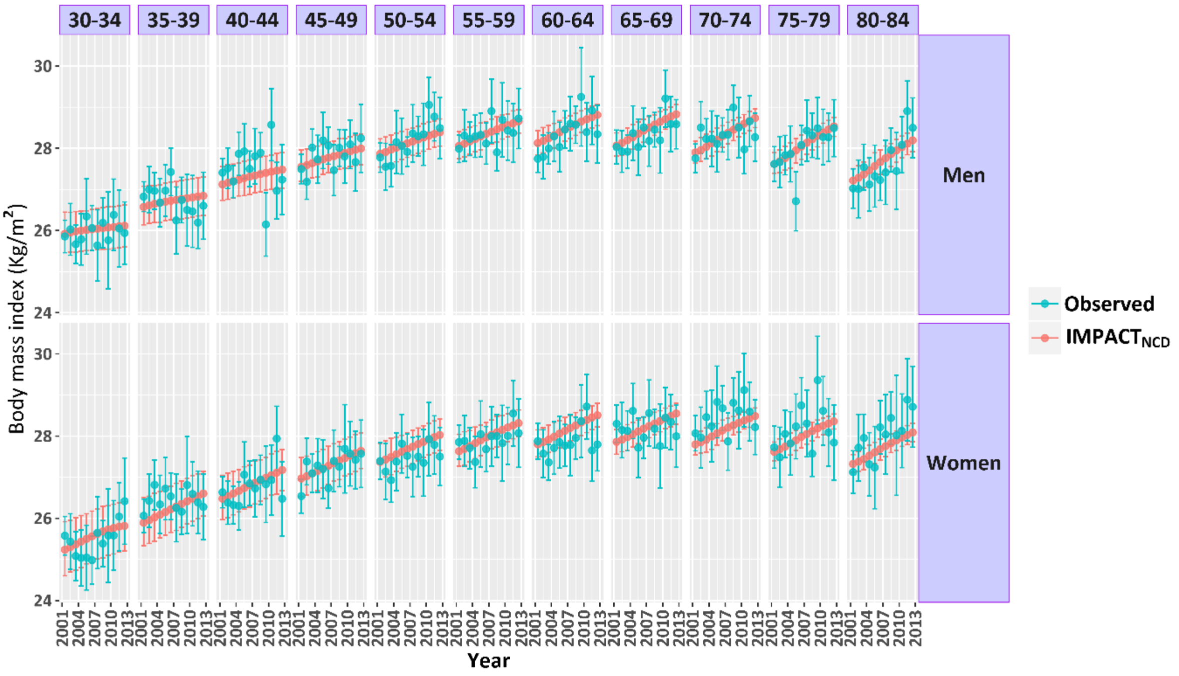

Figure S25 Mean body mass index for ages 30 – 84 by age group between years 2001 and 2012. Observed in the population through Health Survey for England vs. IMPACT$_{NCD}$ synthetic population estimates. Error bars represent 95% confidence intervals of the mean.

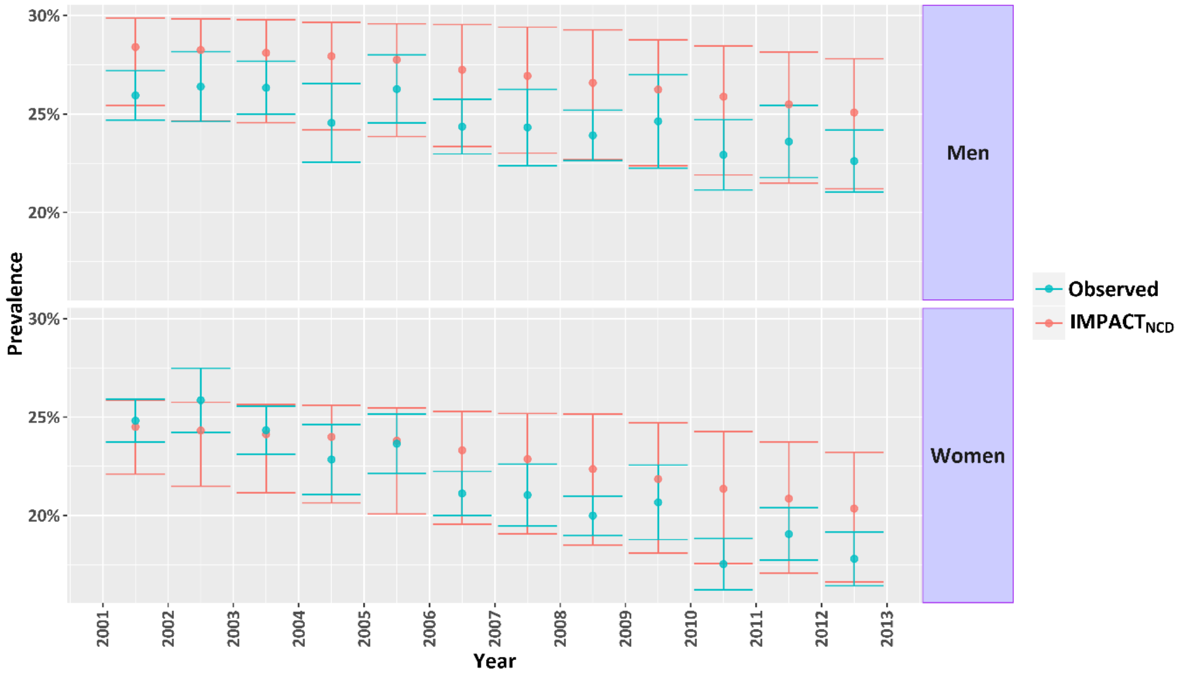

Figure S26 Smoking prevalence for ages 30 – 84 between years 2001 and 2012. Observed in the population through Health Survey for England vs. IMPACT$_{NCD}$ synthetic population estimates. Error bars represent 95% confidence intervals of the mean.

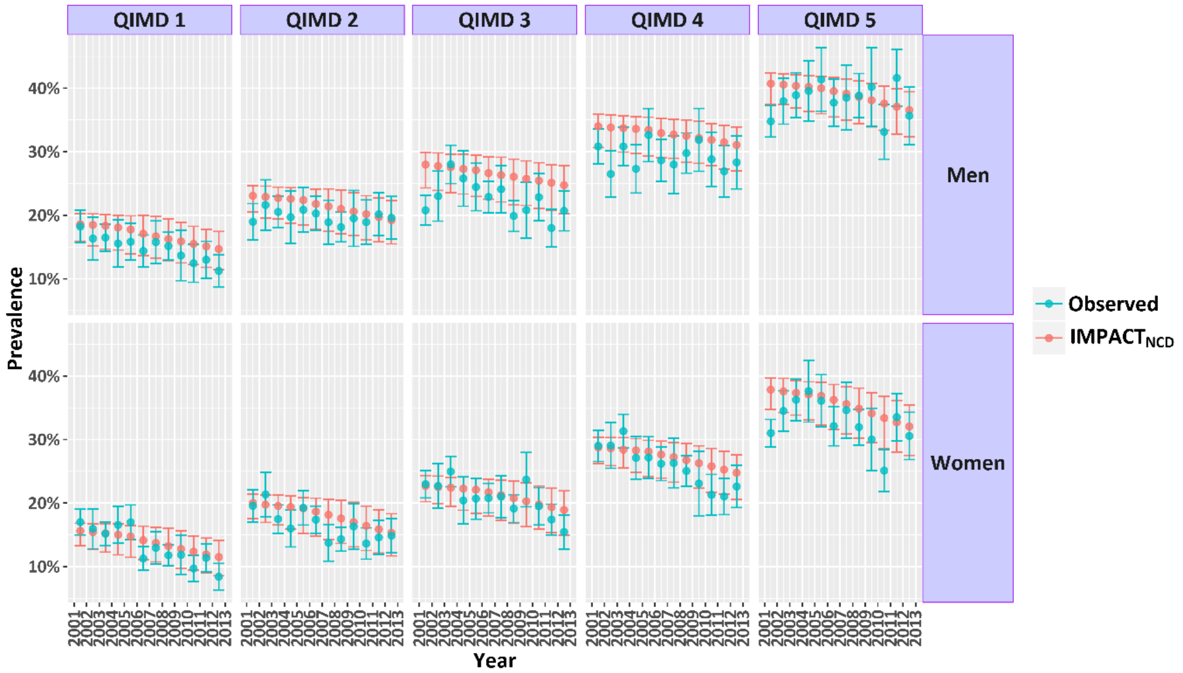

Figure S27 Smoking prevalence for ages 30 – 84 by quintile group of the index of multiple deprivation (QIMD, 1 = least deprived) between years 2001 and 2012. Observed in the population through Health Survey for England vs. IMPACT$_{NCD}$ synthetic population estimates. Error bars represent 95% confidence intervals of the mean.

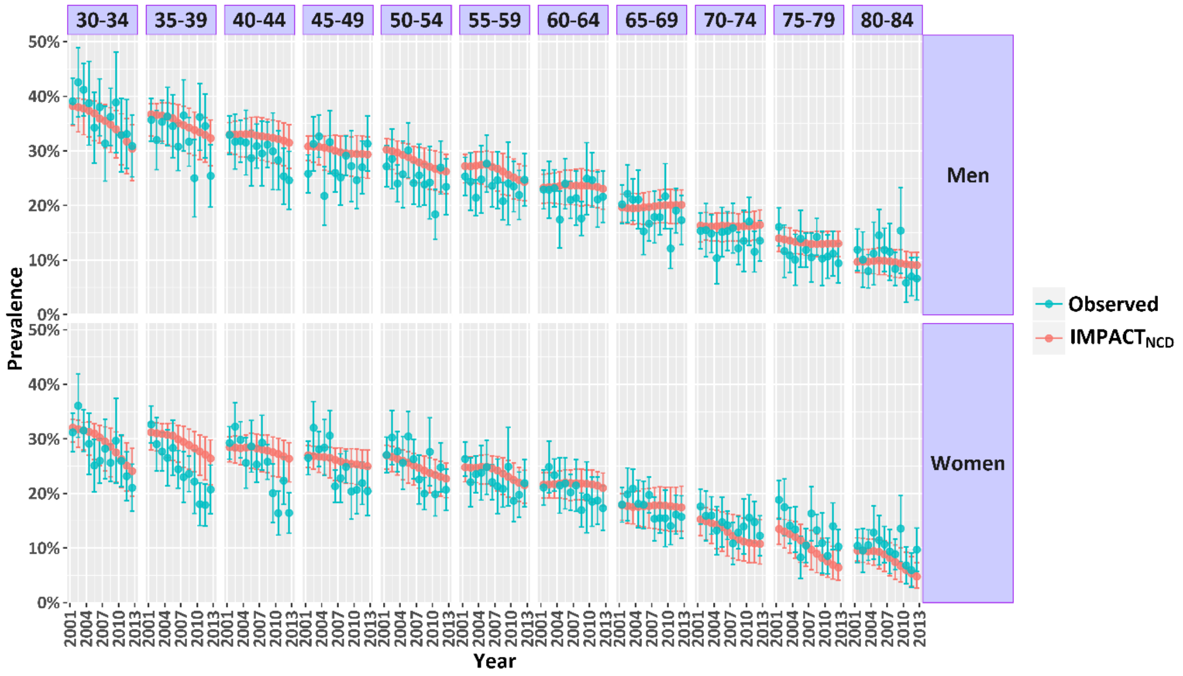

Figure S28 Smoking prevalence for ages 30 – 84 by age group between years 2001 and 2012. Observed in the population through Health Survey for England vs. IMPACT$_{NCD}$ synthetic population estimates. Error bars represent 95% confidence intervals of the mean.

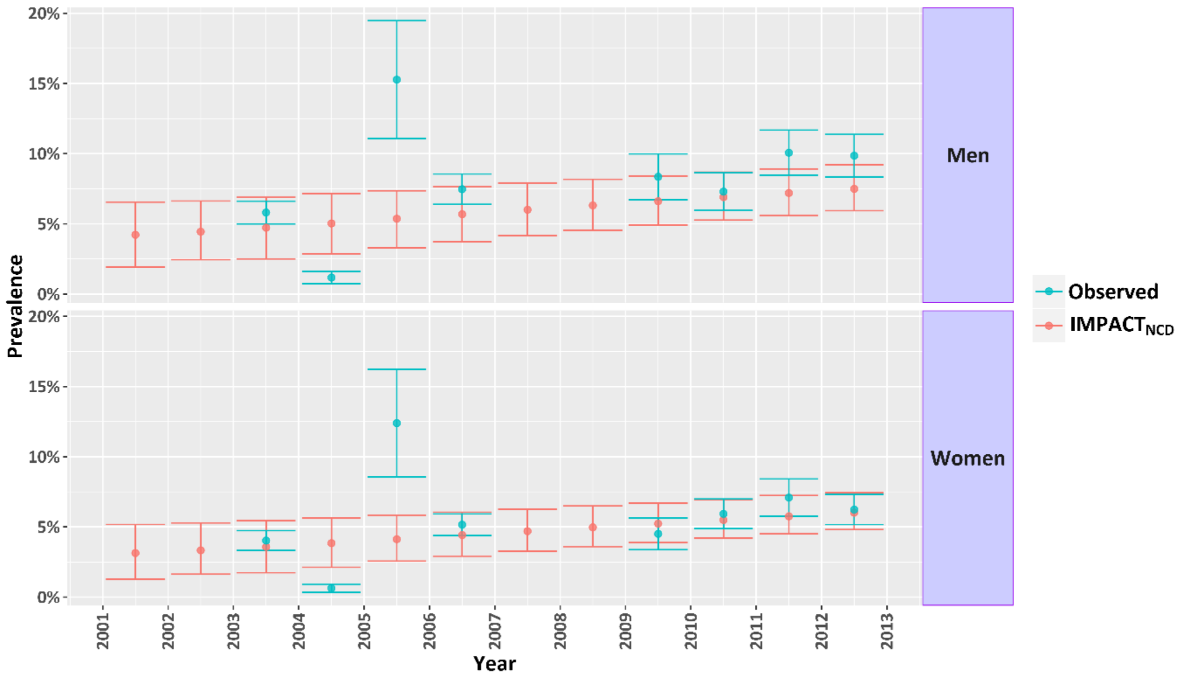

Figure S29 Diabetes mellitus prevalence for ages 30 – 84 between years 2001 and 2012. Observed in the population through Health Survey for England vs. IMPACT$_{NCD}$ synthetic population estimates. Error bars represent 95% confidence intervals of the mean.

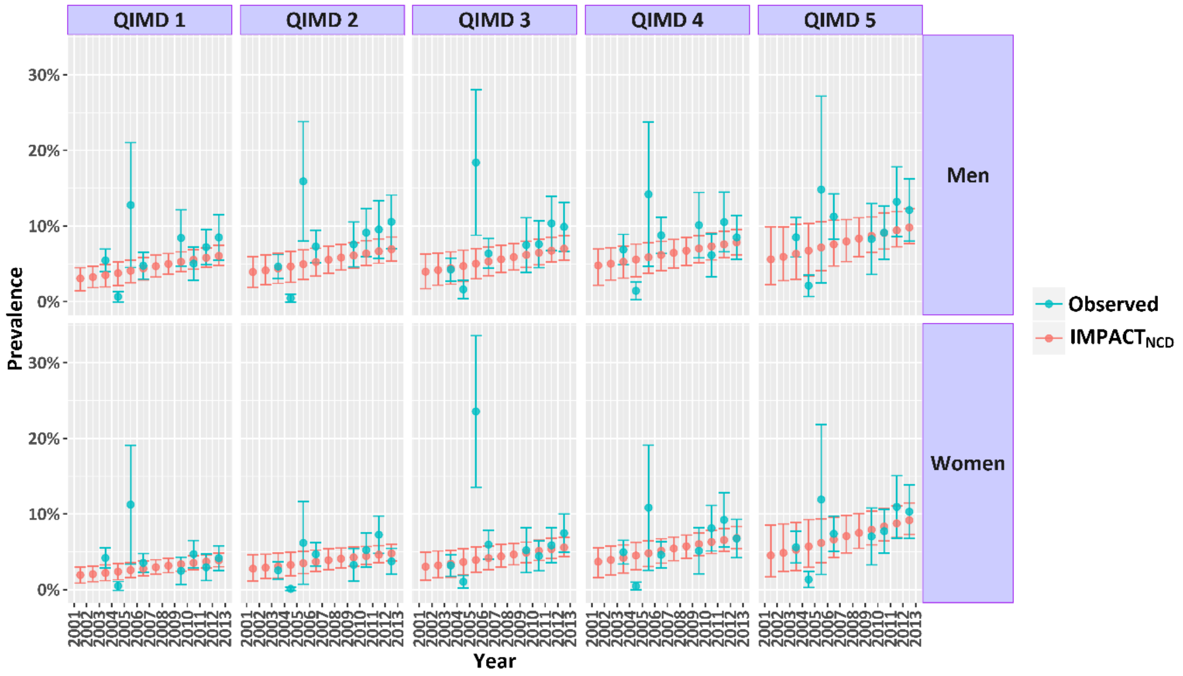

Figure S30 Diabetes mellitus prevalence for ages 30 – 84 by quintile group of the index of multiple deprivation (QIMD, 1 = least deprived) between years 2001 and 2012. Observed in the population through Health Survey for England vs. IMPACT$_{NCD}$ synthetic population estimates. Error bars represent 95% confidence intervals of the mean.

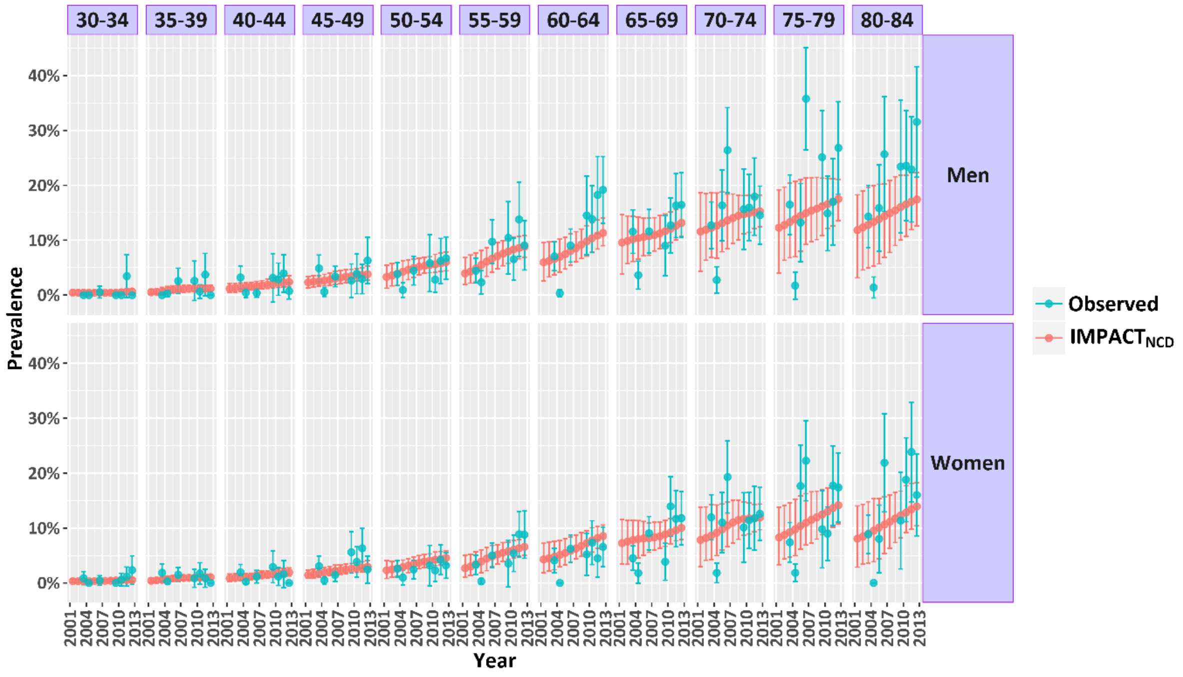

Figure S31 Diabetes mellitus prevalence for ages 30 – 84 by age group between years 2001 and 2012. Observed in the population through Health Survey for England vs. IMPACT$_{NCD}$ synthetic population estimates. Error bars represent 95% confidence intervals of the mean.

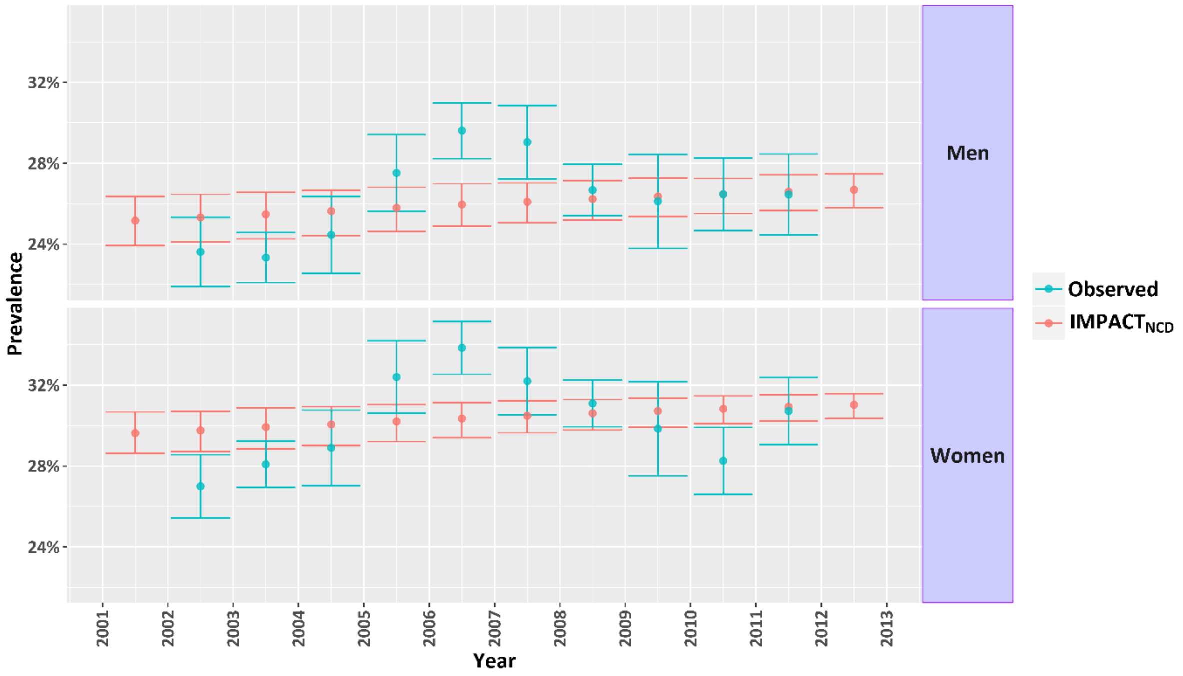

Figure S32 Five or more portions of fruit & veg per day prevalence for ages 30 – 84 between years 2001 and 2012. Observed in the population through Health Survey for England vs. IMPACT$_{NCD}$ synthetic population estimates. Error bars represent 95% confidence intervals of the mean.

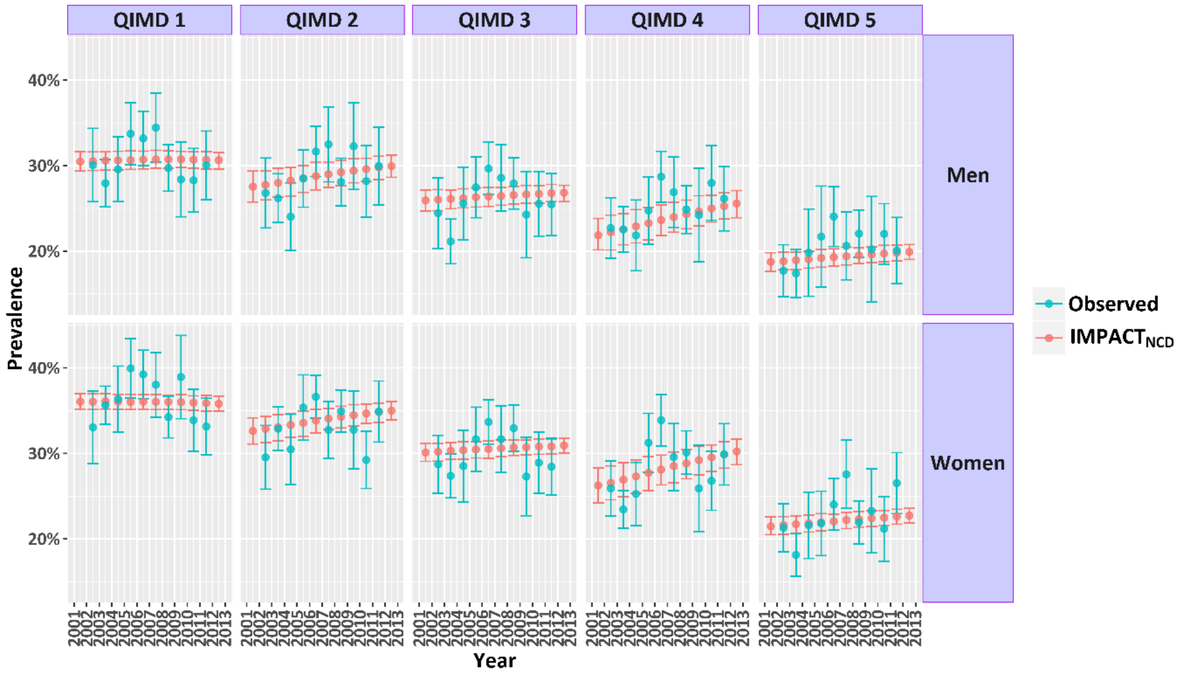

Figure S33 Five or more portions of fruit & veg per day prevalence for ages 30 – 84 by quintile group of the index of multiple deprivation (QIMD, 1 = least deprived) between years 2001 and 2012. Observed in the population through Health Survey for England vs. IMPACT$_{NCD}$ synthetic population estimates. Error bars represent 95% confidence intervals of the mean.

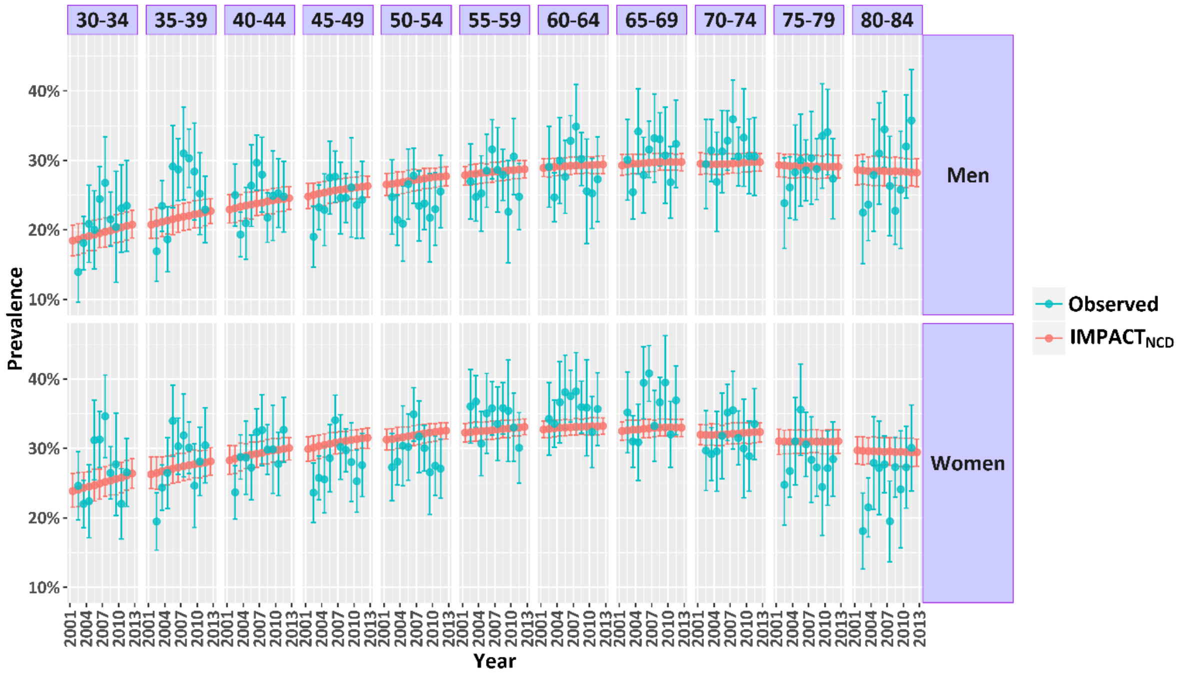

Figure S34 Five or more portions of fruit & veg per day prevalence for ages 30 – 84 by age group between years 2001 and 2012. Observed in the population through Health Survey for England vs. IMPACT$_{NCD}$ synthetic population estimates. Error bars represent 95% confidence intervals of the mean.

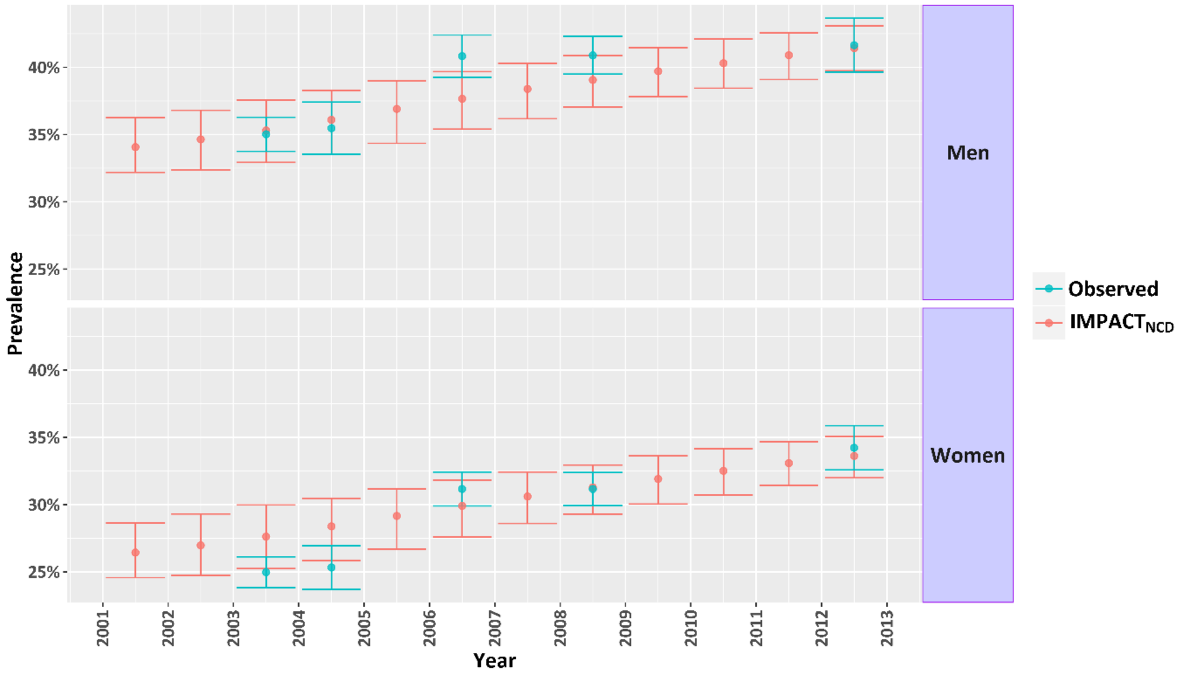

Figure S35 Five or more active days per week prevalence for ages 30 – 84 between years 2001 and 2012. Observed in the population through Health Survey for England vs. IMPACT$_{NCD}$ synthetic population estimates. Error bars represent 95% confidence intervals of the mean.

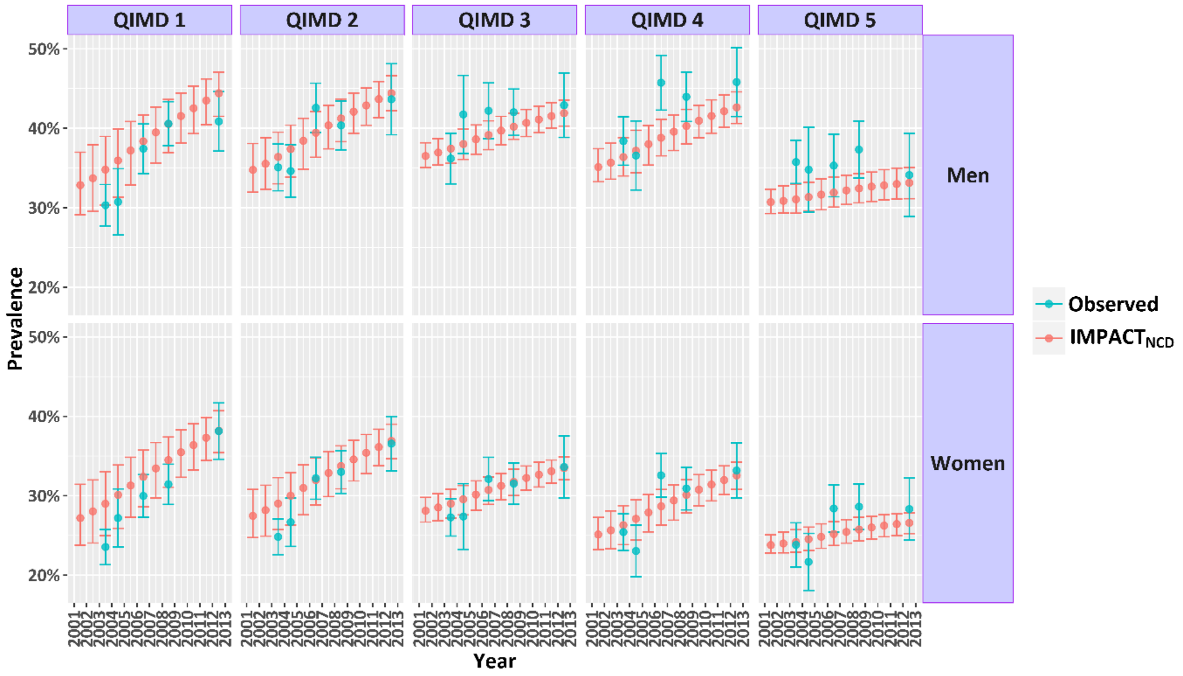

Figure S36 Five or more active days per week prevalence for ages 30 – 84 by quintile group of the index of multiple deprivation (QIMD, 1 = least deprived) between years 2001 and 2012. Observed in the population through Health Survey for England vs. IMPACT$_{NCD}$ synthetic population estimates. Error bars represent 95% confidence intervals of the mean.

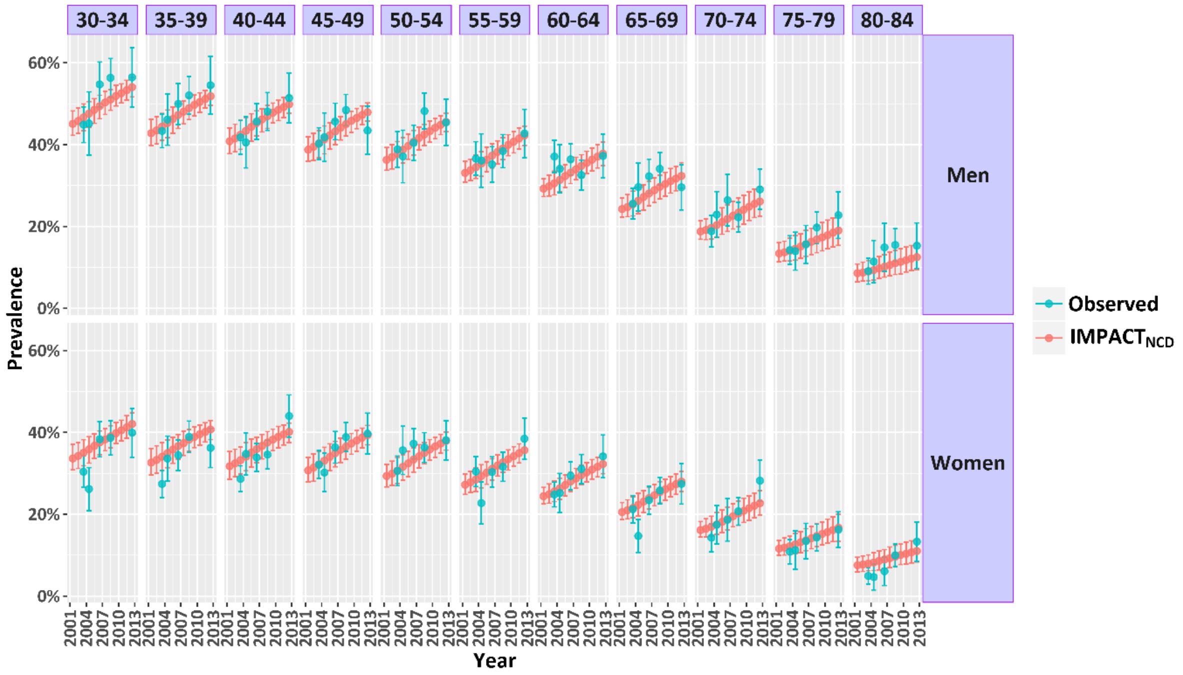

Figure S37 Five or more active days per week prevalence for ages 30 – 84 by age group between years 2001 and 2012. Observed in the population through Health Survey for England vs. IMPACT$_{NCD}$ synthetic population estimates. Error bars represent 95% confidence intervals of the mean.

S8.3. Incidence external validation

We validated incidence only for GCa, as data the observed incidence is known through the cancer registries. This was not possible for CVD as the true 'first ever' incidence is largely unknown.

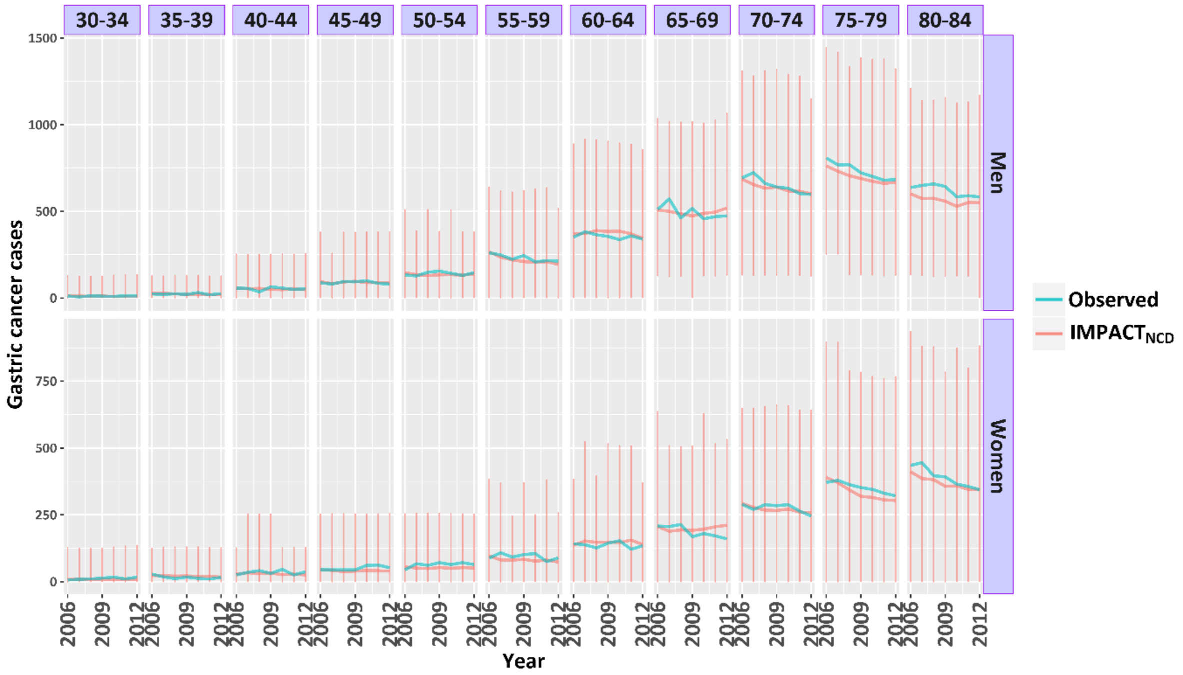

Figure S38 Gastric cancer cases in England for ages 30 – 84 by age group between years 2006 and 2012. Observed in the population through cancer registries vs. IMPACT$_{NCD}$ synthetic population estimates. Error bars represent 95% uncertainty intervals.

S8.4. Mortality external validation

Here we validate the $IMPACT_{NCD}$ estimated mortality against the observed mortality in England between 2006 and 2013. We stratify by disease, age, sex and QIMD. Overall, the plots support the argument that $IMPACT_{NCD}$ is capable of translating changes in risk factors prevalence into changes in disease incidence and mortality, rather accurately.

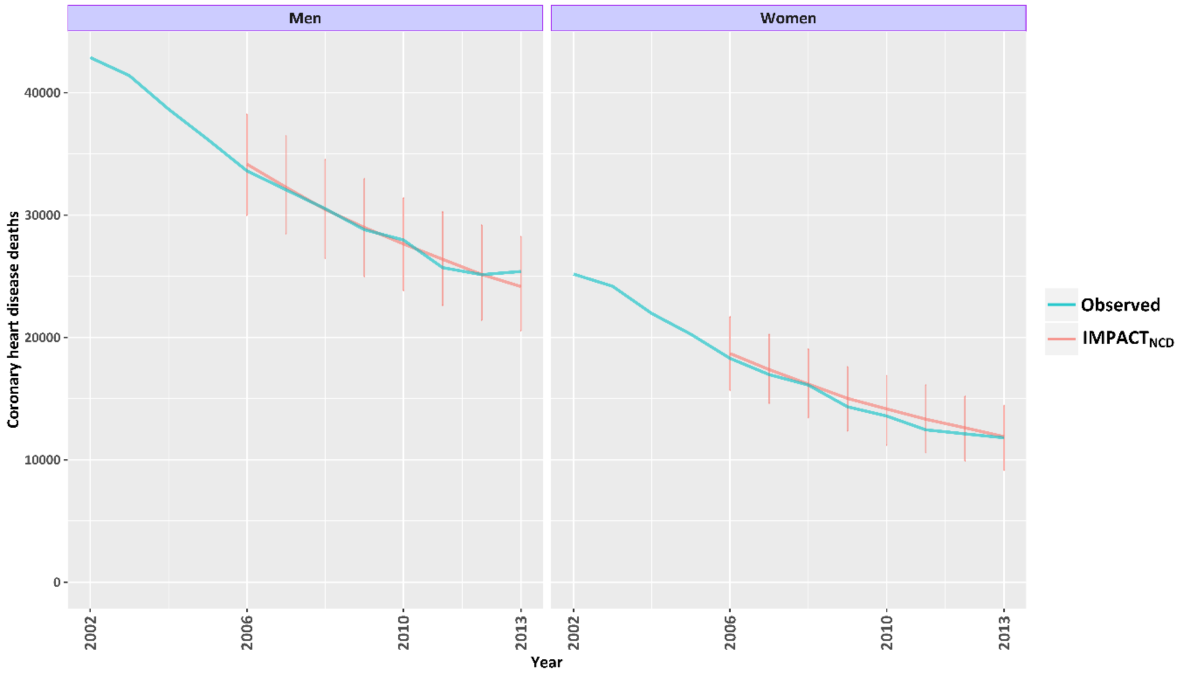

Figure S39 Number of deaths from coronary heart disease in England, by year and sex for ages 30 to 84. Office for National Statistics reported deaths (observed) vs $IMPACT_{NCD}$ estimated

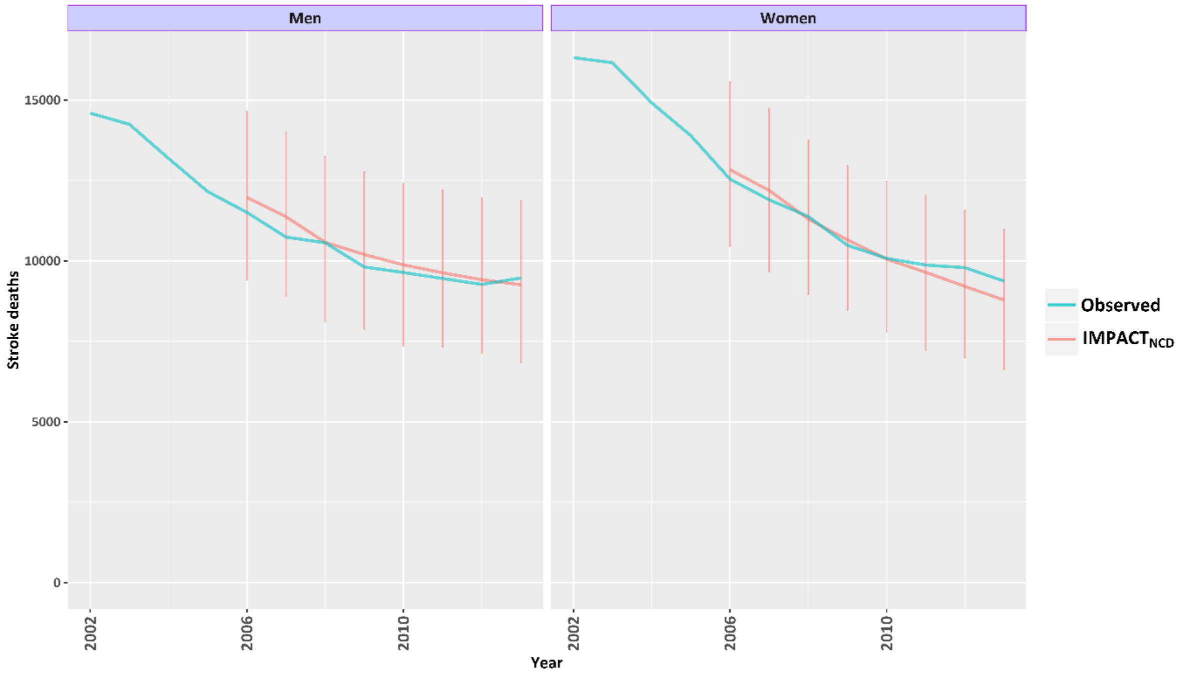

Figure S40 Number of deaths from stroke in England, by year and sex for ages 30 to 84. Office for National Statistics (ONS) reported deaths (observed) vs $IMPACT_{NCD}$ estimated. Observed deaths after 2010 were adjusted to account for changes in the ICD-10 version used by ONS since 201. Error bars represent interquartile ranges.

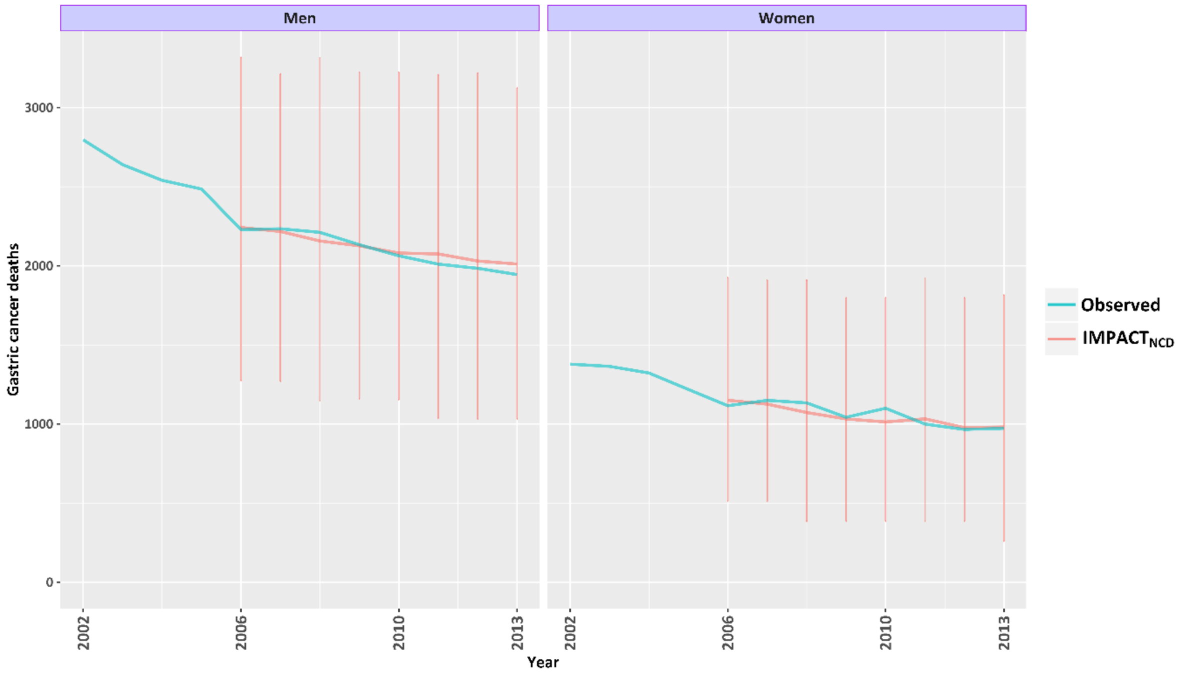

Figure S 41 Number of deaths from gastric cancer in England, by year and sex for ages 30 to 84. Office for National Statistics reported deaths (observed) vs $IMPACT_{NCD}$ estimated.

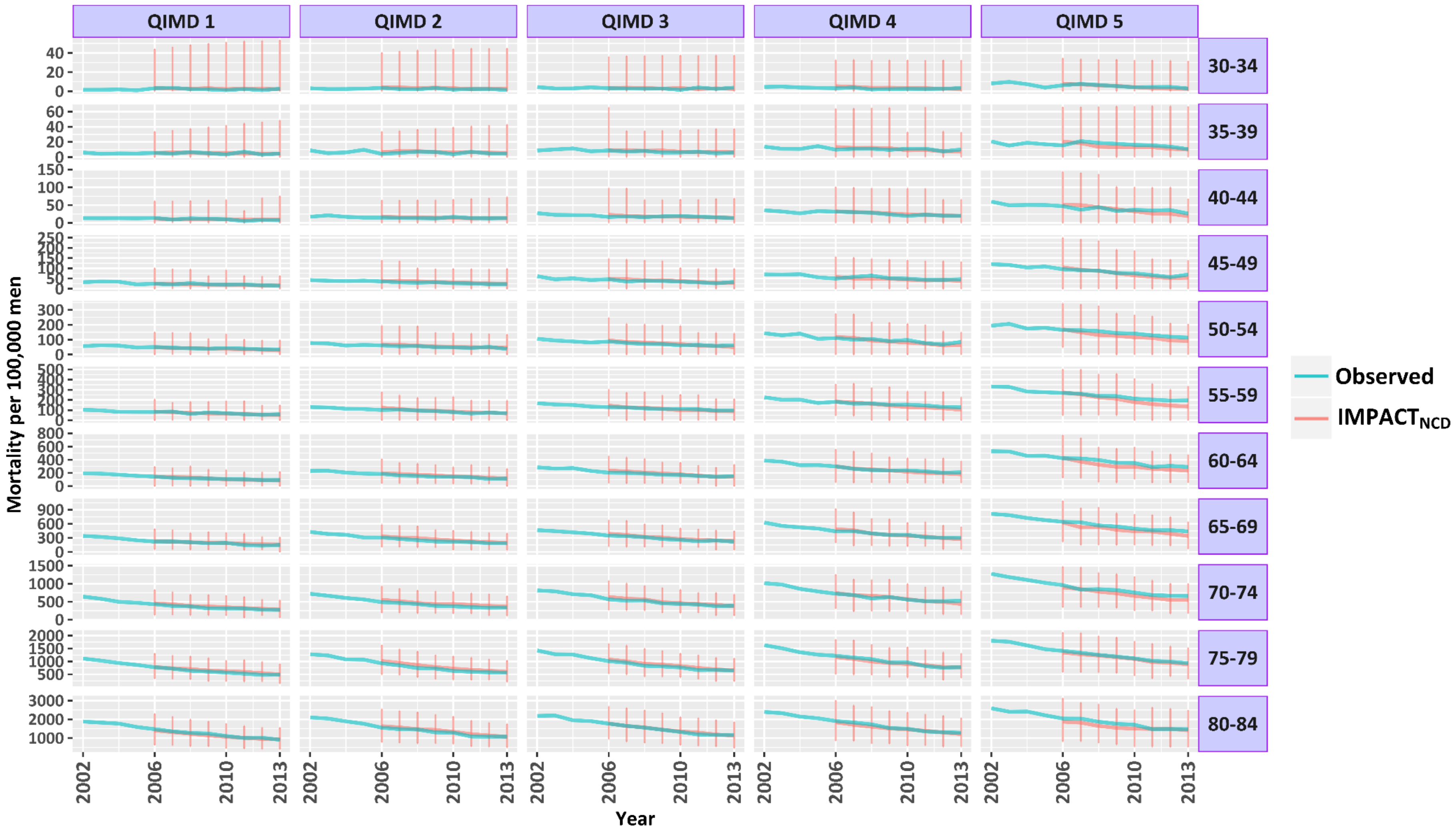

Figure S42 Coronary heart disease mortality (ICD10: I20 – I25) for men by age group and quintile group of index of multiple deprivation (QIMD, 1 = least deprived) between years 2002 and 2013. Observed in the population through mortality registries vs. $IMPACT_{NCD}$ synthetic population estimates. Whiskers represent 95% uncertainty intervals.

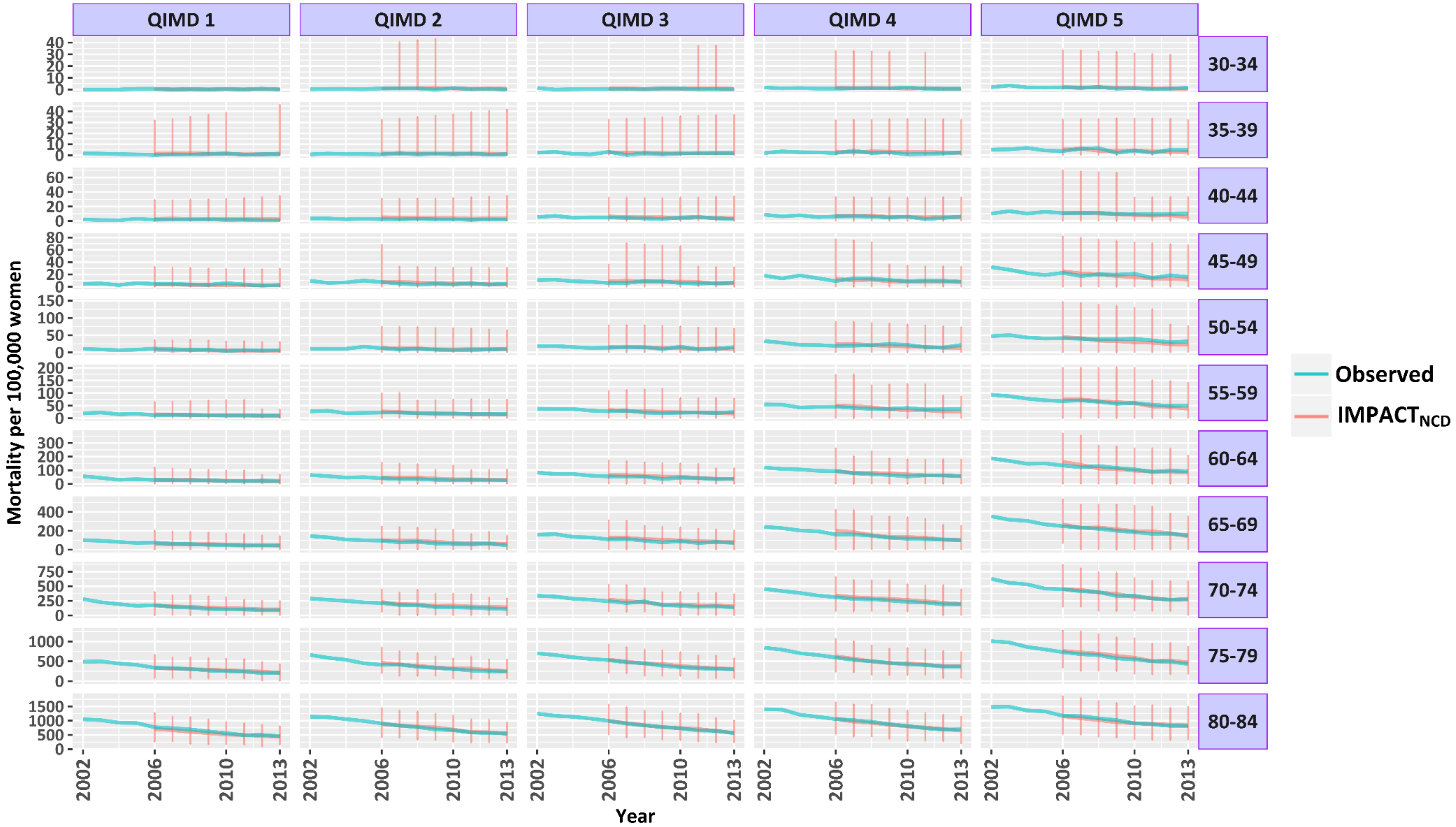

Figure S43 Coronary heart disease mortality (ICD10: I20 – I25) for women by age group and quintile group of index of multiple deprivation (QIMD, 1 = least deprived) between years 2002 and 2013. Observed in the population through mortality registries vs. IMPACT$_{NCD}$ synthetic population estimates. Whiskers represent 95% uncertainty intervals.

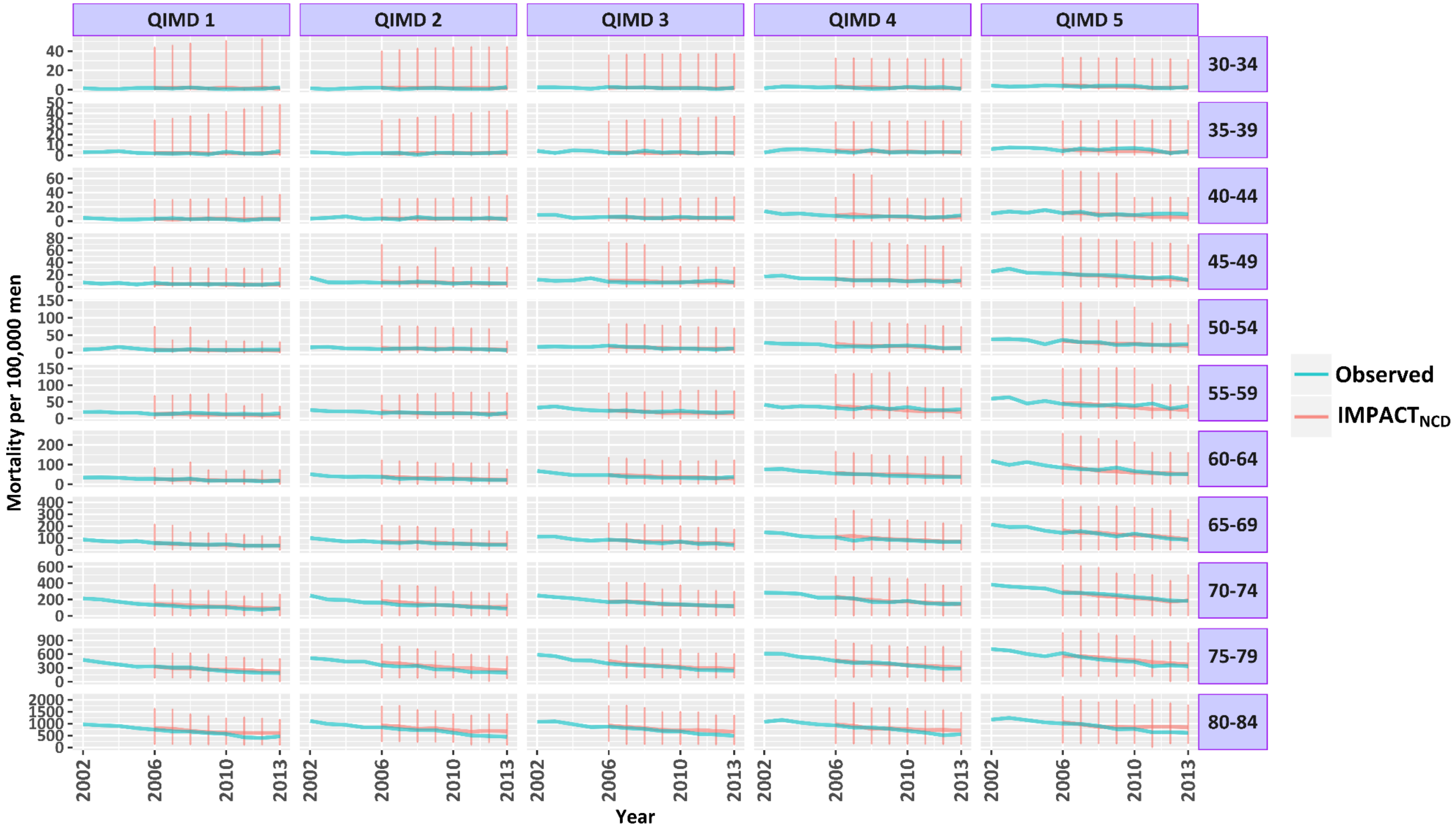

Figure S44 Stroke mortality (ICD10: I60 – I69) for men by age group and quintile group of index of multiple deprivation (QIMD, 1 = least deprived) between years 2002 and 2013. Observed in the population through mortality registries vs. IMPACT$_{NCD}$ synthetic population estimates. Whiskers represent 95% uncertainty intervals.

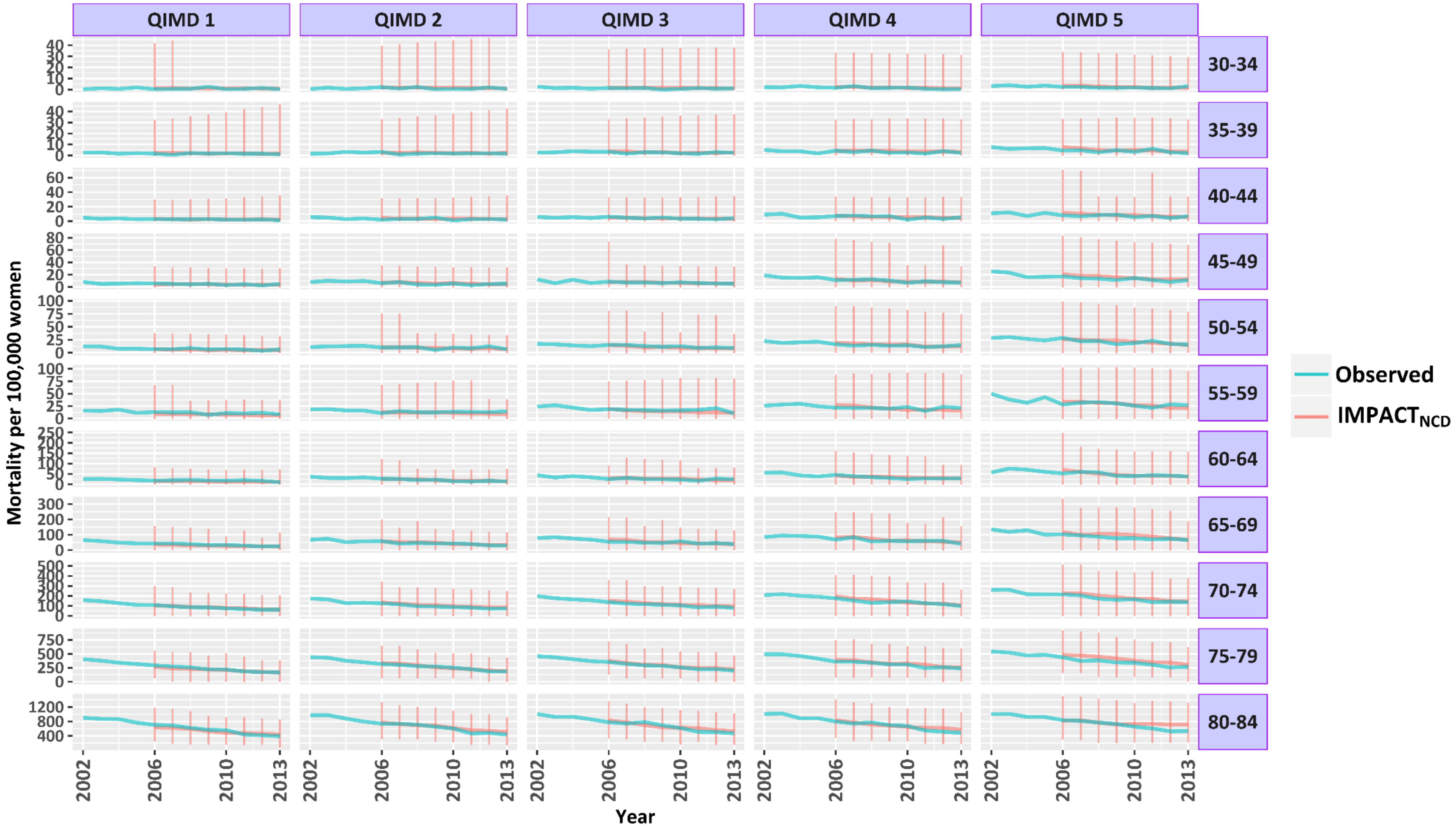

Figure S45 Stroke mortality (ICD10: I60 – I69) for women by age group and quintile group of index of multiple deprivation (QIMD, 1 = least deprived) between years 2002 and 2013. Observed in the population through mortality registries vs. IMPACT$_{NCD}$ synthetic population estimates. Whiskers represent 95% uncertainty intervals.

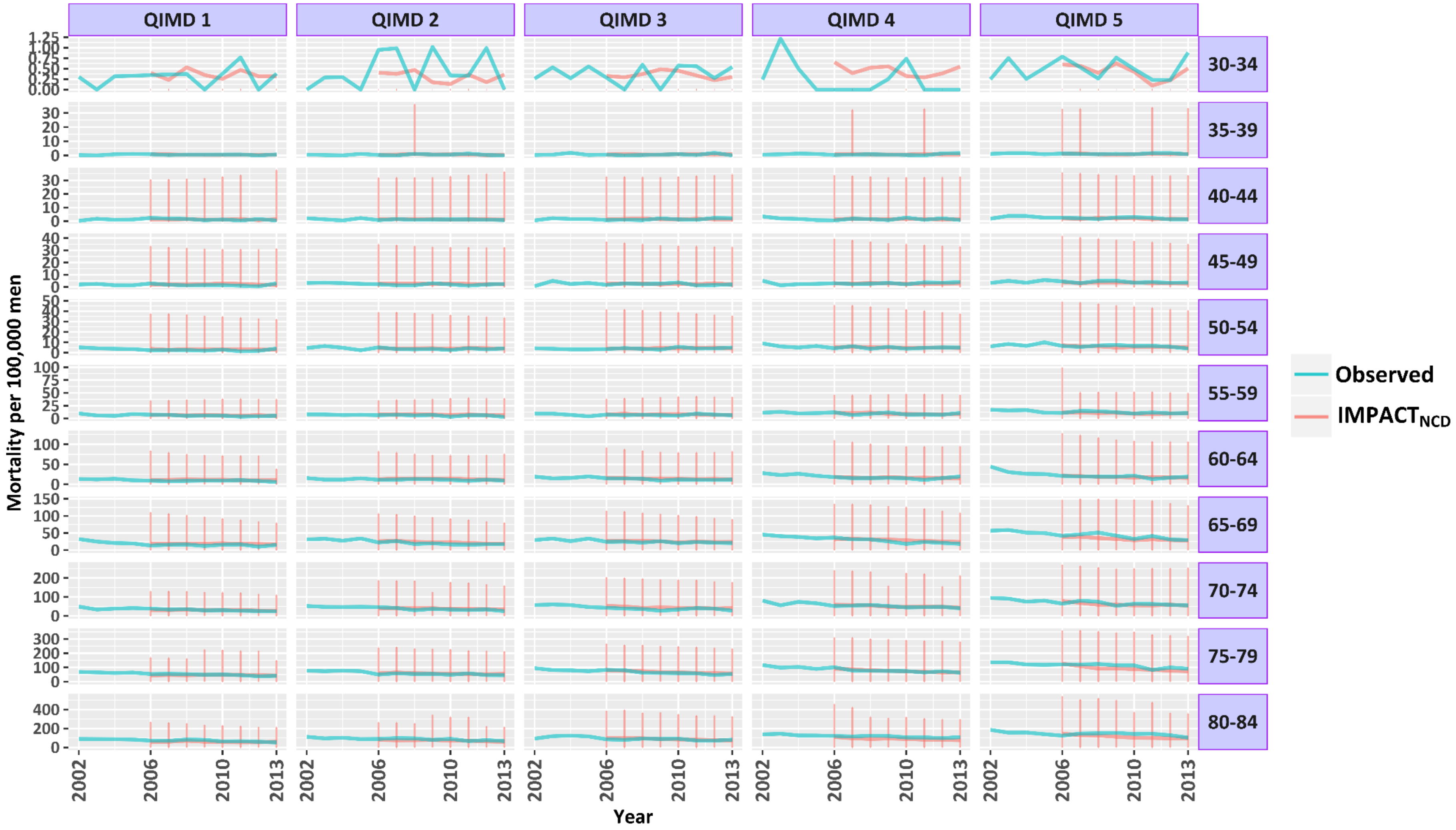

Figure S46 Gastric cancer mortality (ICD10: C16) for men by age group and quintile group of index of multiple deprivation (QIMD, 1 = least deprived) between years 2002 and 2013. Observed in the population through mortality registries vs. IMPACT$_{NCD}$ synthetic population estimates. Whiskers represent 95% uncertainty intervals. Uncertainty intervals could not be estimated for younger age groups due to small number of events.

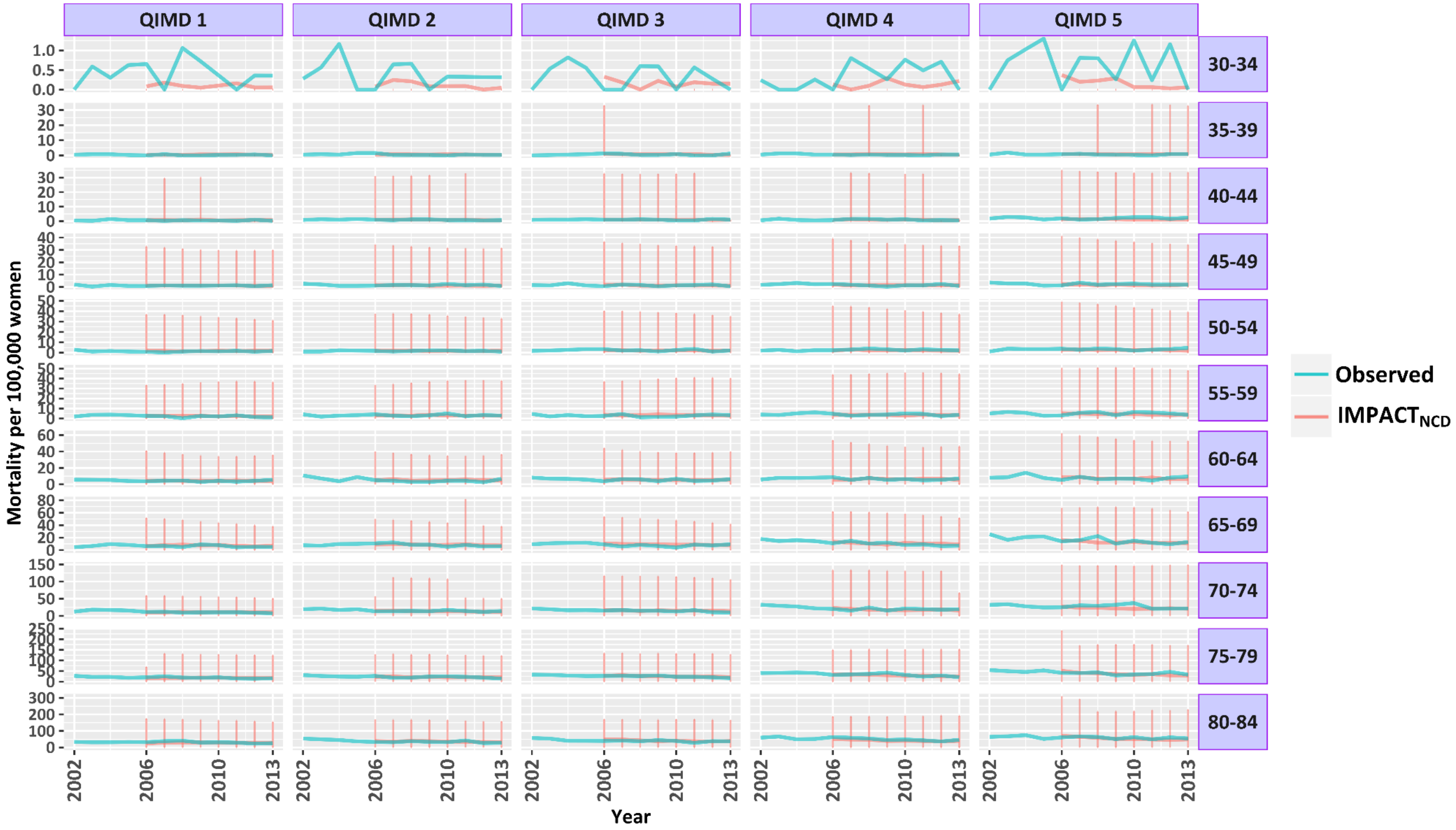

Figure S47 Gastric cancer mortality (ICD10: C16) for women by age group and quintile group of index of multiple deprivation (QIMD, 1 = least deprived) between years 2002 and 2013. Observed in the population through mortality registries vs. IMPACT$_{NCD}$ synthetic population estimates. Whiskers represent 95% uncertainty intervals. Uncertainty intervals could not be estimated for younger age groups due to small number of events.

TABLES

Table S1 IMPACT$_{NCD}$ data sources

Parameter	Outcome	Details	Comments	Source
Fertility rates	Births	Principal-assumption fertility projections for England	Stratified by age	National Population Projections, 2012-based Statistical Bulletin [Internet]. Office for National Statistics; 2013 [cited 2014 Nov 11]. Available from: http://www.ons.gov.uk/ons/rel/npp/national-population-projections/2012-based-projections/index.html
Mortality rates	Deaths from non-modelled causes	Mortality and mid-year population estimates for England	Stratified by age, sex, QIMD and cause of death. Years 2002-2013.	Data requested and obtained by the Office for National Statistics. Available from: http://www.ons.gov.uk/ons/about-ons/business-transparency/freedom-of-information/what-can-i-request/published-ad-hoc-data/health/december-2014/number-of-registered-deaths-by-sex--cause--year--the-adjusted-index.xls
Exposure to risk factors	Exposure of individuals	Health survey for England	Anonymised, individual-level datasets. Years 2001-2012.	Health survey for England 2001-2012. Data available to researchers from http://ukdataservice.ac.uk/
Relative risk for salt consumption	Gastric cancer incidence (ICD10: C16)	Meta-analysis of 2 cohort studies	Both studies adjusted for age, sex, and smoking. One also adjusted for non-green/yellow vegetable intake and the other for education, stomach disorders and history of stomach cancer in the family.	World Cancer Research Fund, American Institute for Cancer Research. Food, nutrition, physical activity, and the prevention of cancer: a global perspective. Washington, DC: WCRF/AICR; 2007. (Figure 4.6.1)

Parameter	Outcome	Details	Comments	Source
Effect of salt consumption on systolic blood pressure	Systolic blood pressure change	Meta-analysis/meta-regression of 103 trials	Only trials with duration > 7 days were analysed.	Mozaffarian D, Fahimi S, Singh GM, Micha R, Khatibzadeh S, Engell RE, et al. Global Sodium Consumption and Death from Cardiovascular Causes. New England Journal of Medicine 2014;371:624–34. (Text S1 in the appendix)
Setting reference level of salt consumption	Ideal salt consumption below which no risk was considered	Evidence from ecologic studies randomised trials and meta-analyses of prospective cohort studies	Intake levels associated with the lowest risk ranged from 1.5 to 6 g/day. The lowest observed mean national intakes were ~3.8 g/day. Thus a PERT (1.5, 3.8, 6) distribution was used.	Mozaffarian D, Fahimi S, Singh GM, Micha R, Khatibzadeh S, Engell RE, et al. Global Sodium Consumption and Death from Cardiovascular Causes. New England Journal of Medicine 2014;371:624–34. (Text S4 in the appendix and Table S3)
Relative risk for active smoking	CHD and stroke (ICD10: I20 – I25 and I60 – I69)	Re-analysis of American Cancer Society's Cancer Prevention Study II. Prospective cohort study, 6 years of follow-up	Stratified by age and sex. Adjusted for age, race, education, marital status, "blue collar" employment in most recent or current job, weekly consumption of vegetables and citrus fruit, vitamin (A, C, and E) use, alcohol use, aspirin use, body mass index, exercise, dietary fat consumption, hypertension and diabetes at baseline.	Ezzati M, Henley SJ, Thun MJ, Lopez AD. Role of Smoking in Global and Regional Cardiovascular Mortality. Circulation 2005;112:489–97. (Table 1 Model B)
	Gastric cancer incidence (ICD10: C16)	EPIC prospective cohort study	Stratified by country. Adjusted for sex, consumption of vegetables, fresh fruits, processed meat, alcohol, body mass index and educational level.	González CA, Pera G, Agudo A, Palli D, Krogh V, Vineis P, et al. Smoking and the risk of gastric cancer in the European Prospective Investigation Into Cancer and Nutrition (EPIC). Int J Cancer 2003;107:629–34. (HR of the $\log_2$ of cigarette-years = 1.040)

Parameter	Outcome	Details	Comments	Source
	Other mortality (except CHD and stroke)	Male British doctors prospective cohort study	Age-standardised	Doll R, Peto R, Boreham J, Sutherland I. Mortality in relation to smoking: 50 years' observations on male British doctors. BMJ 2004;328:1519. (Table 1)
Relative risk for ex-smoking	CHD (ICD10: I20 – I25)	Meta- analysis. Multiple-adjusted pooled estimates from 19 prospective studies	Multiply-adjusted	Huxley RR, Woodward M. Cigarette smoking as a risk factor for coronary heart disease in women compared with men: a systematic review and meta-analysis of prospective cohort studies. The Lancet 2011;378:1297–305. (Web-figure 8)
	Stroke (ICD10 I60 – I69)	The Framingham study. Prospective cohort study	Stroke risk decreased significantly by two years and was at the level of non-smokers by five years after cessation of cigarette smoking.	Wolf PA, D'Agostino RB, Kannel WB, Bonita R, Belanger AJ. Cigarette smoking as a risk factor for stroke: The Framingham study. JAMA 1988;259:1025–9.
	Gastric cancer incidence (ICD10: C16)	EPIC prospective cohort study	Stratified by country. Adjusted for sex, consumption of vegetables, fresh fruits, processed meat, alcohol, body mass index and educational level.	González CA, Pera G, Agudo A, Palli D, Krogh V, Vineis P, et al. Smoking and the risk of gastric cancer in the European Prospective Investigation Into Cancer and Nutrition (EPIC). Int J Cancer 2003;107:629–34. (Table IV. Continuous RR)
Relative risk for environmental tobacco smoking	CHD (ICD10: I20 – I25)	Meta-analysis of 10 cohort and case-control studies	Adjusted for important CHD risk factors.	He J, Vupputuri S, Allen K, Prerost MR, Hughes J, Whelton PK. Passive Smoking and the Risk of Coronary Heart Disease — A Meta-Analysis of Epidemiologic Studies. N Engl J Med 1999;340:920–6. (Table 3. Adjusted RR)

Parameter	Outcome	Details	Comments	Source
	Stroke (ICD10 I60 – I69)	Meta-analysis of 20 prospective, case-control and cross-sectional studies	13 studies adjusted for important CHD risk factors. The overall effect from all 20 studies was used.	Oono IP, Mackay DF, Pell JP. Meta-analysis of the association between second hand smoke exposure and stroke. J Public Health 2011;33:496–502. (Figure 1)
Relative risk for systolic blood pressure	CHD and stroke (ICD10: I20 – I25 and I60 – I69)	Meta-analysis of individual data from 61 prospective studies	Stratified by age and sex. Adjusted for regression dilution and total blood cholesterol and, where available, lipid fractions (HDL and non-HDL cholesterol), diabetes, weight, alcohol consumption, and smoking at baseline.	Age-specific relevance of usual blood pressure to vascular mortality: a meta-analysis of individual data for one million adults in 61 prospective studies. The Lancet 2002;360:1903–13. (Figures 3 and 5)
Relative risk for total cholesterol	CHD and stroke (ICD10: I20 – I25 and I60 – I69)	Meta-analysis of individual data from 61 prospective studies	Stratified by age and sex. Adjusted for regression dilution and age, sex, study, systolic blood pressure and smoking.	Prospective Studies Collaboration. Blood cholesterol and vascular mortality by age, sex, and blood pressure: a meta-analysis of individual data from 61 prospective studies with 55 000 vascular deaths. The Lancet 2007;370:1829–39. (Web-table 6 fully adjusted and Figure 3)
Relative risk for body mass index	CHD and stroke (ICD10: I20 – I25 and I60 – I69)	Meta-analysis of 58 prospective studies	Stratified by age. Adjusted for age, sex, smoking status, systolic blood pressure, history of diabetes, and total and HDL cholesterol.	The Emerging Risk Factors Collaboration. Separate and combined associations of body-mass index and abdominal adiposity with cardiovascular disease: collaborative analysis of 58 prospective studies. The Lancet 2011;377:1085–95. (Table 1 and Figure 2)

Parameter	Outcome	Details	Comments	Source
	Gastric cancer incidence (ICD10: C16)	Meta-analysis of 7 studies	Non-linear dose-response meta-analysis for risk of cardia gastric cancer. Adjusted for age, sex, and smoking.	World Cancer Research Fund International/American Institute for Cancer Research. Continuous Update Project report: diet, nutrition, physical activity and stomach cancer. AICR/WCRF 2016. wcrf.org/stomach-cancer-2016 (Table 8 p37).
Relative risk for diabetes mellitus	CHD and stroke (ICD10: I20 – I25 and I60 – I69)	Meta-analysis of 102 prospective studies	Stratified by age. Adjusted for age, smoking status, body-mass index, and systolic blood pressure.	The Emerging Risk Factors Collaboration. Diabetes mellitus, fasting blood glucose concentration, and risk of vascular disease: a collaborative meta-analysis of 102 prospective studies. The Lancet 2010;375:2215–22. (Figure 2)
	Other mortality (except CHD and stroke)	DECODE. A collaborative prospective study of 22 cohorts in Europe	Adjusted for BMI, blood pressure, smoking and serum cholesterol.	The DECODE Study Group. Is the current definition for diabetes relevant to mortality risk from all causes and cardiovascular and noncardiovascular diseases? Diabetes Care 2003;26:688–96.
Relative risk for physical activity	CHD and stroke (ICD10: I20 – I25 and I60 – I69)	Meta-analysis of 18 cohort studies for CHD and 8 cohort studies for ischaemic stroke	Stratified by age and sex. Adjusted for measurement error, age, sex, smoking, blood pressure and cholesterol.	Bull FC, Armstrong TP, Dixon T, Ham S, Neiman A, Pratt M. Comparative quantification of health risks. Chapter 10: physical inactivity. Geneva: World Health Organisation; 2004. (Tables 10.19 and 10.20)
Relative risk for fruit and vegetable consumption	CHD (ICD10: I20 – I25)	Meta-analysis of 9 cohort studies	RR per portion of F&V. Multiply-adjusted.	Dauchet L, Amouyel P, Hercberg S, Dallongeville J. Fruit and Vegetable Consumption and Risk of Coronary Heart Disease: A Meta-Analysis of Cohort Studies. J Nutr 2006;136:2588–93.
	Stroke (ICD10: I60 – I69)	Meta-analysis of 7 cohort studies	RR per portion of F&V. Multiply-adjusted.	Dauchet L, Amouyel P, Dallongeville J. Fruit and vegetable consumption and risk of stroke A meta-analysis of cohort studies. Neurology 2005;65:1193–7.

Parameter	Outcome	Details	Comments	Source
	Gastric cancer incidence (ICD10: C16)	Reanalysis of the Netherlands Cohort study	Stratified by age group. Estimates are based on the Netherlands Cohort study. Adjusted for age, sex, smoking, education, stomach disorders, and family history of stomach cancer. We considered a risk only for <2 portions/day consumption.[3]	Lock K, Pomerleau J, Causer L, McKee M. Comparative quantification of health risks. Chapter 9: Low fruit and vegetable consumption [Internet]. Geneva: World Health Organisation; 2004. Available from: http://www.who.int/publications/cra/en/ (Table 9.28)

Table S2 IMPACT$_{NCD}$ main assumptions and limitations

Population module	Immigration is not considered.
	Social mobility is not considered.
	Quintile groups of the index of multiple deprivation (QIMD) is a relative marker of (area) deprivation with several versions since 2003. We considered all version of QIMD identical.
	We assume all salt that is consumed is excreted from urine and all urine sodium origins from salt consumption.
	We assume that the surveys used, are truly representative of the population. For example, the adjustments for selection bias in the Health Surveys for England are perfect.
Disease module	We assume multiplicative risk effects.
	We assume log-linear dose-response for the continuous risk factors.
	We assume that the effects of the risk factors on incidence and mortality are equal and risk factors are not modifying survival.
	We assume 5-year mean lag time for CVD and 8-year for GCa (except for the cumulative effect of smoking on GCa where lag was assumed similar to CVD one).
	We assume 100% risk reversibility.
	We assume that trends in disease incidence are attributable only to trends of the relevant modelled risk factors.
	Only well-accepted associations between upstream and downstream risk factors that have been observed in longitudinal studies are considered. However, the magnitudes of the associations are extracted from a series of nationally representative cross-sectional surveys (Health Survey for England).
	For GCa, we assumed that survival 10 years after diagnosis equals remission.

Table S3 Distributions that were used as inputs for the simulations. Numbers are rounded

Variable	Sex	Ages	Distribution
**Relative risks of relevant risk factors for CHD**			
Active smoking[68 table 1 model B]	Men	30 - 44	Log-Normal (mean = ln(5.51), sd = ln(12.3 / 5.51) / 1.96)
		45 - 59	Log-Normal (mean = ln(3.04), sd = ln(3.48 / 3.04) / 1.96)
		60 - 69	Log-Normal (mean = ln(1.88), sd = ln(2.08 / 1.88) / 1.96)
		70 - 79	Log-Normal (mean = ln(1.44), sd = ln(1.63 / 1.44) / 1.96)
	Women	30 - 44	Log-Normal (mean = ln(2.26), sd = ln(6.14 / 2.26) / 1.96)
		45 - 59	Log-Normal (mean = ln(3.78), sd = ln(4.62 / 3.78) / 1.96)
		60 - 69	Log-Normal (mean = ln(2.53), sd = ln(2.87 / 2.53) / 1.96)
		70 - 79	Log-Normal (mean = ln(1.68), sd = ln(1.93 / 1.68) / 1.96)
		80 - 84	Log-Normal (mean = ln(1.38), sd = ln(1.77 / 1.38) / 1.96)
Ex-Smoking[69 web-figure 8]	Men	30 - 84	Log-Normal (mean = ln(1.25), sd = ln(1.32 / 1.25) / 1.96)
	Women	30 - 84	Log-Normal (mean = ln(1.2), sd = ln(1.34 / 1.2) / 1.96)
ETS[70 table 3 adjusted RR]	Both	30 - 84	Log-Normal (mean = ln(1.26), sd = ln(1.38 / 1.26) / 1.96)
SBP[71 figure 5]	Men	30 - 49	Log-Normal (mean = ln(0.5), sd = ln(0.54 / 0.5) / 1.96)
		50 - 59	Log-Normal (mean = ln(0.5), sd = ln(0.52 / 0.5) / 1.96)
		60 - 69	Log-Normal (mean = ln(0.55), sd = ln(0.57 / 0.55) / 1.96)

Variable	Sex	Ages	Distribution
		70 - 74	Log-Normal (mean = ln(0.62), sd = ln(0.64 / 0.62) / 1.96)
		80 - 84	Log-Normal (mean = ln(0.69), sd = ln(0.73 / 0.69) / 1.96)
	Women	30 - 49	Log-Normal (mean = ln(0.4), sd = ln(0.49 / 0.4) / 1.96)
		50 - 59	Log-Normal (mean = ln(0.49), sd = ln(0.54 / 0.49) / 1.96)
		60 - 69	Log-Normal (mean = ln(0.5), sd = ln(0.61 / 0.5) / 1.96)
		70 - 74	Log-Normal (mean = ln(0.55), sd = ln(0.58 / 0.55) / 1.96)
		80 - 84	Log-Normal (mean = ln(0.64), sd = ln(0.68 / 0.64) / 1.96)
TC[72 web-table 6]	Both	30 - 49	Log-Normal (mean = ln(0.49), sd = ln(0.52 / 0.49) / 1.96)
		50 - 59	Log-Normal (mean = ln(0.62), sd = ln(0.65 / 0.62) / 1.96)
		60 - 69	Log-Normal (mean = ln(0.74), sd = ln(0.76 / 0.74) / 1.96)
		70 - 74	Log-Normal (mean = ln(0.84), sd = ln(0.86 / 0.84) / 1.96)
		80 - 84	Log-Normal (mean = ln(0.87), sd = ln(0.9 / 0.87) / 1.96)
BMI[73 table 1 and figure 2]	Both	30 - 59	Log-Normal (mean = ln(1.21), sd = ln(1.28 / 1.21) / 1.96)
		60 - 69	Log-Normal (mean = ln(1.06), sd = ln(1.12 / 1.06) / 1.96)
Diabetes[74 figure 2]	Both	40 - 59	Log-Normal (mean = ln(2.51), sd = ln(2.8/ 2.51) / 1.96)
		60 - 69	Log-Normal (mean = ln(2.01), sd = ln(2.26/ 2.01) / 1.96)
		70 - 84	Log-Normal (mean = ln(1.78), sd = ln(2.05/ 1.78) / 1.96)

Variable	Sex	Ages	Distribution
PA[75 table 10.19]	Both	30 - 69	No active days: Log-Normal (mean = ln(1.71), sd = ln(1.85/ 1.71) / 1.96)
			1 – 4 active days: Log-Normal (mean = ln(1.44), sd = ln(1.62/ 1.44) / 1.96)
		70 - 79	No active days: Log-Normal (mean = ln(1.5), sd = ln(1.61/ 1.5) / 1.96)
			1 – 4 active days: Log-Normal (mean = ln(1.31), sd = ln(1.48/ 1.31) / 1.96)
		80 - 84	No active days: Log-Normal (mean = ln(1.4), sd = ln(1.41/ 1.4) / 1.96)
			1 – 4 active days: Log-Normal (mean = ln(1.2), sd = ln(1.35/ 1.2) / 1.96)
F&V[76]			Log-Normal (mean = ln(0.96), sd = ln(1.0.99/ 0.96) / 1.96)
**Relative risks of relevant risk factors for stroke**			
Active smoking[68 table 1 model B]	Men	30 - 59	Log-Normal (mean = ln(3.12), sd = ln(4.64 / 3.12) / 1.96)
		60 - 69	Log-Normal (mean = ln(1.87), sd = ln(2.44 / 1.87) / 1.96)
		70 - 79	Log-Normal (mean = ln(1.39), sd = ln(1.77 / 1.39) / 1.96)
	Women	30 - 59	Log-Normal (mean = ln(4.61), sd = ln(6.37 / 4.61) / 1.96)
		60 - 69	Log-Normal (mean = ln(2.81), sd = ln(3.58 / 2.81) / 1.96)
		70 - 79	Log-Normal (mean = ln(1.95), sd = ln(2.45 / 1.95) / 1.96)
ETS[77 figure 1]	Both	30 - 84	Log-Normal (mean = ln(1.25), sd = ln(1.38 / 1.25) / 1.96)
SBP[71 figure 3]	Men	30 - 49	Log-Normal (mean = ln(0.33), sd = ln(0.38 / 0.33) / 1.96)
		50 - 59	Log-Normal (mean = ln(0.34), sd = ln(0.37 / 0.34) / 1.96)

Variable	Sex	Ages	Distribution
		60 - 69	Log-Normal (mean = ln(0.41), sd = ln(0.44 / 0.41) / 1.96)
		70 - 74	Log-Normal (mean = ln(0.48), sd = ln(0.51 / 0.48) / 1.96)
		80 - 84	Log-Normal (mean = ln(0.68), sd = ln(0.75 / 0.68) / 1.96)
	Women	30 - 49	Log-Normal (mean = ln(0.41), sd = ln(0.49 / 0.41) / 1.96)
		50 - 59	Log-Normal (mean = ln(0.45), sd = ln(0.5 / 0.45) / 1.96)
		60 - 69	Log-Normal (mean = ln(0.47), sd = ln(0.51 / 0.47) / 1.96)
		70 - 74	Log-Normal (mean = ln(0.53), sd = ln(0.56 / 0.53) / 1.96)
		80 - 84	Log-Normal (mean = ln(0.65), sd = ln(0.71 / 0.65) / 1.96)
TC[72 figure 3]	Both	40 - 49	Log-Normal (mean = ln(0.87), sd = ln(1 / 0.87) / 1.96)
		50 - 59	Log-Normal (mean = ln(0.91), sd = ln(0.97 / 0.91) / 1.96)
		60 - 69	Log-Normal (mean = ln(0.93), sd = ln(0.97 / 0.93) / 1.96)
BMI[73 table 1 and figure 2]	Both	30 - 59	Log-Normal (mean = ln(1.18), sd = ln(1.26 / 1.18) / 1.96)
		60 - 69	Log-Normal (mean = ln(1.08), sd = ln(1.15 / 1.08) / 1.96)
Diabetes[74 figure 2]	Both	40 - 59	Log-Normal (mean = ln(3.74), sd = ln(4.58/ 3.74) / 1.96)
		60 - 69	Log-Normal (mean = ln(2.06), sd = ln(2.58/ 2.06) / 1.96)
		70 - 84	Log-Normal (mean = ln(1.8), sd = ln(2.27/ 1.8) / 1.96)
PA[75 table 10.20]	Both	30 - 69	No active days: Log-Normal (mean = ln(1.53), sd = ln(1.79/ 1.53 / 1.96)

Variable	Sex	Ages	Distribution
		70 - 79	No active days: Log-Normal (mean = ln(1.38), sd = ln(1.6/ 1.38) / 1.96)
		80 - 84	No active days: Log-Normal (mean = ln(1.24), sd = ln(1.45/ 1.24) / 1.96)
F&V[78]			Log-Normal (mean = ln(0.95), sd = ln(0.97/ 0.95) / 1.96)
**Relative risks of relevant risk factors for GCa**			
Active smoking (duration in years)[86 table III]	Both	30 - 84	Normal (mean = 0.03, sd = 0.002)
Ex-smoking (years since cessation)[86 table IV]	Both	30 - 84	Log-Normal (mean = ln(0.96), sd = ln(1/ 0.96) / 1.96)
BMI[3 table 8]	Both	30 - 84	Normal (mean and sd is a function of BMI)
F&V[87 table 9.28]	Both	30 - 69	Log-Normal (mean = ln(0.94), sd = ln(1/ 0.94) / 1.96)
	Both	70 - 79	Log-Normal (mean = ln(0.96), sd = ln(1/ 0.96) / 1.96)
	Both	80 - 84	Log-Normal (mean = ln(0.97), sd = ln(1/ 0.97) / 1.96)
Salt[88]	Both	30 - 84	Log-Normal (mean = ln(1.08), sd = ln(1.08/ 1) / 1.96)
**Other inputs**			
CVD lag time	Both	30 - 84	1 + Binomial(n = 9, p = (5-1)/9)
GCa lag time	Both	30 - 84	1 + Binomial(n = 9, p = (8-1)/9)
Optimal salt consumption[5 appendix Text S4]	Both	30 - 84	PERT(min = 1.5, mode = 3.8, max = 6, shape = 4)
Stricter salt policy target	Both	30 - 84	PERT(min = 5.8, mode = 6, max = 7, shape = 4)

REFERENCES

1. Strazzullo P, D'Elia L, Kandala N-B, Cappuccio FP. Salt intake, stroke, and cardiovascular disease: meta-analysis of prospective studies. BMJ 2009;339:b4567.

2. D'Elia L, Rossi G, Ippolito R, Cappuccio FP, Strazzullo P. Habitual salt intake and risk of gastric cancer: A meta-analysis of prospective studies. Clin Nutr 2012;31:489–98.

3. World Cancer Research Fund International/American Institute for Cancer Research. Continuous Update Project report: diet, nutrition, physical activity and stomach cancer [Internet]. AICR/WCRF; 2016 [cited 2016 Apr 22]. Available from: http://wcrf.org/sites/default/files/Stomach-Cancer-2016-Report.pdf

4. He FJ, Li J, MacGregor GA. Effect of longer-term modest salt reduction on blood pressure [Internet]. In: Cochrane Database of Systematic Reviews. John Wiley & Sons, Ltd; 2013 [cited 2015 May 27]. Available from: http://onlinelibrary.wiley.com/doi/10.1002/14651858.CD004937.pub2/abstract

5. Mozaffarian D, Fahimi S, Singh GM, Micha R, Khatibzadeh S, Engell RE, et al. Global sodium consumption and death from cardiovascular causes. N Engl J Med 2014;371:624–34.

6. Tatematsu M, Takahashi M, Fukushima S, Hananouchi M, Shirai T. Effects in rats of sodium chloride on experimental gastric cancers induced by N-methyl-N-nitro-N-nitrosoguanidine or 4-nitroquinoline-1-oxide. J Natl Cancer Inst 1975;55:101–6.

7. Fox J, Dangler C, Taylor N, King A, Koh T, Wang T. High-salt diet induces gastric epithelial hyperplasia and parietal cell loss, and enhances Helicobacter pylori colonization in C57BL/6 mice. CANCER Res 1999;59:4823–8.

8. Ioannidis JP. Commentary: Salt and the assault of opinion on evidence. Int J Epidemiol 2016;45:264–5.

9. World Health Organisation. Guideline: Sodium Intake for Adults and Children [Internet]. Geneva: World Health Organisation; 2012 [cited 2016 Jul 17]. Available from: http://www.ncbi.nlm.nih.gov/books/NBK133309/

10. Scientific Advisory Committee on Nutrition. Salt and health [Internet]. Norwich, UK: The stationery office; 2003 [cited 2016 Nov 14]. Available from: https://www.gov.uk/government/uploads/system/uploads/attachment_data/file/338782/SACN_Salt_and_Health_report.pdf

11. Mente A, O'Donnell M, Rangarajan S, Dagenais G, Lear S, McQueen M, et al. Associations of urinary sodium excretion with cardiovascular events in individuals with and without hypertension: a pooled analysis of data from four studies. The Lancet 2016;388:465–75.

12. O'Donnell M, Mente A, Rangarajan S, McQueen MJ, Wang X, Liu L, et al. Urinary sodium and potassium excretion, mortality, and cardiovascular events. N Engl J Med 2014;371:612–23.

13. Cogswell ME, Mugavero K, Bowman BA, Frieden TR. Dietary sodium and cardiovascular disease risk — measurement matters. N Engl J Med 2016;375:580--586.

14. Taylor R, Najafi F, Dobson A. Meta-analysis of studies of passive smoking and lung cancer: effects of study type and continent. Int J Epidemiol 2007;36:1048–59.

15. Aburto NJ, Ziolkovska A, Hooper L, Elliott P, Cappuccio FP, Meerpohl JJ. Effect of lower sodium intake on health: systematic review and meta-analyses. BMJ 2013;346:f1326.

16. Ballard-Barbash R, Hunsberger S, Alciati MH, Blair SN, Goodwin PJ, McTiernan A, et al. Physical activity, weight control, and breast cancer risk and survival: clinical trial rationale and design considerations. JNCI J Natl Cancer Inst 2009;101:630–43.

17. Williamson P. The role of the International Journal of Microsimulation. Int J Microsimulation 2007;1:1–2.

18. Zucchelli E, Jones AM, Rice N. The evaluation of health policies through dynamic microsimulation methods. Int J Microsimulation 2012;5:2–20.

19. Department for Communities and Local Government. English indices of deprivation 2010 - Publications - GOV.UK [Internet]. 2011 [cited 2014 Aug 26];Available from: https://www.gov.uk/government/statistics/english-indices-of-deprivation-2010

20. R Core Team. R: a language and environment for statistical computing [Internet]. R Foundation for Statistical Computing; 2014. Available from: http://www.R-project.org/

21. Dowle M, Short T, Lianoglou S, Srinivasan A. data.table: Extension of data.frame [Internet]. 2015. Available from: https://github.com/Rdatatable/data.table/

22. Revolution Analytics, Weston S. foreach: Foreach looping construct for R [Internet]. 2014. Available from: http://CRAN.R-project.org/package=foreach

23. Gaujoux R. doRNG: Generic Reproducible Parallel Backend for foreach Loops [Internet]. 2014. Available from: http://CRAN.R-project.org/package=doRNG

24. L'Ecuyer P. Good Parameters and Implementations for Combined Multiple Recursive Random Number Generators. Oper Res 1999;47:159–64.

25. Alfons A, Kraft S, Templ M, Filzmoser P. Simulation of close-to-reality population data for household surveys with application to EU-SILC. Stat Methods Appl 2011;20:383–407.

26. Dahlgren G, Whitehead M. Policies and strategies to promote social equity in health [Internet]. Copenhagen: World Health Organisation; 1992 [cited 2015 May 5]. Available from: http://core.ac.uk/download/pdf/6472456.pdf

27. National Centre for Social Research, University College London. Department of Epidemiology and Public Health. Health Survey for England, 2001 [computer file]. 3rd Edition. Colchester, Essex: UK Data Archive [distributor] [Internet]. 2010 [cited 2014 May 1];Available from: http://dx.doi.org/10.5255/UKDA-SN-4628-1

28. Alfons A, Kraft S. simPopulation: simulation of synthetic populations for surveys based on sample data [Internet]. 2013. Available from: http://CRAN.R-project.org/package=simPopulation

29. Giles Horsfield SSD. Chapter 3: Equivalised Income [Internet]. Off. Natl. Stat.2012 [cited 2015 May 4];Available from: http://www.ons.gov.uk/ons/rel/family-spending/family-spending/family-spending-2012-edition/art-chapter-3--equivalised-income.html

30. Economic Labour and Social Analysis Office for National Statistics. SOC2010 volume 3: the National Statistics socio-economic classification (NS-SEC rebased on the SOC2010) [Internet]. Off. Natl. Stat.2010 [cited 2015 Jan 6];Available from: http://www.ons.gov.uk/ons/guide-method/classifications/current-standard-classifications/soc2010/soc2010-volume-3-ns-sec--rebased-on-soc2010--user-manual/index.html

31. Ji C, Sykes L, Paul C, Dary O, Legetic B, Campbell NRC, et al. Systematic review of studies comparing 24-hour and spot urine collections for estimating population salt intake. Rev Panam Salud Pública 2012;32:307–15.

32. Ji C, Miller MA, Venezia A, Strazzullo P, Cappuccio FP. Comparisons of spot vs 24-h urine samples for estimating population salt intake: Validation study in two independent samples of adults in Britain and Italy. Nutr Metab Cardiovasc Dis 2014;24:140–7.

33. Mulrow CD, Chiquette E, Angel L, Cornell J, Summerbell C, Anagnostelis B, et al. Dieting to reduce body weight for controlling hypertension in adults. Cochrane Database Syst Rev 2000;CD000484.

34. Goldstein DJ. Beneficial health effects of modest weight loss. Int J Obes Relat Metab Disord J Int Assoc Study Obes 1992;16:397–415.

35. MacMahon S, Cutler J, Brittain E, Higgins M. Obesity and hypertension: epidemiological and clinical issues. Eur Heart J 1987;8 Suppl B:57–70.

36. Dyer AR, Elliott P. The INTERSALT study: relations of body mass index to blood pressure. INTERSALT Co-operative Research Group. J Hum Hypertens 1989;3:299–308.

37. Dattilo AM, Kris-Etherton PM. Effects of weight reduction on blood lipids and lipoproteins: a meta-analysis. Am J Clin Nutr 1992;56:320–8.

38. Kahn SE, Hull RL, Utzschneider KM. Mechanisms linking obesity to insulin resistance and type 2 diabetes. Nature 2006;444:840–6.

39. Lahti-Koski M, Pietinen P, Heliövaara M, Vartiainen E. Associations of body mass index and obesity with physical activity, food choices, alcohol intake, and smoking in the 1982–1997 FINRISK Studies. Am J Clin Nutr 2002;75:809–17.

40. DiPietro L. Physical activity in the prevention of obesity: current evidence and research issues. Med Sci Sports Exerc 1999;31:S542-546.

41. Fogelholm M, Kukkonen-Harjula K. Does physical activity prevent weight gain--a systematic review. Obes Rev Off J Int Assoc Study Obes 2000;1:95–111.

42. Dochi M, Sakata K, Oishi M, Tanaka K, Kobayashi E, Suwazono Y. Smoking as an independent risk factor for hypertension: a 14-year longitudinal study in male Japanese workers. Tohoku J Exp Med 2009;217:37–43.

43. Bowman TS, Gaziano JM, Buring JE, Sesso HD. A Prospective Study of Cigarette Smoking and Risk of Incident Hypertension in Women. J Am Coll Cardiol 2007;50:2085–92.

44. National Centre for Social Research, University College London. Department of Epidemiology and Public Health. Health Survey for England, 2002 [computer file]. 2nd Edition. Colchester, Essex: UK Data Archive [distributor] [Internet]. 2010 [cited 2014 May 1];Available from: http://dx.doi.org/10.5255/UKDA-SN-4912-1

45. National Centre for Social Research, University College London. Department of Epidemiology and Public Health. Health Survey for England, 2003 [computer file]. 2nd Edition. Colchester, Essex: UK Data Archive [distributor] [Internet]. 2010 [cited 2014 May 1];Available from: http://dx.doi.org/10.5255/UKDA-SN-5098-1

46. National Centre for Social Research, University College London. Department of Epidemiology and Public Health. Health Survey for England, 2004 [computer file]. 2nd Edition. Colchester, Essex: UK Data Archive [distributor] [Internet]. 2010 [cited 2014 May 1];Available from: http://dx.doi.org/10.5255/UKDA-SN-5439-1

47. National Centre for Social Research, University College London. Department of Epidemiology and Public Health. Health Survey for England, 2005 [computer file]. 3rd Edition. Colchester, Essex: UK Data Archive [distributor] [Internet]. 2011 [cited 2014 May 1];Available from: http://dx.doi.org/10.5255/UKDA-SN-5675-1

48. National Centre for Social Research, University College London. Department of Epidemiology and Public Health. Health Survey for England, 2006 [computer file]. 4th Edition. Colchester, Essex: UK Data Archive [distributor] [Internet]. 2011 [cited 2014 May 1];Available from: http://dx.doi.org/10.5255/UKDA-SN-5809-1

49. National Centre for Social Research, University College London. Department of Epidemiology and Public Health. Health Survey for England, 2007 [computer file]. 2nd Edition. Colchester, Essex: UK Data Archive [distributor] [Internet]. 2010 [cited 2014 May 1];Available from: http://dx.doi.org/10.5255/UKDA-SN-6112-1

50. National Centre for Social Research, University College London. Department of Epidemiology and Public Health. Health Survey for England, 2008 [computer file]. 4th Edition. Colchester, Essex: UK Data Archive [distributor] [Internet]. 2013 [cited 2014 May 1];Available from: http://dx.doi.org/10.5255/UKDA-SN-6397-2

51. National Centre for Social Research, University College London. Department of Epidemiology and Public Health. Health Survey for England, 2009 [computer file]. 2nd Edition. Colchester, Essex: UK Data Archive [distributor] [Internet]. 2011 [cited 2014 May 1];Available from: http://dx.doi.org/10.5255/UKDA-SN-6732-1

52. NatCen Social Research, Free Royal, University College London. Department of Epidemiology and Public Health. Health Survey for England, 2010 [computer file]. 2nd Edition. Colchester, Essex: UK Data Archive [distributor] [Internet]. 2012 [cited 2014 May 1];Available from: http://dx.doi.org/10.5255/UKDA-SN-6986-2

53. NatCen Social Research, University College London. Department of Epidemiology and Public Health. Health Survey for England, 2011 [computer file]. Colchester, Essex: UK Data Archive [distributor] [Internet]. 2013 [cited 2014 May 1];Available from: http://dx.doi.org/10.5255/UKDA-SN-7260-1

54. NatCen Social Research, University College London. Department of Epidemiology and Public Health. Health Survey for England, 2012 [computer file]. Colchester, Essex: UK Data Archive [distributor] [Internet]. 2014 [cited 2014 May 1];Available from: http://dx.doi.org/10.5255/UKDA-SN-7480-1

55. Sadler K, Nicholson S, Steer T, Gill V, Bates B, Tipping S, et al. National Diet and Nutrition Survey - Assessment of dietary sodium in adults (aged 19 to 64 years) in England, 2011 [Internet]. London: Department of Health; 2012 [cited 2016 Nov 14]. Available from:

http://webarchive.nationalarchives.gov.uk/20130402145952/http://transparency.dh.gov.uk/2012/06/21/sodium-levels-among-adults/

56. National Centre for Social Research. An assessment of dietary sodium levels among adults (aged 19-64) in the UK general population in 2008, based on analysis of dietary sodium in 24-hour urine samples [Internet]. 2008. Available from: http://tna.europarchive.org/20110116113217/http://www.food.gov.uk/multimedia/pdfs/08sodiumreport.pdf

57. National Centre for Social Research. An assessment of dietary sodium levels among adults (aged 19-64) in the general population in England, based on analysis of dietary sodium in 24-hour urine samples [Internet]. 2006 [cited 2016 Nov 14]. Available from: http://tna.europarchive.org/20110116113217/http://www.food.gov.uk/multimedia/pdfs/englandsodiumreport.pdf

58. Henderson L, Irving K, Gregory J, Bates CJ, Prentice A, Perks J, et al. National Diet and Nutrition Survey: adults aged 19 to 64 years. Volume 3: Vitamin and mineral intake and urinary analytes. [Internet]. London: The Stationery Office; 2003 [cited 2015 Jun 5]. Available from: http://www.ons.gov.uk/ons/rel/lifestyles/the-national-diet-and-nutrition-survey/2001-edition/the-national-diet-and-nutrition-survey---volume-3.pdf

59. Belgorodski N, Greiner M, Tolksdorf K, Schueller K. rriskDistributions: Fitting Distributions to Given Data or Known Quantiles [Internet]. 2015. Available from: http://CRAN.R-project.org/package=rriskDistributions

60. Hippisley-Cox J, Coupland C, Robson J, Sheikh A, Brindle P. Predicting risk of type 2 diabetes in England and Wales: prospective derivation and validation of QDScore. BMJ 2009;338:b880.

61. Capewell S, O'Flaherty M. Rapid mortality falls after risk-factor changes in populations. The Lancet 2011;378:752–3.

62. Capewell S, O'Flaherty M. Can dietary changes rapidly decrease cardiovascular mortality rates? Eur Heart J 2011;32:1187–9.

63. Comparative quantification of health risks [Internet]. Geneva: World Health Organisation; 2004 [cited 2014 Jan 30]. Available from: http://www.who.int/publications/cra/en/

64. National Population Projections, 2012-based Statistical Bulletin [Internet]. Office for National Statistics; 2013 [cited 2014 Nov 11]. Available from: http://www.ons.gov.uk/ons/rel/npp/national-population-projections/2012-based-projections/index.html

65. Levin ML. The occurrence of lung cancer in man. Acta - Unio Int Contra Cancrum 1953;9:531–41.

66. Office for National Statistics. Published ad hoc data: health, requests during December 2014 [Internet]. Publ. Ad Hoc Data Health Req. Dec. 20142014 [cited 2015 Oct 14];Available from: http://www.ons.gov.uk/ons/about-ons/business-transparency/freedom-of-information/what-can-i-request/published-ad-hoc-data/health/december-2014/number-of-registered-deaths-by-sex--cause--year--the-adjusted-index.xls

67. Townsend N, Wickramasinghe K, Bhatnagar P, Smolina K, Nichols M, Leal J, et al. Coronary heart disease statistics 2012 edition [Internet]. British Heart Foundation; 2012 [cited 2016 Nov 14].

Available from: https://www.bhf.org.uk/-/media/files/publications/research/2012_chd_statistics_compendium.pdf

68. Smolina K, Wright FL, Rayner M, Goldacre MJ. Incidence and 30-day case fatality for acute myocardial infarction in England in 2010: national-linked database study. Eur J Public Health 2012;22:848–53.

69. Barendregt JJ, van Oortmarssen GJ, Vos T, Murray CJ. A generic model for the assessment of disease epidemiology: the computational basis of DisMod II. Popul Health Metr 2003;1:4.

70. Lim SS, Vos T, Flaxman AD, Danaei G, Shibuya K, Adair-Rohani H, et al. A comparative risk assessment of burden of disease and injury attributable to 67 risk factors and risk factor clusters in 21 regions, 1990–2010: a systematic analysis for the Global Burden of Disease Study 2010. The Lancet 2012;380:2224–60.

71. Boshuizen HC, Lhachimi SK, Baal PHM van, Hoogenveen RT, Smit HA, Mackenbach JP, et al. The DYNAMO-HIA Model: An Efficient Implementation of a Risk Factor/Chronic Disease Markov Model for Use in Health Impact Assessment (HIA). Demography 2012;49:1259–83.

72. Office for National Statistics. Cancer registrations in England [Internet]. Cancer Regist. Engl.2015 [cited 2015 Sep 28];Available from: http://www.ons.gov.uk/ons/rel/vsob1/cancer-registrations-in-england/index.html

73. Office for National Statistics. Cancer survival rates - cancer survival in England: patients diagnosed 2006–2010 and followed up to 2011 [Internet]. Cancer Surviv. Rates - Cancer Surviv. Engl. Patients Diagn.2012 [cited 2015 Sep 28];Available from: http://www.ons.gov.uk/ons/rel/cancer-unit/cancer-survival/2006---2010--followed-up-to-2011/index.html

74. Hyndman RJ. demography: Forecasting mortality, fertility, migration and population data [Internet]. 2014. Available from: http://CRAN.R-project.org/package=demography

75. Hyndman RJ, Shahid Ullah M. Robust forecasting of mortality and fertility rates: A functional data approach. Comput Stat Data Anal 2007;51:4942–56.

76. Doll R, Peto R, Boreham J, Sutherland I. Mortality in relation to smoking: 50 years’ observations on male British doctors. BMJ 2004;328:1519.

77. The DECODE Study Group. Is the current definition for diabetes relevant to mortality risk from all causes and cardiovascular and noncardiovascular diseases? Diabetes Care 2003;26:688–96.

78. Stephens MR, Blackshaw GRJC, Lewis WG, Edwards P, Barry JD, Hopper NA, et al. Influence of socio-economic deprivation on outcomes for patients diagnosed with gastric cancer. Scand J Gastroenterol 2005;40:1351–7.

79. Siemerink EJM, Hospers GAP, Mulder NH, Siesling S, van der Aa MA. Disparities in survival of stomach cancer among different socioeconomic groups in North-East Netherlands. Cancer Epidemiol 2011;35:413–6.

80. Allemani C, Weir HK, Carreira H, Harewood R, Spika D, Wang X-S, et al. Global surveillance of cancer survival 1995–2009: analysis of individual data for 25 676 887 patients from 279 population-based registries in 67 countries (CONCORD-2). The Lancet 2015;385:977–1010.

81. Koerkamp BG, Stijnen T, Weinstein MC, Hunink MGM. The combined analysis of uncertainty and patient heterogeneity in medical decision models. Med Decis Making 2011;31:650–61.

82. Briggs AH, Weinstein MC, Fenwick EAL, Karnon J, Sculpher MJ, Paltiel AD. Model parameter estimation and uncertainty: a report of the ISPOR-SMDM modeling good research practices Task Force-6. Value Health 2012;15:835–42.

83. Mackenbach JP, Kunst AE. Measuring the magnitude of socio-economic inequalities in health: An overview of available measures illustrated with two examples from Europe. Soc Sci Med 1997;44:757–71.

84. Bross IDJ. How to use ridit analysis. Biometrics 1958;14:18–38.

85. Friendly M. Mosaic Displays for Multi-Way Contingency Tables. J Am Stat Assoc 1994;89:190–200.

86. González CA, Pera G, Agudo A, Palli D, Krogh V, Vineis P, et al. Smoking and the risk of gastric cancer in the European Prospective Investigation Into Cancer and Nutrition (EPIC). Int J Cancer 2003;107:629–34.

87. Lock K, Pomerleau J, Causer L, McKee M. Comparative quantification of health risks. Chapter 9: low fruit and vegetable consumption [Internet]. Geneva: World Health Organisation; 2004. Available from: http://www.who.int/publications/cra/en/

88. World Cancer Research Fund, American Institute for Cancer Research. Food, nutrition, physical activity, and the prevention of cancer: a global perspective. Washington, DC: WCRF/AICR; 2007.
